# Synthesis, Cytotoxicity and Antimicrobial Action of 11 Analogs of Nifuroxazide

**DOI:** 10.3390/ijms27187983

**Published:** 2026-09-08

**Authors:** Daniil N. Yarullin, Olga I. Logacheva, Aleksandra K. Isagulieva, Olga N. Sineva, Vera S. Sadykova, Maksim N. Zavalishin, George A. Gamov

**Affiliations:** 1 General Chemical Technology Department, Ivanovo State University of Chemistry and Technology, Sheremetevskii Ave. 7, 153000 Ivanovo, Russia; dn_yarullin@isuct.ru (D.N.Y.); o.logachiova@yandex.ru (O.I.L.); ggamov@isuct.ru (G.A.G.); 2 Burnazyan Federal Medical Biophysical Center, Federal Medical Biological Agency, Marshal Novikov St., 23, 123098 Moscow, Russia; kia2303@yandex.ru; 3 Gause Institute of New Antibiotics, Bol’shaya Pirogovskaya St. 11/1, 119021 Moscow, Russia; olga.sineva81@yandex.ru (O.N.S.); sadykova_09@mail.ru (V.S.S.)

**Keywords:** nifuroxazide, hydrazone, antibiotic, cytotoxicity, protein binding

## Abstract

The global spread of multidrug-resistant bacteria, alongside the rising burden of oncological diseases, poses an increasing threat to human health and public healthcare systems worldwide. Substantial efforts are underway to discover new drugs and to reposition or repurpose existing ones. Recently, nifuroxazide introduced into clinical practice in the late 1960s has attracted considerable attention due to its antitumor potential. However, its poor absorption in the intestinal tract limits its applicability as a systemic antitumor agent, a limitation that can be addressed through chemical modification. In this study, we evaluated 11 nifuroxazide analogs featuring systematic modifications to both the five-membered ring (replacement of furan with thiophene) and the six-membered aromatic ring (variation in the position and nature of substituents). MTT cytotoxicity assays performed on HCT116 and HEK293 cell lines revealed that 5-nitrofurfurylidene hydrazide of 2-pyridinecarboxylic acid and 5-nitrothien-2-yl hydrazide of 2-pyridinecarboxylic acid exhibit selective toxicity toward tumor cells while showing significantly lower toxicity to non-tumor cells, indicating potential for further investigation in more complex biological models. Antimicrobial activity testing identified 5-nitrofurfurylidene hydrazide of 3-hydroxybenzoic acid and 5-nitrofurfurylidene hydrazide of 3-pyridinecarboxylic acid as particularly potent.

## 1. Introduction

The increasing dissemination of multidrug-resistant microorganisms constitutes a critical threat to global public health [1,2]. In 2021, an estimated 4.71 million deaths were associated with bacterial antibiotic resistance, and this figure is projected to rise to 8.22 million by 2050 [1]. In response, numerous nations have implemented strategic programs to curb antimicrobial resistance (Russia, for instance, has adopted the “Strategy for Preventing the Spread of Antimicrobial Resistance” [3]). A key component of these national efforts is the discovery and development of novel antimicrobial agents, with drug repurposing and structural modification of existing pharmaceuticals representing particularly promising approaches.

In this context, nifuroxazide (5-nitrofurfurylidene hydrazide of 4-hydroxybenzoic acid) has attracted considerable attention. Patented in the United States in 1966 [4] and widely used for the treatment of acute diarrhea, vaginitis, and infections caused by *Chlamydia trachomatis*, *Mycoplasma*, and *Candida* [5,6], nifuroxazide exhibits broad-spectrum antibacterial activity against Gram-positive bacteria (e.g., *Staphylococcus* spp.) and Gram-negative pathogens, including *Escherichia*, *Salmonella*, *Enterobacter*, *Yersinia*, and *Klebsiella* spp. Notably, it lacks efficacy against *Pseudomonas aeruginosa*, *Proteus mirabilis*, and *Proteus vulgaris*, while preserving the physiological intestinal microbiota [6].

Beyond its established antimicrobial role, nifuroxazide has recently garnered renewed interest owing to its demonstrated antitumor potential [5,6,7]. Mechanistically, it inhibits JAK2/TYK2-mediated STAT3 activation [7] and modulates STAT1/3/5, NF-κB, and downstream inflammatory pathways [8,9]. Preclinical studies have reported promising activity against cancer cell lines (IC_50_ = 3 µM against the U3A human fibrosarcoma-derived cell line) [8]. Furthermore, nifuroxazide has been shown to attenuate gastric mucosal inflammation by reducing HMGB1, NF-κB p65, TNF-α, and STAT3 levels, suppressing TLR4 and IL-1β expression, and lowering serum CRP [10], while also mitigating apoptosis through inhibition of caspase-3 expression [10].

The therapeutic potential of nifuroxazide extends to cardioprotection: it significantly attenuates 5-fluorouracil-induced cardiotoxicity, as evidenced by reduced serum levels of CK-MB, AST, ALP, and LDH, with histopathological examinations confirming protective effects against tissue damage [11]. These benefits are mediated through PPAR-γ signal activation and suppression of the NLRP3/STAT3 pathway [11].

Nifuroxazide also enhances the efficacy of other therapeutic agents. The combination of liraglutide and nifuroxazide has shown promise in managing septic acute lung injury by modulating the JAK2/STAT3/NF-κB signaling pathway and augmenting the ACE2/Ang1-7/MAS receptor axis [12]. In HepG2 cells, co-administration of low-dose regorafenib with nifuroxazide inhibited cell viability, proliferation, and migration, induced apoptosis, and reduced phosphorylated STAT3 expression [13]. *In* *vivo*, H22 tumor-bearing mice treated with this combination exhibited suppressed tumor growth without systemic toxicity, accompanied by changes in apoptotic proteins, enhanced tumor-infiltrating immune cells, and improved systemic immune responses [13]. Additionally, nifuroxazide-loaded RAW264.7 macrophages significantly inhibited tumor growth in a mouse hepatocellular carcinoma model and increased the M1/M2 macrophage ratio [14].

**Figure 1:**
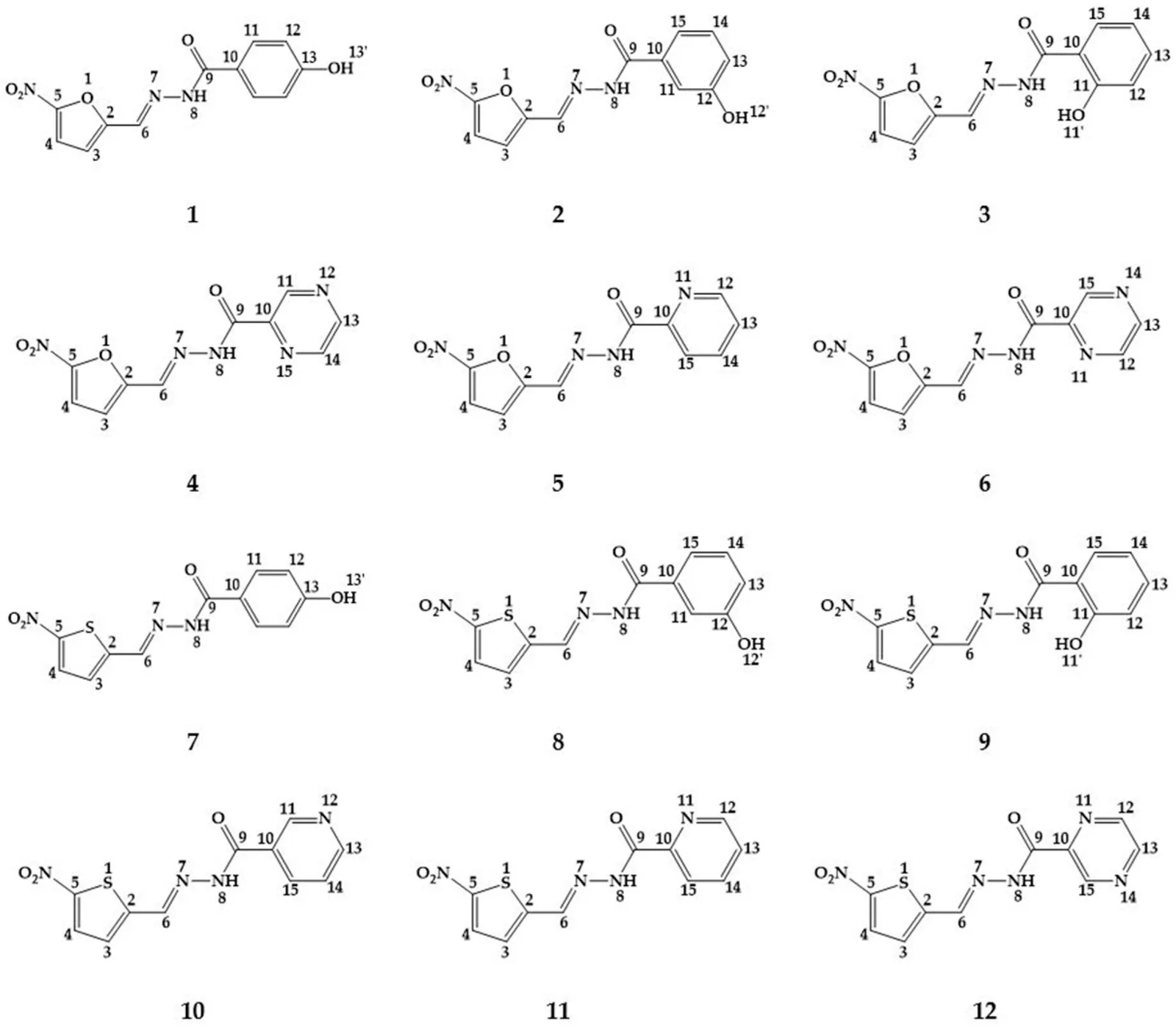
Structure of nifuroxazide (5-nitrofurfurylidene hydrazide of 4-hydroxybenzoic acid) (**1**); 5-nitrofurfurylidene hydrazide of 3-hydroxybenzoic acid (**2**); 5-nitrofurfurylidene hydrazide of 2-hydroxybenzoic acid (**3**); 5-nitrofurfurylidene hydrazide of 3-pyridinecarboxylic acid (**4**); 5-nitrofurfurylidene hydrazide of 2-pyridinecarboxylic acid (**5**); 5-nitrofurfurylidene hydrazide of pyrazinecarboxylic acid (**6**); 5-nitrothien-2-yl hydrazide of 4-hydroxybenzoic acid (**7**); 5-nitrothien-2-yl hydrazide of 3-hydroxybenzoic acid (**8**); 5-nitrothien-2-yl hydrazide of 2-hydroxybenzoic acid (**9**); 5-nitrothien-2-yl hydrazide of 3-pyridinecarboxylic acid (**10**); 5-nitrothien-2-yl hydrazide of 2-pyridinecarboxylic acid (**11**); 5-nitrothien-2-yl hydrazide of pyrazinecarboxylic acid (**12**). Atom numbering is used in NMR signal assignment (see Section 2.1 and Section 3.2).

Despite these promising attributes, nifuroxazide has notable limitations, including low systemic absorption (10 to 20% of the oral dose), with the majority of the administered drug remaining in the gut lumen and being excreted unchanged [15]. This property is both advantageous (minimizing systemic side effects) and disadvantageous, as poor absorption hinders systemic delivery, particularly if nifuroxazide is to be repositioned for non-intestinal targets. In addition, systemic pharmacokinetic data remain scarce [8]. To address these limitations, we designed a series of nifuroxazide derivatives with systematic modifications at the hydrazide and aromatic moieties, with the aim of improving bioavailability and expanding the antimicrobial spectrum. We synthesized 11 nifuroxazide derivatives and systematically evaluated their affinity toward blood serum proteins (using bovine and human albumins and *γ*-globulins as model proteins), cytotoxicity against tumor and non-tumor cell lines, and antimicrobial activity against *S. aureus*, *B. subtilis*, *E. coli*, *P. aeruginosa*, and *C. albicans*. The structures of all investigated compounds are depicted in the Figure 1.

The choice of the 5-nitrothien-2-yl moiety in compounds **7**–**12** is supported by previous findings [16], in which fourteen thiophene-substituted analogs of nifuroxazide demonstrated promising activity against multidrug-resistant *S. aureus*. It should also be noted that other chemical modifications of nifuroxazide have been explored. For example, N-benzyl substitution enhanced the antileishmanial activity of nifuroxazide [17], while introduction of a nitrile group adjacent to the carbonyl increased its cytotoxicity against both tumor (CaCo-2, MDA-MB-231 and HepG-2) and non-tumor (Wi-38, hepatocytes, Vero cells) cell lines [18]. The cytotoxicity of twelve nifuroxazide analogs containing a thiophene moiety instead of furan and varying in the *para*-substituent of the phenyl ring was evaluated against CaCo-2 cells [19].

It should be acknowledged that the compounds included in this library have been previously reported. Specifically, compounds **2** and **8** have demonstrated antileishmanial activity [20]; compounds **3** and **4** have been tested against various Gram-positive and Gram-negative bacteria as well as fungi [21,22,23]; compounds **3** and **4** have also been investigated for their ability to influence STAT3 transcriptional activity in THP-1 monocytes [24]; hydrazones **4** and **5** were tested as antimicrobial agents against *M. tuberculosis*, *S. aureus*, *V. cholerae* and *E. coli* [25]; compounds **4** and **10** as well as their complexes with divalent *d*-metal ions were found to show a wide spectrum of antibacterial activity [26] (synthesis of **10** as well as its use for cyanide detection in living organisms is also described in papers [27,28]); compounds **2**, **3**, **8**, **9** and **10** have been evaluated for cytotoxicity against Vero cells, macrophages, and *Toxoplasma gondii* [29]; hydrazone **11** shows some nonlinear optical properties [30]; and compounds **6** and **12** have been examined for antimycobacterial activity [31]. In addition, nifuroxazide analogs bearing a 4-pyridinyl moiety were studied in our previous paper [32]. A variety of biological activities were reported for **7,** including antiprotozoan and antimycobacterial effects [33]. However, these prior investigations have been fragmented, addressing isolated compounds and discrete biological endpoints without a systematic, comparative assessment across the entire series. The present study fills this gap by providing a comprehensive evaluation of the biological activity of this compound library, assessing, for the first time in a single study, their serum protein binding affinity, cytotoxicity against tumor and non-tumor cell lines, and antimicrobial activity. Given that serum protein binding critically influences the pharmacokinetics, bioavailability, and distribution of small-molecule drugs, we also assessed the affinity of all derivatives toward bovine (BSA) and human (HSA) serum albumins and *γ*-globulins (BGG, HGG) under standardized conditions (pH 6.86, *T* = 298.2 K, *p* = 0.1 MPa, *I* = 0.05 M) for the first time. The integrated biological evaluation presented herein provides a comprehensive structure-activity relationship framework that may guide future optimization of nifuroxazide-based therapeutics.

## 2. Results and Discussion

### 2.1. NMR Spectra of Synthesized ***1**–**12***

Compounds **1**–**12** were synthesized in high yield using a consistent procedure that involved refluxing the starting aldehyde (either 5-nitrofurfural or 5-nitro-2-thiophenecarboxaldehyde) with the corresponding carbohydrazide. The successful synthesis and purity of the obtained compounds were confirmed by ^1^H NMR, ^13^C NMR, IR, and mass spectrometry (Figures S1–S12). The NMR characterization data are summarized in the Table 1.

The IR spectra of the synthesized hydrazones feature an N-H stretching band of weak to medium intensity at approximately 3300 cm^−1^, characteristic of secondary amines and confirming successful condensation. By contrast, the starting carbohydrazides display two distinct bands corresponding to symmetric and asymmetric N-H stretching vibrations. Strong absorption bands attributable to the C=O and NO_2_ stretching vibrations are observed at approximately 1650 and 1350 cm^−1^, respectively, confirming retention of these functional groups in the final products.

Several notable trends emerge from the NMR data (Table 1). The position of the –NH– bridge proton signal does not depend on the nature of the five-membered ring but is influenced by the structure of the six-membered aromatic residue. Changing the position of the –OH group from *para*- to *ortho*- or *meta*- causes a slight deshielding of the imino proton (by ~0.01 ppm), whereas the introduction of heteronitrogen atoms results in a pronounced downfield shift in the –NH– resonance. This latter effect is particularly strong for β-pyridinyl derivatives (i.e., derivatives of picolinic or pyrazinic acids), indicating the formation of a relatively weak intramolecular hydrogen bond (NH···N_heter_).

The influence of intramolecular hydrogen bonding is even more evident for the –OH protons. The transition from *para*-OH- to *ortho*-OH-substituted derivatives results in a substantial downfield shift (from 10.25 to 11.53 ppm), consistent with OH···O_carbonyl_ hydrogen bond formation. This interpretation is supported by the observation that in compounds **3** and **9**, the carbonyl carbon is the most deshielded among all hydrazones. In *meta*-OH-substituted derivatives, the OH proton signals shift upfield, reflecting less efficient conjugation with the electron-withdrawing hydrazide group.

The –CH= protons of the five-membered heterocycles are largely unaffected by peripheral substitution, both in the furan series (**1**–**6**) and the thiophene series (**7**–**12**). The downfield shifts observed for the H3, C3, H4, and C4 resonances when comparing the furan (**1**–**6**) and thiophene (**7**–**12**) sets can be attributed to the greater aromatic character of thiophene relative to furan. This same factor likely accounts for the slight downfield shift in the C=O carbonyl resonances in thiophene derivatives compared with their furan counterparts.

The C2 carbon signal is the most sensitive to the presence of a heterocyclic nitrogen atom at the *ortho*- position relative to the hydrazide bridge. In contrast, the C5 carbon signals, adjacent to the nitro group, do not shift with changes in the six-membered aromatic system and move upfield by only ~1 ppm upon substitution of oxygen by sulfur in the furan ring. The C10 signal, directly attached to the hydrazide bridge, shifts similarly in both furan and thienyl derivatives, which is consistent with the large distance separating C10 from the five-membered aromatic ring.

These observations are relevant to the subsequent biochemical discussion, as they reveal key structural features of the molecules; in particular, the presence of intramolecular hydrogen bonds (most pronounced for compounds **3**, **5**, **6**, **9**, **11**, and **12**).

### 2.2. Equilibria of ***1**–**12*** Association with Blood Serum Albumins and γ-Globulins

The primary spectrofluorimetric data are presented in the Figures S13–S24. In all cases, the addition of hydrazones resulted in quenching of the intrinsic protein fluorescence, which we attribute to changes in the microenvironment of fluorescent amino acid residues upon small-molecule association. These experimental data, together with the total concentrations of proteins and hydrazones **1**–**12**, were processed using the software KEV, ver. 0.6.0 [34], according to the following stoichiometric scheme:

(1) P + L ⇌ PL,

where P denotes the protein and L denotes the hydrazone.

It should be noted that we also considered the formation of associates with alternative protein:ligand stoichiometries, namely P:L = 1:2 and 1:3. However, in all cases, increasing the stoichiometric coefficient of the ligand to 2 or 3 led to a significant increase in the standard deviation of the calculated equilibrium constant, a decrease in *R_adj_*_._^2^, and a visible worsening of the fitting quality. Examples of calculations using different stoichiometric schemes are provided in the Figure S25 for the BSA + **7** system. Accordingly, the most probable stoichiometric composition of the associates formed was concluded to be 1:1 (PL), and the corresponding conditional binding constants are reported in the Table 2.

**Figure 2:**
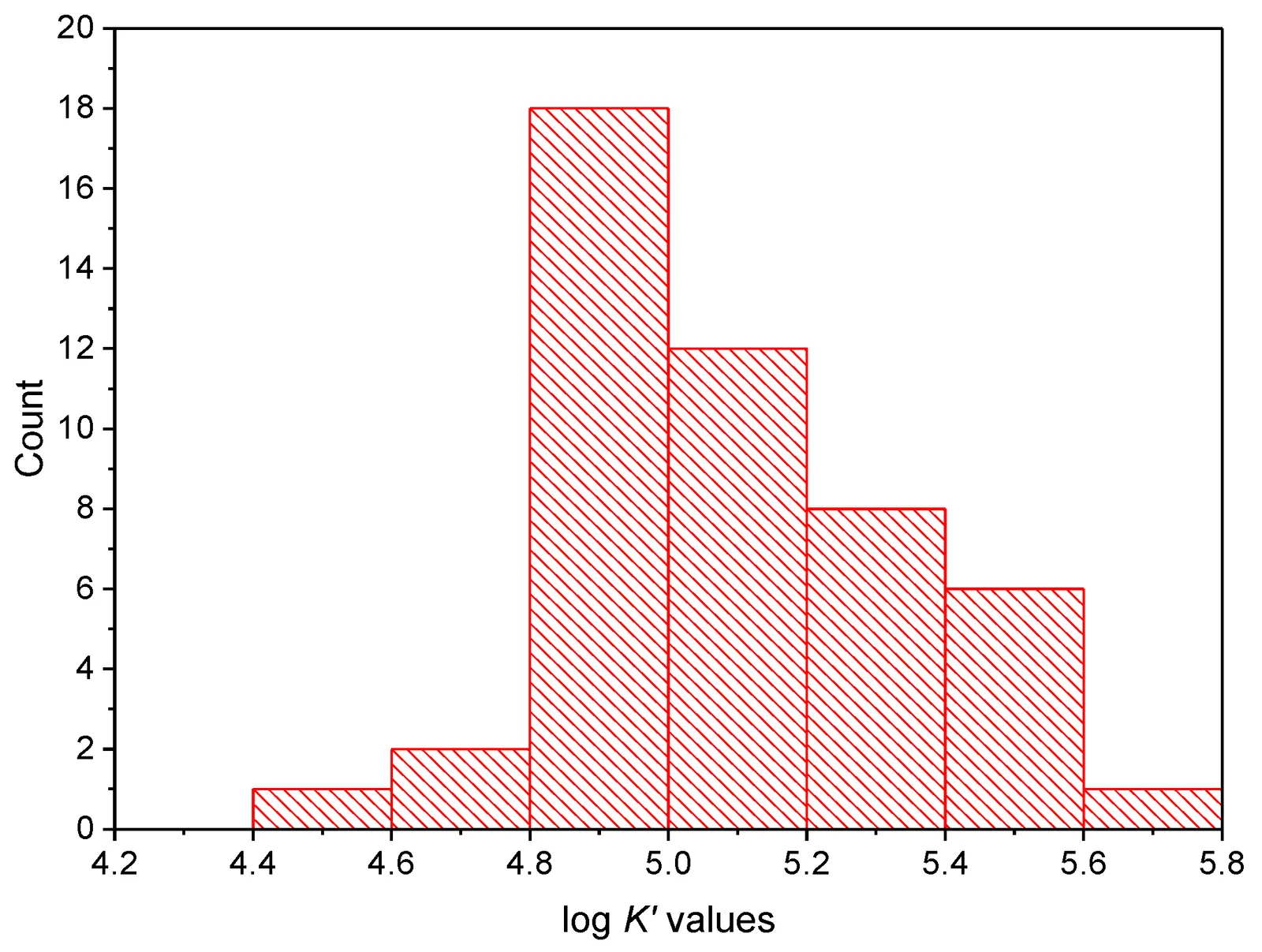
Frequency distribution of log *K*′ values determined for various proteins and hydrazones.

As the data in the Table 2 suggest, the conditional binding constants, regardless of ligand and protein, vary within the relatively narrow range of 4.4 to 5.6 log units. This variation is more likely attributable to experimental standard deviations and the complex nature of ligand–protein interactions than to any specific selectivity in the interactions. A distribution test (Figure 2) shows that the data in the Table 2 can be described by a histogram that does not significantly differ from a normal distribution (*p* = 0.066). Thus, the null hypothesis (that the values are normally distributed) cannot be rejected.

Taken together, the data in the Table 2 should be interpreted as indicating that all ligands exhibit similar affinity toward all proteins tested, with a mean value of log *K*′ = 5.0 ± 0.3. This value indicates relatively strong binding of the hydrazones to albumins, which are characterized by relatively high blood serum concentrations (on the order of 0.6 mM [35,36]). Such strong affinity would ensure strong drug-to-protein binding (90–100%), a factor that would be relevant if nifuroxazide and its analogs were to be introduced into the bloodstream [37]. These experimental results may therefore prove useful in the context of potential nifuroxazide repurposing, which would require, for example, intravenous administration, and contribute to the currently limited understanding of nifuroxazide and its analogs’ pharmacodynamics and pharmacokinetics [8].

### 2.3. Cytotoxicity of ***1**–**12***

The cytotoxicity of hydrazones **1**–**12** was evaluated using an MTT assay against human colorectal carcinoma (HCT116) and immortalized human embryonic kidney (HEK293) cell lines. The results are summarized in the Table 3, and the primary dose–response data are presented in the Figure S26.

While the present study was not designed to elucidate the precise molecular mechanism of action, the observed cytotoxicity trends can be interpreted in the context of the known mechanism of nifuroxazide. Nelson et al. [38] demonstrated that nifuroxazide inhibits phosphorylation of JAK2 and TYK2, upstream activator kinases of STAT3, thereby reducing STAT3 activity and leading to downregulation of the STAT3 target gene *Mcl-1*, which was associated with decreased cell survival. Da Costa et al. [39] further elucidated the binding mode through molecular docking simulations, proposing that nifuroxazide acts as a Type II kinase inhibitor, occupying both the ATP-binding site and the adjacent allosteric pocket of JAK2, with key interactions involving hydrogen bonds to Glu930, Leu932, Asp994, and His974. With this mechanistic framework, the SAR trends observed in our cytotoxicity data can be rationalized in the following way: the 2-pyridinyl derivatives **5** and **11**, forming intramolecular NH···N_heter_ hydrogen bonds (Section 2.1), are preorganized for optimal JAK2 binding, consistent with their high potency (IC_50_ = 4.9 and 10.4 µM) and selectivity (SI = 8.8 and > 4.8, respectively; these compounds were significantly more toxic to tumor cells than to non-tumor cells, *p* < 0.001 after Bonferroni–Dunn correction for multiple comparisons). In contrast, the *ortho*-hydroxy derivatives **3** and **9** form strong intramolecular OH···O_carbonyl_ hydrogen bonds that may lock the molecule in a conformation incompatible with the JAK2 binding site, reducing cytotoxicity (IC_50_ = 9.5 and 18.8 µM). The dramatic loss of activity for the pyrazinyl derivatives **6** and **12** (IC_50_ > 50 µM for **6**) likely reflects the electron-withdrawing effect of the additional ring nitrogen, which may weaken key hydrogen-bonding interactions with JAK2 residues. Although in present study we did not compare direct effect of tested compounds on STAT3, it may be hypothesized that reduced cytotoxicity of thiophene derivatives against HEK293 cells (e.g., **7**, **9**, **11** with IC_50_ > 50 µM) and the superior selectivity of **5** and **11** are consistent with the established mechanism, as nifuroxazide and its analogs are selectively toxic to cells with constitutive STAT3 activation while sparing normal cells [38].

### 2.4. Antimicrobial Action of ***1**–**12***

The diameters of the bacterial and fungal growth inhibition zones (in mm) are presented in the Table 4. Representative examples of the primary experimental data (Petri dishes) are shown in the Figures S27–S29.

The antimicrobial data reveal SAR patterns that are distinct from those observed for cytotoxicity, indicating that the structural requirements for antibacterial activity differ from those for antitumor activity.

The antimicrobial action of nitrofuran derivatives, including nifuroxazide, is believed to be primarily mediated by the enzymatic reduction of the 5-nitro group by bacterial nitroreductases [40]. This reductive activation generates reactive oxygen species (ROS) and electrophilic intermediates that damage bacterial DNA, proteins, and membranes, ultimately leading to bacterial cell death. The 5-nitro group is therefore a pharmacophoric requirement for antibacterial activity, and its presence in all compounds **1**–**12** explains the observed activity against Gram-positive strains.

The superior activity of the furan series (**1**–**6**) compared to the thiophene series (**7**–**12**) may reflect differences in the reduction potential of the nitro group. The more electron-rich furan ring may facilitate nitroreduction by bacterial enzymes, whereas the more aromatic thiophene ring may slightly increase the reduction potential, making nitroreduction less favorable. This interpretation is supported by the electrochemical data discussed for related nitrofuran and nitrothiophene derivatives [40]; however, Sidorov et al. [40] indicate the controversies in the literature and lack of any meaningful correlation between the electronegativity of the substituents introduced and the activity of the substituted nifuroxazide analogs. Alternatively, the reduced activity of thiophene derivatives may arise from differences in membrane permeability or efflux susceptibility, though these factors were not directly assessed in our study.

Among the furan derivatives, compounds **2** and **4** (bearing *meta*-substituents) exhibited the highest activity. We propose that the *meta*-substitution pattern optimizes the balance between hydrophilicity (for diffusion through agar and aqueous media) and hydrophobicity (for penetration through the bacterial membranes). The *para*-hydroxy derivative **1** (nifuroxazide) is more hydrophilic due to the *para*-OH group engaged in conjugation with the hydrazide bridge, which may reduce its membrane permeability. The *ortho*-OH derivatives (**3**, **9**) are conformationally constrained by intramolecular H-bonds, which may hinder their interaction with bacterial nitroreductases or their access to intracellular targets.

The limited activity against Gram-negative *E. coli* and inactivity against *P. aeruginosa* are consistent with the known spectrum of nifuroxazide [6,40]. This selectivity likely reflects differences in outer membrane permeability and the presence of efflux pumps. *P. aeruginosa* is notoriously difficult to target due to its highly impermeable outer membrane and multidrug efflux systems, which may effectively exclude these hydrazones from the periplasmic space. The lack of activity against *C. albicans* is consistent with the previous findings [23,40].

## 3. Materials and Methods

### 3.1. Chemicals

5-Nitrofurfural, 5-nitrothiophene-2-carboxaldehyde, and the hydrazides of 3-hydroxybenzoic acid, 2-hydroxybenzoic acid, 3-pyridinecarboxylic acid, 2-pyridinecarboxylic acid, and pyrazinecarboxylic acid were purchased from Shanghai Macklin Biochemical Technology Co., Ltd. (Shanghai, China) with a claimed purity of >99% and were used for synthesis without further purification. Phosphate buffer (pH 6.86) was prepared from a standard solution purchased from Uralkhiminvest (Ufa, Russia), supplied in sealed glass ampoules. The buffer contained KH_2_PO_4_ and Na_2_HPO_4_ at a final concentration of 0.005 mol kg^−1^ each, as well as 0.03 M NaCl (Reakhim, Moscow, Russia), yielding a final ionic strength of 0.05 M. All solutions were prepared using double-distilled, deaerated water (pH 6.4, specific conductivity *κ* = 2.1 μS cm^−1^).

The concentration of each protein solution, typically in the range of 5 to 15 μM, was determined spectrophotometrically prior to each set of experiments. Molar absorption coefficients of 43,824 M^−1^ cm^−1^ for BSA [41] and 35,700 M^−1^ cm^−1^ for HSA [42] were used for this purpose. Protein stock solutions were prepared in phosphate buffer (pH 6.86) containing 0.05 M NaCl to maintain constant ionic strength and were stored at 4 °C for no more than three days.

DMSO-d_6_ (isotopic purity 99.8 atom %), used for NMR measurements, was also supplied by Shanghai Macklin Biochemical Technology Co., Ltd.

### 3.2. Synthesis of ***1**–**12***

**Figure 3:**
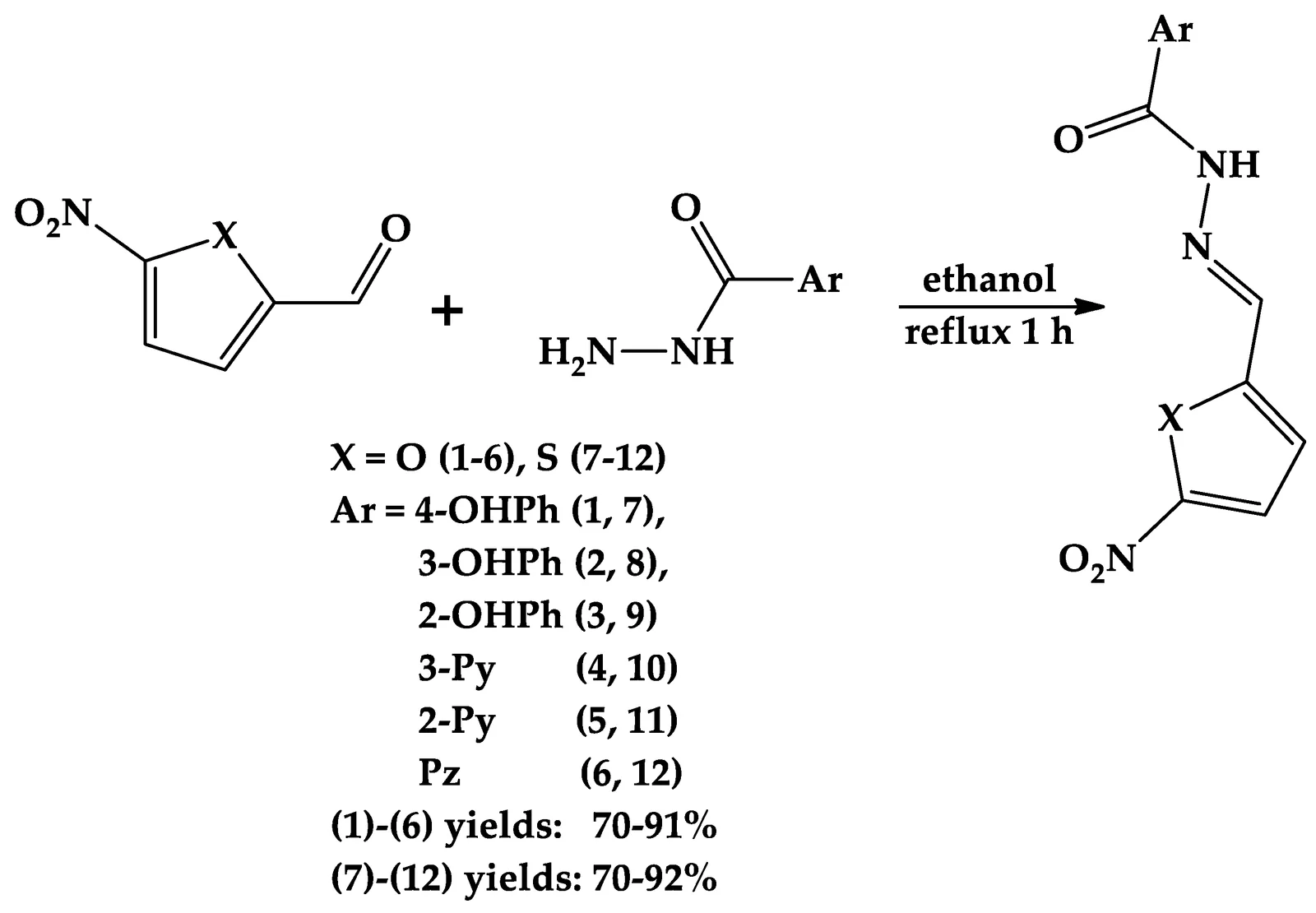
Scheme of synthesis of hydrazones **1**–**12**.

Hydrazones **1**–**12** were synthesized according to the following general procedure (Figure 3).

A 2 mmol portion of 5-nitrofurfural or 5-nitrothiophene-2-carboxaldehyde was dissolved in 50 mL of ethanol in a round-bottomed flask with gentle heating (~50 °C). A solution of the corresponding carbohydrazide (2 mmol) in 25 mL of ethanol was then added dropwise, and the mixture was heated at reflux for 1 h with stirring. The progress of the reaction was monitored by TLC. After cooling to room temperature, the precipitated product was collected by vacuum filtration, washed with cold ethanol, and dried to constant weight at 80 °C. For hydrazones **5** and **8**, recrystallization from ethanol was necessary to obtain pure products.

The compounds were characterized using IR, ^1^H and ^13^C NMR spectroscopy as well as mass spectrometry (Figures S1–S24). The following notation is used while describing IR spectra: s stands for a strong band, while w denotes a weak band. The following notation is used while describing ^1^H NMR spectra: s for singlet, d for doublet, t for triplet, dd for doublet of doublets, td for triplet of doublets.

**4-Hydroxy-N′-((5-nitrofuran-2-yl)methylene)benzohydrazide, 1.** Yellow powder, m.p. 282 °C, yield 70%. Anal. Calcd. for C_12_H_9_N_3_O_5_ (275.22): C, 52.37; H, 3.30; N, 15.27; O 29.07. Found: C, 52.59; H, 3.13; N, 15.20; O 29.09. IR (KBr), cm^−1^: 3367s, 3248s, 3130s, 3033w, 1674s, 1606s, 1583s, 1554s, 1508s, 1467s, 1444s, 1406s, 1363s, 1259s, 1188s, 1153s, 1116s, 1026s, 983s, 964s, 933s, 904s, 852s, 798s, 760s, 721s, 625s, 548s. ^1^H NMR, *δ*, ppm (500 MHz, DMSO-*d*_6_): 12.06s (1H, NH), 10.25s (1H, OH), 8.38s (1H, H_6_), 7.82d (^3^*J* = 8.5 Hz, 2H, H_11_), 7.80s (1H, H_4_), 7.25d (^3^*J* = 3.9 Hz, 1H, H_3_), 6.88d (^3^*J* = 8.5 Hz, 2H, H_12_). ^13^C NMR, *δ*, ppm (125 MHz, DMSO-*d*_6_): 161.6 (C_9_), 152.5 (C_5_, C_10_), 152.3 (C_2_), 134.9 (C_6_), 130.4 (C_11_), 123.6 (C_12_), 115.6 (C_4_), 115.4 (C_10_), 115.2 (C_3_). *m*/*z* = 276.28 [**1** + H^+^], theoretical *m*/*z* = 276.22 [**1** + H^+^]; *m*/*z* = 298.30 [**1** + Na^+^], theoretical *m*/*z* = 298.26 [**1** + Na^+^].

**3-Hydroxy-N′-((5-nitrofuran-2-yl)methylene)benzohydrazide, 2.** Light-brown powder, m.p. 279 °C, yield 73%. Anal. Calcd. for C_12_H_9_N_3_O_5_ (275.22): C, 52.37; H, 3.30; N, 15.27; O 29.07. Found: C, 52.44; H, 3.21; N, 15.14; O 29.21. IR (KBr), cm^−1^: 3313s, 3211s, 3020w, 1658s, 1581s, 1554s, 1469s, 1354s, 1292s, 1246s, 1132s, 1082s, 1024s, 987s, 937s, 889s, 848s, 808s, 744s, 683s, 569s. ^1^H NMR, *δ*, ppm (500 MHz, DMSO-*d*_6_): 12.16s (1H, H_8_), 9.82s (1H, H_12′_), 8.41s (1H, H_6_), 7.81d (^3^*J* = 3.9 Hz, 1H, H_4_), 7.35s (1H, H_15_), 7.34s (1H, H_11_), 7.30s (1H, H_14_), 7.27d (^3^*J* = 3.9 Hz, 1H, H_3_), 7.01dd (^3^*J* = 7.3 Hz, ^4^*J* = 4.0 Hz, 1H, H_13_). ^13^C NMR, *δ*, ppm (125 MHz, DMSO-*d*_6_): 163.9 (C_9_), 158.0 (C_12_), 152.4 (C_5_), 152.3 (C_2_), 135.9 (C_10_), 134.6 (C_6_), 130.2 (C_14_), 119.6 (C_15_), 118.7 (C_13_), 115.7 (C_4_), 115.2 (C_11_), 115.0 (C_3_). *m*/*z* = 276.24 [**2** + H^+^], theoretical *m*/*z* = 276.22 [**2** + H^+^]; *m*/*z* = 298.23 [**2** + Na^+^], theoretical *m*/*z* = 298.26 [**2** + Na^+^].

**2-Hydroxy-N′-((5-nitrofuran-2-yl)methylene)benzohydrazide, 3.** Brown powder, m.p. 258 °C, yield 84%. Anal. Calcd. for C_12_H_9_N_3_O_5_ (275.22): C, 52.37; H, 3.30; N, 15.27; O 29.07. Found: C, 52.56; H, 3.26; N, 15.19; O 29.00. IR (KBr), cm^−1^: 3228s, 3126s, 3076w, 1631s, 1583s, 1535s, 1512s, 1467s, 1388s, 1346s, 1288s, 1248s, 1230s, 1194s, 1134s, 1018s, 968s, 939s, 902s, 806s, 754s, 661s. ^1^H NMR, *δ*, ppm (500 MHz, DMSO-*d*_6_): 12.14s (1H, H_8_), 11.53s (1H, H_11′_), 8.43s (1H, H_6_), 7.83d (^3^*J* = 7.2 Hz, 1H, H_15_), 7.82d (^3^*J* = 3.7 Hz, 1H, H_4_), 7.46t (^3^*J* = 7.2 Hz, 1H, H_13_), 7.29d (^3^*J* = 3.7 Hz, 1H, H_3_), 7.00d (^3^*J* = 8.6 Hz, 1H, H_14_), 6.97d (^3^*J* = 7.6 Hz, 1H, H_12_). ^13^C NMR, *δ*, ppm (125 MHz, DMSO-*d*_6_): 165.1 (C_9_), 158.8 (C_11_), 152.5 (C_5_), 152.1 (C_2_), 136.5 (C_6_), 134.5 (C_13_), 129.6 (C_15_), 119.6 (C_14_), 117.6 (C_12_), 117.0 (C_10_), 116.1 (C_4_), 115.1 (C_3_). *m*/*z* = 276.37 [**3** + H^+^], theoretical *m*/*z* = 276.22 [**3** + H^+^]; *m*/*z* = 298.39 [**3** + Na^+^], theoretical *m*/*z* = 298.26 [**3** + Na^+^].

**N′-((5-nitrofuran-2-yl)methylene)pyridine-3-carbohydrazide, 4.** Light-brown powder, m.p. 240 °C, yield 91%. Anal. Calcd. for C_11_H_8_N_4_O_4_ (260.21): C, 50.77; H, 3.10; N, 21.53; O, 24.60. Found: C, 50.91; H, 2.94; N, 21.47; O, 24.68. IR (KBr), cm^−1^: 3277s, 3149s, 3070w, 1685s, 1535s, 1504s, 1467s, 1354s, 1255s, 1145s, 1070s, 1024s, 964s, 933s, 814s, 740s, 698s, 621s. ^1^H NMR, *δ*, ppm (500 MHz, DMSO-*d*_6_): 12.41s (1H, H_8_), 9.08s (1H, H_11_), 8.80d (^3^*J* = 4.2 Hz, 1H, H_13_), 8.39s (1H, H_6_), 8.27d (^3^*J* = 7.8 Hz, 1H, H_15_), 7.82d (^3^*J* = 4.2 Hz, 1H, H_4_), 7.60dd (^3^*J* = 7.8, 4.2 Hz, 1H, H_14_), 7.32d (^3^*J* = 3.6 Hz, 1H, H_3_). ^13^C NMR, *δ*, ppm (125 MHz, DMSO-*d*_6_): 162.5 (C_9_), 153.2 (C_13_), 152.5 (C_5_), 151.9 (C_2_), 149.2 (C_11_), 136.6 (C_6_), 136.1 (C_15_), 129.1 (C_10_), 124.2 (C_14_), 116.3 (C_4_), 115.1 (C_3_). *m*/*z* = 261.24 [**4** + H^+^], theoretical *m*/*z* = 261.231 [**4** + H^+^]; *m*/*z* = 283.24 [**4** + Na^+^], theoretical *m*/*z* = 283.25 [**4** + Na^+^].

**N′-((5-nitrofuran-2-yl)methylene)pyridine-2-carbohydrazide, 5.** Yellowish-brown powder, m.p. 254 °C (decomposes), yield 87%. Anal. Calcd. for C_11_H_8_N_4_O_4_ (260.21): C, 50.77; H, 3.10; N, 21.53; O, 24.60. Found: C, 50.93; H, 3.00; N, 21.34; O, 24.74. IR (KBr), cm^−1^: 3253s, 3144s, 3070w, 1657s, 1593s, 1556s, 1510s, 1471s, 1421s, 1392s, 1348s, 1317s, 1280s, 1250s, 1142s, 1118s, 1074s, 1012s, 962s, 904s, 810s, 737s, 702s, 619s. ^1^H NMR, *δ*, ppm (500 MHz, DMSO-*d*_6_): 12.71s (1H, H_8_), 8.75d (^3^*J* = 4.5 Hz, 1H, H_12_), 8.63s (1H, H_6_), 8.16d (^3^*J* = 7.8 Hz, 1H, H_15_), 8.08td (^3^*J* = 7.8 Hz, ^4^*J* = 1.4 Hz, 1H, H_14_), 7.81d (^3^*J* = 4.5 Hz, 1H, H_4_), 7.71dd (^3^*J* = 7.8 Hz, 1H, H_13_), 7.28d (^3^*J* = 4.5 Hz, 1H, H_3_). ^13^C NMR, *δ*, ppm (125 MHz, DMSO-*d*_6_): 161.3 (C_9_), 152.4 (C_5_), 152.3 (C_10_), 149.5 (C_2_), 149.1 (C_12_), 138.6 (C_6_), 137.3 (C_14_), 127.9 (C_13_), 123.5 (C_15_), 115.6 (C_4_), 115.1 (C_3_). *m*/*z* = 261.21 [**5** + H^+^], theoretical *m*/*z* = 261.06 [**5** + H^+^]; *m*/*z* = 283.20 [**5** + Na^+^], theoretical *m*/*z* = 283.04 [**5** + Na^+^].

**N′-((5-nitrofuran-2-yl)methylene)pyrazine-2-carbohydrazide, 6.** Light-brown powder, m.p. 282 °C, yield 80%. Anal. Calcd. for C_10_H_7_N_5_O_4_ (261.19): C, 45.98; H, 2.70; N, 26.81; O, 24.50. Found: C, 46.11; H, 2.64; N, 26.68; O, 24.57. IR (KBr), cm^−1^: 3267s, 3151s, 3012s, 1682s, 1566s, 1537s, 1510s, 1475s, 1402s, 1356s, 1263s, 1155s, 1068s, 1020s, 964s, 941s, 914s, 868s, 810s, 765s, 737s, 640s, 438s. ^1^H NMR, *δ*, ppm (500 MHz, DMSO-*d*_6_): 12.79s (1H, H_8_), 9.30d (^4^*J* = 1.4 Hz, 1H, H_15_), 8.97d (^3^*J* = 2.5 Hz, 1H, H_12_), 8.82dd (^3^*J* = 2.5 Hz, ^4^*J* = 1.4 Hz, 1H, H_13_), 8.62s (1H, H_6_), 7.81d (^3^*J* = 3.9 Hz, 1H, H_4_), 7.31d (^3^*J* = 3.9 Hz, 1H, H_3_). ^13^C NMR, *δ*, ppm (125 MHz, DMSO-*d*_6_): 160.4 (C_9_), 152.5 (C_5_), 152.0 (C_2_), 148.7 (C_13_), 144.8 (C_15_), 144.6 (C_10_), 143.8 (C_12_), 137.9 (C_6_), 116.1 (C_4_), 115.1 (C_5_). *m*/*z* = 262.20 [**6** + H^+^], theoretical *m*/*z* = 262.19 [**6** + H^+^]; *m*/*z* = 284.21 [**6** + Na^+^], theoretical *m*/*z* = 284.23 [**6** + Na^+^].

**4-Hydroxy-N′-((5-nitrothiophen-2-yl)methylene)benzohydrazide, 7.** Yellow powder, m.p. 254 °C (decomposes), yield 72%. Anal. Calcd. for C_12_H_9_N_3_O_4_S (291.28): C, 49.48; H, 3.11; N, 14.43; S, 11.01; O, 21.97. Found: C, 49.63; H, 3.04; N, 14.35; S, 10.93; O, 22.05. IR (KBr), cm^−1^: 3367s, 3315s, 3109w, 1668s, 1604s, 1577s, 1502s, 1431s, 1336s, 1261s, 1182s, 1111s, 1030s, 937s, 848s, 812s, 690s, 619s, 522s. ^1^H NMR, *δ*, ppm (500 MHz, DMSO-*d*_6_): 12.05s (1H, NH), 10.25s (1H, OH), 8.66s (1H, H_6_), 8.13d (^3^*J* = 4.2 Hz, 1H, H_4_), 7.81d (^3^*J* = 8.2 Hz, 1H, H_11_), 7.55d (^3^*J* = 4.2 Hz, 1H, H_3_), 6.88d (^3^*J* = 8.2 Hz, 1H, H_12_). ^13^C NMR, *δ*, ppm (125 MHz, DMSO-*d*_6_): 163.4 (C_9_), 161.5 (C_13_), 151.0 (C_5_), 147.6 (C_2_), 140.6 (C_6_), 131.0 (C_11_), 130.4 (C_4_), 129.7 (C_3_), 123.7 (C_12_), 115.6 (C_10_). *m*/*z* = 292.19 [**7** + H^+^], theoretical *m*/*z* = 292.28 [**7** + H^+^]; *m*/*z* = 314.25 [**7** + Na^+^], theoretical *m*/*z* = 314.26 [**7** + Na^+^].

**3-Hydroxy-N′-((5-nitrothiophen-2-yl)methylene)benzohydrazide, 8.** Yellow powder, m.p. 270 °C, yield 71%. Anal. Calcd. for C_12_H_9_N_3_O_4_S (291.28): C, 49.48; H, 3.11; N, 14.43; S, 11.01; O, 21.97. Found: C, 49.66; H, 3.07; N, 14.27; S, 10.88; O, 22.12. IR (KBr), cm^−1^: 3338s, 3226s, 3109s, 1653s, 1610s, 1577s, 1550s, 1521s, 1489s, 1431s, 1338s, 1284s, 1234s, 1130s, 1084s, 1030s, 960s, 931s, 868s, 804s, 738s, 681s, 511s. ^1^H NMR, *δ*, ppm (500 MHz, DMSO-*d*_6_): 12.18s (1H, H_8_), 9.85s (1H, H_12′_), 8.67s (1H, H_6_), 8.14d (^3^*J* = 4.1 Hz, 1H, H_4_), 7.58d (^3^*J* = 4.1 Hz, 1H, H_3_), 7.34s (1H, H_11_), 7.33s (1H, H_15_), 7.29s (1H, H_14_), 7.01s (1H, H_13_). ^13^C NMR, *δ*, ppm (125 MHz, DMSO-*d*_6_): 163.8 (C_9_), 157.9 (C_12_), 151.3 (C_5_), 147.3 (C_2_), 141.5 (C_6_), 134.7 (C_10_), 131.0 (C_14_), 130.1 (C_4_), 129.4 (C_3_), 119.6 (C_15_), 118.9 (C_13_), 115.0 (C_11_). *m*/*z* = 292.24 [**8** + H^+^], theoretical *m*/*z* = 292.28 [**8** + H^+^]; *m*/*z* = 314.25 [**8** + Na^+^], theoretical *m*/*z* = 314.26 [**8** + Na^+^].

**2-Hydroxy-N′-((5-nitrothiophen-2-yl)methylene)benzohydrazide, 9.** Yellow powder, m.p. 250 °C (decomposes), yield 88%. Anal. Calcd. for C_12_H_9_N_3_O_4_S (291.28): C, 49.48; H, 3.11; N, 14.43; S, 11.01; O, 21.97. Found: C, 49.53; H, 3.12; N, 14.40; S, 10.95; O, 22.01. IR (KBr), cm^−1^: 3240s, 3030s, 1635s, 1604s, 1550s, 1494s, 1450s, 1433s, 1369s, 1338s, 1305s, 1221s, 1151s, 1080s, 1032s, 912s, 818s, 754s, 729s, 648s. ^1^H NMR, *δ*, ppm (500 MHz, DMSO-*d*_6_): 12.10s (1H, H_8_), 11.53s (1H, H_11′_), 8.68s (1H, H_6_), 8.13d (^3^*J* = 4.1 Hz, 1H, H_4_), 7.83d (^3^*J* = 7.8 Hz, 1H, H_15_), 7.58d (^3^*J* = 4.1 Hz, 1H, H_3_), 7.45t (^3^*J* = 7.8 Hz, 1H, H_13_), 6.99d (^3^*J* = 8.8 Hz, 1H, H_14_), 6.96d (^3^*J* = 7.8 Hz, 1H, H_12_). ^13^C NMR, *δ*, ppm (125 MHz, DMSO-*d*_6_): 165.0 (C_9_), 158.8 (C_11_), 151.5 (C_5_), 146.9 (C_2_), 142.2 (C_6_), 134.4 (C_13_), 131.0 (C_15_), 130.4 (C_4_), 129.5 (C_3_), 119.6 (C_14_), 117.6 (C_12_), 117.0 (C_10_). *m*/*z* = 292.21 [**9** + H^+^], theoretical *m*/*z* = 292.28 [**9** + H^+^]; *m*/*z* = 314.23 [**9** + Na^+^], theoretical *m*/*z* = 314.26 [**9** + Na^+^].

**N′-((5-nitrothiophen-2-yl)methylene)pyridine-3-carbohydrazide, 10.** Yellow powder, m.p. 200 °C, yield 92%. Anal. Calcd. for C_11_H_8_N_4_O_3_S (276.27): C, 47.82; H, 2.92; N, 20.28; S, 11.61; O, 17.37. Found: C, 47.89; H, 2.94; N, 20.22; S, 11.54; O, 17.41. IR (KBr), cm^−1^: 3063s, 1670s, 1587s, 1496s, 1427s, 1385s, 1332s, 1304s, 1219s, 1165s, 1026s, 926s, 816s, 731s. ^1^H NMR, *δ*, ppm (500 MHz, DMSO-*d*_6_): 12.38s (1H, H_8_), 9.07s (1H, H_11_), 8.79d (^3^*J* = 4.2 Hz, 1H, H_13_), 8.68s (1H, H_6_), 8.26d (^3^*J* = 7.6 Hz, 1H, H_15_), 8.14d (^3^*J* = 7.6 Hz, 1H, H_4_), 7.62d (^3^*J* = 4.2 Hz, 1H, H_3_), 7.59dd (^3^*J* = 7.6, 5.2 Hz, 1H, H_14_). ^13^C NMR, *δ*, ppm (125 MHz, DMSO-*d*_6_): 162.5 (C_9_), 153.1 (C_13_), 151.5 (C_5_), 149.1 (C_2_), 146.8 (C_11_), 142.3 (C_6_), 136.0 (C_15_), 130.5 (C_3_), 131.0 (C_4_), 129.2 (C_10_), 124.1 (C_14_). *m*/*z* = 277.22 [**10** + H^+^], theoretical *m*/*z* = 277.27 [**10** + H^+^].

**N′-((5-nitrothiophen-2-yl)methylene)pyridine-2-carbohydrazide, 11.** Yellow powder, m.p. 260 °C (decomposes), yield 72%. Anal. Calcd. for C_11_H_8_N_4_O_3_S (276.27): C, 47.82; H, 2.92; N, 20.28; S, 11.61; O, 17.37. Found: C, 47.98; H, 2.86; N, 20.16; S, 11.48; O, 17.52. IR (KBr), cm^−1^: 3269s, 3097s, 3063s, 1689s, 1583s, 1521s, 1431s, 1340s, 1278s, 1230s, 1151s, 1078s, 1037s, 995s, 912s, 810s, 737s, 629s. ^1^H NMR, *δ*, ppm (500 MHz, DMSO-*d*_6_): 12.65s (1H, H_8_), 8.86s (1H, H_6_), 8.74d (^3^*J* = 4.5 Hz, 1H, H_12_), 8.15d (^3^*J* = 4.5 Hz, 1H, H_4_), 8.14s (1H, H_15_), 8.08td (^3^*J* = 7.7 Hz, ^4^*J* = 1.3 Hz, 1H, H_14_), 7.71dd (^3^*J* = 7.7, 3.7 Hz, 1H, H_13_), 7.55d (^3^*J* = 4.5 Hz, 1H, H_3_). ^13^C NMR, *δ*, ppm (125 MHz, DMSO-*d*_6_): 161.2 (C_9_), 151.4 (C_5_), 149.5 (C_2_), 149.1 (C_12_), 147.2 (C_10_), 147.2 (C_6_), 138.6 (C_14_), 131.0 (C_4_), 130.2 (C_3_), 127.8 (C_13_), 123.5 (C_15_). *m*/*z* = 277.22 [**11** + H^+^], theoretical *m*/*z* = 277.27 [**11** + H^+^].

**N′-((5-nitrothiophen-2-yl)methylene)pyrazine-2-carbohydrazide, 12.** Yellow-orange powder, m.p. 270 °C (decomposes), yield 70%. Anal. Calcd. for C_10_H_7_N_5_O_3_S (277.26): C, 43.32; H, 2.54; N, 25.26; S, 11.56; O, 17.31. Found: C, 43.51; H, 2.48; N, 25.10; S, 11.43; O, 17.49. IR (KBr), cm^−1^: 3273s, 3097s, 3061s, 1682s, 1585s, 1523s, 1437s, 1404s, 1340s, 1269s, 1223s, 1151s, 1070s, 1041s, 1016s, 943s, 916s, 868s, 839s, 814s, 767s, 729s, 629s. ^1^H NMR, *δ*, ppm (500 MHz, DMSO-*d*_6_): 12.75s (1H, H_8_), 9.28s (1H, H_15_), 8.96d (^4^*J* = 2.2 Hz, 1H, H_12_), 8.85s (1H, H_6_), 8.82s (1H, H_13_), 8.14d (^3^*J* = 4.3 Hz, 1H, H_4_), 7.58d (^3^*J* = 4.3 Hz, 1H, H_3_). ^13^C NMR, *δ*, ppm (125 MHz, DMSO-*d*_6_): 160.4 (C_9_), 151.6 (C_5_), 148.6 (C_2_), 146.8 (C_10_), 144.8 (C_13_), 144.7 (C_12_), 143.9 (C_15_), 143.4 (C_6_), 130.9 (C_4_), 130.5 (C_3_). *m*/*z* = 278.20 [**12** + H^+^], theoretical *m*/*z* = 278.26 [**12** + H^+^]; *m*/*z* = 300.20 [**12** + Na^+^], theoretical *m*/*z* = 300.24 [**12** + Na^+^].

### 3.3. Spectral Measurements

Concentration control of the studied proteins was performed using a UV-1800 spectrophotometer (Shimadzu, Canby, OR, USA) over the wavelength range of 200 to 400 nm, with absorbance measured from 0 to 2 log units. The molar extinction coefficients of hydrazones **1**–**12**, required for correcting inner filter effects, were also determined over the wavelength range of 200 to 500 nm using the same spectrophotometer via the calibration plot method.

Protein binding experiments (spectrofluorimetric titrations) were performed using a CM2203 spectrofluorometer (Solar, Minsk, Belarus). An aliquot of 2.5 mL of buffered aqueous solution (pH 6.86, *I* = 0.05 M) containing BSA, HSA, BGG, or HGG at concentrations ranging from 5 to 15 µM was titrated with solutions of hydrazones **1**–**12** in dimethyl sulfoxide at a concentration of 5.0 ± 0.5 mM. In total, ten additions of 1 µL each were made, resulting in a final DMSO mole fraction of less than 0.001 to avoid any DMSO-related effects on protein properties. Emission spectra were recorded over the wavelength range of 290 to 400 nm with an excitation wavelength (*λ_ex_*) of 280 nm. Both excitation and emission slit widths were set to 5 nm, and quartz cuvettes with a 1.00 cm optical path length were used. All experiments were performed in triplicate.

The inner filter effect arising from increasing hydrazone concentrations was corrected using the following equation [43,44]:

(2) *F_corr_* = *F_obs_* × 10^0.5(*A_ex_*+*A_em_*)^

where *F_corr_* and *F_obs_* are the corrected and observed fluorescence intensities, respectively, and *A_ex_* and *A_em_* are the absorbance values at the excitation and emission wavelengths, respectively.

The samples for IR spectroscopy were prepared via fine dispersion of hydrazones **1**–**12** with KBr used as a matrix. Infrared spectra of all compounds were recorded using an Avatar 360 FTIR spectrometer (Thermo Nicolet, Sugarland, TX, USA). The samples were prepared as KBr pellets. Spectra were collected over the wavenumber range of 4000 to 400 cm^−1^ with a spectral resolution of 2 cm^−1^. The positions of the absorption bands were determined with an accuracy of ±2 cm^−1^.

^1^H and ^13^C NMR spectra were recorded using a Bruker Avance III 500 NMR spectrometer (Bruker, Mannheim, Germany) operating at ^1^H and ^13^C frequencies of 500.17 and 125.77 MHz, respectively. The temperature was maintained at 298.2 ± 0.1 K using a Bruker variable temperature unit (BVT-2000, Bruker, Mannheim, Germany); samples were not spun during data acquisition. Chemical shifts were referenced to an external standard of hexamethyldisiloxane (HMDSO, Sigma-Aldrich, Burlington, MA, USA) and are reported with an accuracy of ±0.01 ppm (^1^H NMR) and ±0.1 ppm (^13^C NMR). Standard pulse sequences with 30° pulses from TopSpin 3.6.1 software were employed [45] for both ^1^H and ^13^C spectral acquisition. The spectral window was set to 7184 Hz (^1^H NMR) and 22,321 Hz (^13^C NMR), and 32,768 data points (TD) were collected. Standard 5 mm NMR tubes were used for analysis.

MS (MALDI-TOF) spectra were collected using the Shimadzu Biotech Axima Confidence system, manufactured by Shimadzu (Canby, OR, USA), using 2,5-dihydroxybenzoic acid (Macklin Biochemical Technology, Shanghai, China) as a matrix.

Elemental analysis was performed using a Flash EA1112 analyzer (Thermo Fischer Scientific, Waltham, MA, USA). Melting points were determined using a microscope MBS-10M (LZOS Inc., Lytkarino, Russia) equipped with a heated table.

### 3.4. Cells

Two cell lines obtained from the American Type Culture Collection (ATCC) were used for *in vitro* experiments: human colon adenocarcinoma HCT116 and non-tumor human embryonic kidney HEK293T cells. Cells were cultured under standard conditions in DMEM (PanEco, Moscow, Russia) supplemented with 10% fetal bovine serum (FBS; Cell Technologies, Shanghai, China), 2 mM L-glutamine (PanEco, Russia), and a mixture of 100 U mL^−1^ penicillin (PanEco, Russia) and 100 μg mL^−1^ streptomycin (PanEco, Russia). Cells were maintained at 37 °C in a humidified atmosphere containing 5% CO_2_.

For biological assays, all tested compounds were dissolved in DMSO at a concentration of 10 mM.

### 3.5. MTT Assay

Cells in the logarithmic growth phase were seeded into 96-well plates at a density of 3000 cells per well and incubated for 24 h prior to treatment. Compounds were tested at concentrations ranging from 0.1 to 50.0 µM (two-fold serial dilutions). Doxorubicin (Veropharm, Moscow, Russia) was used as a positive control. After 72 h of incubation, 20 µL of MTT reagent (5 mg mL^−1^; PanEco, Russia) was added to each well and incubated for an additional 2–3 h. The medium was then replaced with 100 µL of DMSO to dissolve the formazan crystals, and absorbance was measured at 570 nm using a Tecan Infinite 500 microplate reader (Tecan Group, Grodig, Austria). Cell survival was calculated as the absorbance of treated cells normalized to that of untreated control cells. The MTT assay was performed in three independent biological experiments, each with three technical replicates. Within each biological replicate, three technical replicates were first averaged for each concentration. The resulting mean response values were then averaged across the three independent biological experiments, and these mean values were used to fit the final dose–response curves by nonlinear regression using GraphPad Prism 8 and to calculate IC_50_ values presented as means with 95% confidence intervals [CI].

### 3.6. Antibacterial Assay

The antimicrobial activity of hydrazones **1**–**12** was assessed using an agar well diffusion method, which evaluates the diffusion of the test compound into agar and its subsequent inhibition of microbial growth. The following test microorganisms were used: *Staphylococcus aureus* ATCC 43300, *Bacillus subtilis* ATCC 6633 (Gram-positive bacteria); *Escherichia coli* ATCC 25922, *Pseudomonas aeruginosa* ATCC 27853 (Gram-negative bacteria); and *Candida albicans* ATCC 14053 (fungus). Prior to inoculation, microbial suspensions were adjusted to a 0.5 McFarland standard using a DEN-1 densitometer (Bio-San, Riga, Latvia) and evenly spread onto the surface of the agar medium in Petri dishes. Bacterial suspensions were inoculated onto Mueller-Hinton agar, while fungal suspensions were plated onto the same medium supplemented with 2% glucose. Aliquots of 100 μL of hydrazone solutions in DMSO were introduced into 9 mm wells prepared in the agar medium. After incubation at 35 °C for 24 h, the diameters of the growth inhibition zones were measured. Two concentrations of each hydrazone were tested: 10 and 1 µg mL^−1^. The effect of DMSO alone was assessed in blank control experiments, and nifuroxazide (**1**) was used as a positive control. Antibacterial activity was tested in two independent biological experiments. Representative images were included in the Supplementary Materials.

## 4. Conclusions

In this study, we performed a systematic comparative evaluation of 11 nifuroxazide analogs, which differ in the composition of the five-membered ring (5-nitrofurfural vs. 5-nitrothiophene-2-carboxaldehyde residue) and in the substitution pattern of the six-membered aromatic ring (introduction of a hydroxyl group or a heteronitrogen atom at positions 2 or 3 relative to the hydrazide bridge). Intramolecular hydrogen bonds were observed for 2-pyridinyl derivatives (weaker NH···N_heter_) and 2-hydroxyphenyl-derived hydrazones (stronger OH···O_carbonyl_).

All studied hydrazones bound to blood serum albumins and *γ*-globulins with approximately equal affinity (log *K*′ = 5.0 ± 0.3), showing no clear dependence on ligand structure or protein origin. In MTT assays, two compounds from the tested library *viz.* 5-nitrofurfurylidene hydrazide of 2-pyridinecarboxylic acid (**5**) and 5-nitrothien-2-yl hydrazide of 2-pyridinecarboxylic acid (**11**), exhibited relatively high cytotoxicity against the tumor HCT116 cell line while showing little to no toxicity toward non-tumor HEK293 cells. This selective activity makes them the most interesting candidates for further investigation, including murine cancer models.

Antimicrobial activity testing using the agar well diffusion method revealed that all 5-nitrofurfural-derived hydrazones exhibited effects comparable to those of the parent drug, nifuroxazide. Among these, two hydrazones *viz.* 5-nitrofurfurylidene hydrazide of 3-hydroxybenzoic acid (**2**) and 5-nitrofurfurylidene hydrazide of 3-pyridinecarboxylic acid (**4**), were particularly active and warrant further investigation using more complex *in vivo* models.

It should be acknowledged, however, that the results reported herein are preliminary and may not be fully recapitulated in more complex biological systems. In future work, we plan to conduct pilot *in vivo* experiments to evaluate tumor growth inhibition of selected compounds and determine their basic pharmacokinetic parameters.

## Figures and Tables

**Table 1:** ^1^H and ^13^C NMR signals (*δ*, ppm) of hydrazones **1**–**12** (peaks referred to the shared functional groups that remain the same across all compounds are shown in italics).

Nucleus	1	2	3	4	5	6	7	8	9	10	11	12
*NH*	*12.06*	*12.16*	*12.14*	*12.41*	*12.71*	*12.79*	*12.05*	*12.18*	*12.1*	*12.38*	*12.65*	*12.75*
*OH*	*10.25*	*9.82*	*11.53*				*10.25*	*9.85*	*11.53*			
*H6*	*8.38*	*8.41*	*8.43*	*8.39*	*8.63*	*8.62*	*8.66*	*8.67*	*8.68*	*8.68*	*8.86*	*8.85*
*H3*	*7.25*	*7.27*	*7.29*	*7.32*	*7.28*	*7.31*	*7.55*	*7.58*	*7.58*	*7.62*	*7.55*	*7.58*
*H4*	*7.8*	*7.81*	*7.82*	*7.82*	*7.81*	*7.81*	*8.13*	*8.14*	*8.13*	*8.14*	*8.15*	*8.14*
H11	7.82	7.34		9.08			7.81	7.34		9.07		
H12	6.88		6.97		8.75	8.97	6.88		6.96		8.74	8.96
H13		7.01	7.46	8.80	7.71	8.82		7.01	7.45	8.79	7.71	8.82
H14		7.30	7.00	7.60	8.08			7.29	6.99	7.59	8.08	
H15		7.35	7.83	8.27	8.16	9.30		7.33	7.83	8.26	8.14	9.28
*C9*	*161.6*	*163.9*	*165.1*	*162.5*	*161.3*	*160.4*	*163.4*	*163.8*	*165.0*	*162.5*	*161.2*	*160.4*
*C6*	*134.9*	*134.6*	*136.5*	*136.6*	*138.6*	*137.9*	*140.6*	*141.5*	*142.2*	*142.3*	*142.8*	*143.4*
*C5*	*152.5*	*152.4*	*152.5*	*152.5*	*152.4*	*152.5*	*151.0*	*151.3*	*151.5*	*151.5*	*151.4*	*151.6*
*C2*	*152.3*	*152.3*	*152.1*	*151.9*	*149.5*	*152.0*	*147.6*	*147.3*	*146.9*	*149.1*	*149.5*	*148.6*
*C3*	*115.2*	*115.0*	*115.1*	*115.1*	*115.1*	*115.1*	*129.7*	*129.4*	*129.5*	*130.5*	*130.2*	*130.5*
*C4*	*115.6*	*115.7*	*116.1*	*116.3*	*115.6*	*116.1*	*130.4*	*130.1*	*130.4*	*131.0*	*131.0*	*130.9*
*C10*	*115.4*	*135.9*	*117.0*	*129.1*	*152.3*	*144.6*	*115.6*	*134.7*	*117.0*	*129.2*	*147.2*	*146.8*
C11	130.4	115.2	158.8	149.2			131.0	115.0	158.8	146.8		
C12	123.6	158.0	117.6		149.1	143.8	123.7	157.9	117.6		149.1	144.7
C13	152.5	118.7	134.5	153.2	127.9	148.7	161.5	118.9	134.4	153.1	127.8	144.8
C14		130.2	119.6	124.2	137.3			131.0	119.6	124.1	138.6	
C15		119.6	129.6	136.1	123.5	144.8		119.6	131.0	136.0	123.5	143.9

**Table 2:** Conditional equilibrium constants of the reaction between BSA, HSA, BGG, HGG and hydrazones **1**–**12** determined at pH 6.86, *T* = 298.2 K, *p* = 0.1 MPa, *I* = 0.05 M.

Hydrazone	1	2	3	4	5	6	7	8	9	10	11	12
BSA	5.5 ± 0.2 *	5.5 ± 0.1	5.4 ± 0.1	5.1 ± 0.1	5.2 ± 0.2	5.6 ± 0.4	5.4 ± 0.1	5.3 ± 0.1	5.5 ± 0.1	5.0 ± 0.2	5.0 ± 0.2	4.8 ± 0.1
HSA	5.0 ± 0.1	5.1 ± 0.1	5.2 ± 0.1	5.2 ± 0.2	5.2 ± 0.1	5.0 ± 0.2	5.3 ± 0.1	5.3 ± 0.3	5.4 ± 0.1	4.9 ± 0.1	4.7 ± 0.2	4.8 ± 0.2
BGG	4.9 ± 0.2	4.9 ± 0.1	4.9 ± 0.1	4.8 ± 0.1	5.0 ± 0.1	4.9 ± 0.2	4.9 ± 0.3	4.7 ± 0.2	5.0 ± 0.2	4.8 ± 0.3	5.0 ± 0.3	5.1 ± 0.1
HGG	5.0 ± 0.1	5.1 ± 0.3	4.8 ± 0.1	4.8 ± 0.2	4.8 ± 0.2	4.9 ± 0.1	4.9 ± 0.2	4.8 ± 0.2	4.8 ± 0.2	4.4 ± 0.1	4.9 ± 0.2	5.2 ± 0.2

* Errors given are the standard deviations calculated for three independent experiments.

**Table 3:** IC_50_ values (µM) [CI] of hydrazones **1**–**12** determined for HCT116 and HEK293T cell lines.

Cell Line	1	2	3	4	5	6	7	8	9	10	11	12
HCT116	5.5 [4.1–7.5]	4.5 [3.4–6.1]	9.5 [7.0–12.8]	10.5 [8.1–13.3]	4.9 [3.4–6.9]	>50	26.8 [18.3–40.2]	7.3 [5.1–10.4]	18.8 [11.6–31.1]	28.6 [19.9–40.2]	10.4 [5.7–18.8]	22.6 [13.5–37.0]
HEK293	16.0 [13.2–19.3]	11.3 [9.3–13.7]	26.3 [20.2–35.0]	20.4 [16.2–25.5]	42.9 [36.5–51.2]	>50	>50	23.1 [17.3–30.6]	>50	>50	>50	>50
SI *	2.9	2.5	2.8	1.9	8.8	n/a **	>1.9	3.2	>2.7	>1.7	>4.8	>2.2

* SI, selectivity index: SI = IC_50_(HEK293)/IC_50_(HCT116). ** n/a, not available.

**Table 4:** Diameters of growth inhibition (mm) zones caused by hydrazones **1**–**12**. The standard deviation of zone diameter measurements does not exceed ±1 mm.

Hydrazone	Concentration, µg mL^−1^	Microorganism					pH
		*S. aureus* ATCC 43300	*B. subtilis* ATCC 6633	*E. coli* ATCC 25922	*P. aeruginosa* ATCC 27853	*C. albicans* ATCC 14053	
**1**	10	32	25	15	no effect	no effect	6.5
	1	32	24	15	no effect	no effect	
**2**	10	45	34	25	15	no effect	6.8
	1	34	31	17	no effect	no effect	
**3**	10	30	34	14	12	no effect	6.5
	1	28	35	14	no effect	no effect	
**4**	10	45	38	28	16	no effect	7.0
	1	35	34	21	16	no effect	
**5**	10	30	35	no effect	no effect	no effect	6.8
	1	25	32	no effect	no effect	no effect	
**6**	10	32	34	16	no effect	no effect	6.5
	1	33	37	20	no effect	no effect	
**7**	10	28	22	14	no effect	no effect	6.8
	1	24	15	13	no effect	no effect	
**8**	10	28	20	13	no effect	no effect	7.0
	1	26	22	14	no effect	no effect	
**9**	10	18	17	no effect	no effect	no effect	6.9
	1	no effect	no effect	no effect	no effect	no effect	
**10**	10	28	24	14	no effect	no effect	7.0
	1	31	27	17	no effect	no effect	
**11**	10	12	12	no effect	no effect	no effect	7.0
	1	10	10	no effect	no effect	no effect	
**12**	10	17	16	no effect	no effect	no effect	7.0
	1	12	12	no effect	no effect	no effect	

## Data Availability

The original contributions presented in the study are included in the article/Supplementary Materials. Further inquiries can be directed to the corresponding author.

## Supplementary Materials

The following supporting information can be downloaded at: https://www.mdpi.com/article/10.3390/ijms27187983/s1.

## Abbreviations

The following abbreviations are used in this manuscript:

ACE2	Angiotensin-Converting Enzyme 2
ALP	Alkaline Phosphatase
Ang1-7	Angiotensin 1-7
AST	Aspartate Aminotransferase
ATCC	American Type Culture Collection
BGG	Bovine *γ*-globulin
BSA	Bovine serum albumin
CaCo-2	Immortalized human colorectal adenocarcinoma cell line
CI	Confidence interval
CK-MB	Creatine Kinase-MB
CRP	C-Reactive Protein
DMSO	Dimethyl sulfoxide
DMEM	Dulbecco’s Modified Eagle Medium
HCT116	Human colorectal carcinoma cell line
HEK293	Immortalized human embryonic kidney cell line
HepG-2	Immortalized human liver cancer cell line
HGG	Human *γ*-globulin
HMDSO	Hemamethyldisiloxane
HMGB1	High Mobility Group Box 1 Protein
HSA	Human serum albumin
IC_50_	Half-Maximal Inhibitory Concentration
IL-1β	Interleukin-1 Beta
IR	Infrared
JAK2	Janus Kinase 2
LDH	Lactate Dehydrogenase
MAS	Mas Receptor (G protein-coupled receptor)
MDA-MB-231	Human breast cancer cell line
MTT	3-(4,5-dimethylthiazol-2-yl)-2,5-diphenyltetrazolium bromide
NF-κB	Nuclear Factor Kappa B
NLRP3	NOD-Like Receptor Family Pyrin Domain Containing 3
NMR	Nuclear magnetic resonance
Ph	Phenyl
PPAR-γ	Peroxisome Proliferator-Activated Receptor Gamma
Py	Pyridine
Pz	Pyrazine
SI	Selectivity Index
STAT1/3/5	Signal Transducer and Activator of Transcription 1/3/5
TLR4	Toll-Like Receptor 4
TNF-α	Tumor Necrosis Factor-Alpha
TYK2	Tyrosine Kinase 2
Wi-38	Diploid human cell line
