# Supplementary material for: Synthesis, Cytotoxicity and Antimicrobial Action of 11 Analogs of Nifuroxazide
Source: Int J Mol Sci. 2026 Sep 8;27(18):7983. doi: 10.3390/ijms27187983 (PMC13606672; doi:10.3390/ijms27187983)
Supplement: Supplementary file 1 [file ijms-27-07983-s001.zip › ijms-4529119-supplementary.pdf]

Supporting Information for

Synthesis, Cytotoxicity and Antimicrobial Action of 11 Analogs of Nifuroxazide

Daniil N. Yarullin [1], Olga I. Logacheva [1], Aleksandra K. Isagulieva [2], Olga N. Sineva [3], Vera S. Sadykova [3], Maksim N. Zavalishin [1,*] and George A. Gamov [1]

[1] General Chemical Technology Department, Ivanovo State University of Chemistry and Technology, Sheremetevskii Ave. 7, 153000 Ivanovo, Russia; dn_yarullin@isuct.ru (D.N.Y.); o.logachiova@yandex.ru (O.I.L.); ggamov@isuct.ru (G.A.G.)

[2] Burnazyan Federal Medical Biophysical Center, Federal Medical Biological Agency, Marshal Novikov St., 23, 123098 Moscow, Russia; kia2303@yandex.ru

[3] Gause Institute of New Antibiotics, Bol'shaya Pirogovskaya St. 11/1, 119021 Moscow, Russia; olga.sineva81@yandex.ru (O.N.S.); sadykova_09@mail.ru (V.S.S.)

* Correspondence: zavalishin00@gmail.com; Tel.: +7-(4932)30-73-46

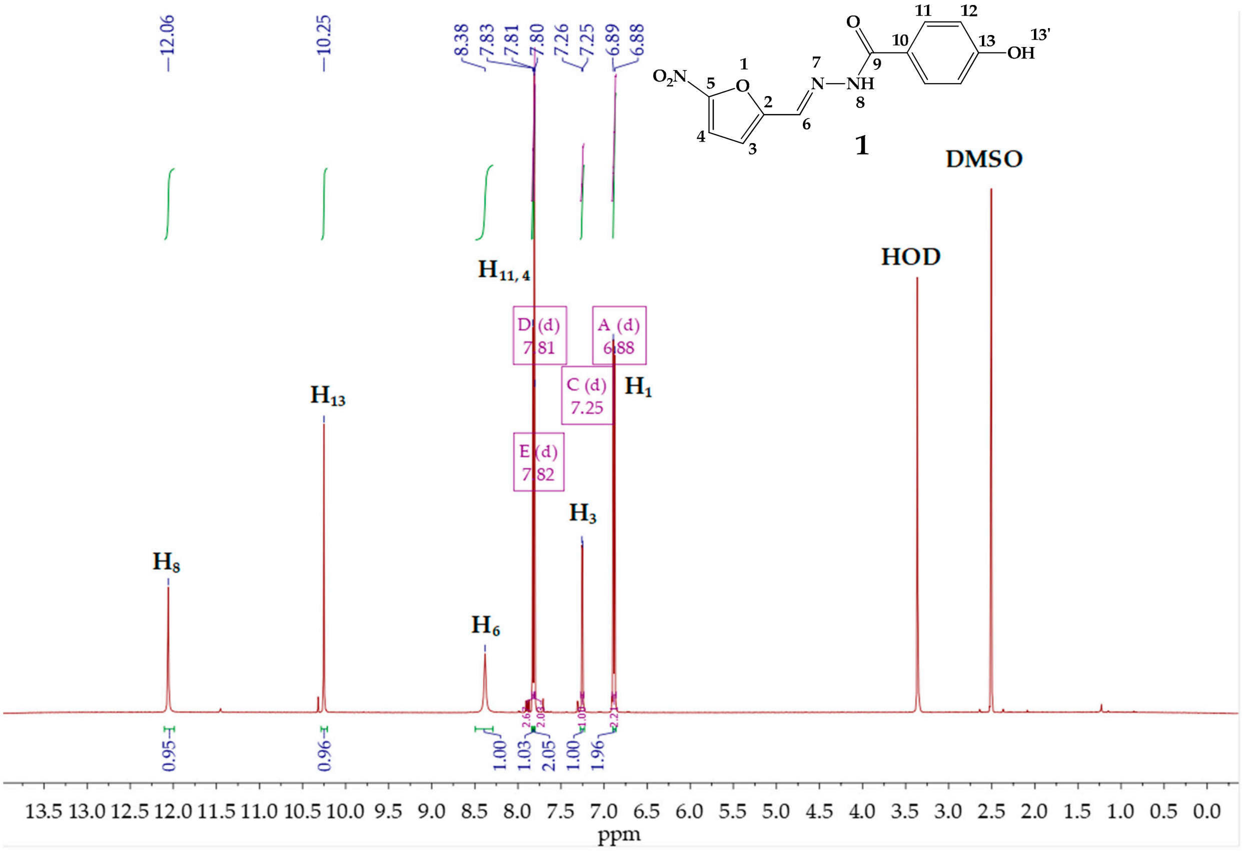

12.06
10.25
8.38
7.83
7.81
7.80
7.26
7.25
6.89
6.88
O
11
12
13
13'
OH
10
9
7
N
NH
8
O_2N
5
O
1
2
6
4
3
1
DMSO
HOD
$H_{11, 4}$
D (d) 7.81
A (d) 6.88
C (d) 7.25
H_1
H_{13}
E (d) 7.82
H_3
H_8
H_6
0.95
0.96
1.00
1.03
2.05
1.00
1.96
13.5 13.0 12.5 12.0 11.5 11.0 10.5 10.0 9.5 9.0 8.5 8.0 7.5 7.0 6.5 6.0 5.5 5.0 4.5 4.0 3.5 3.0 2.5 2.0 1.5 1.0 0.5 0.0
ppm

a

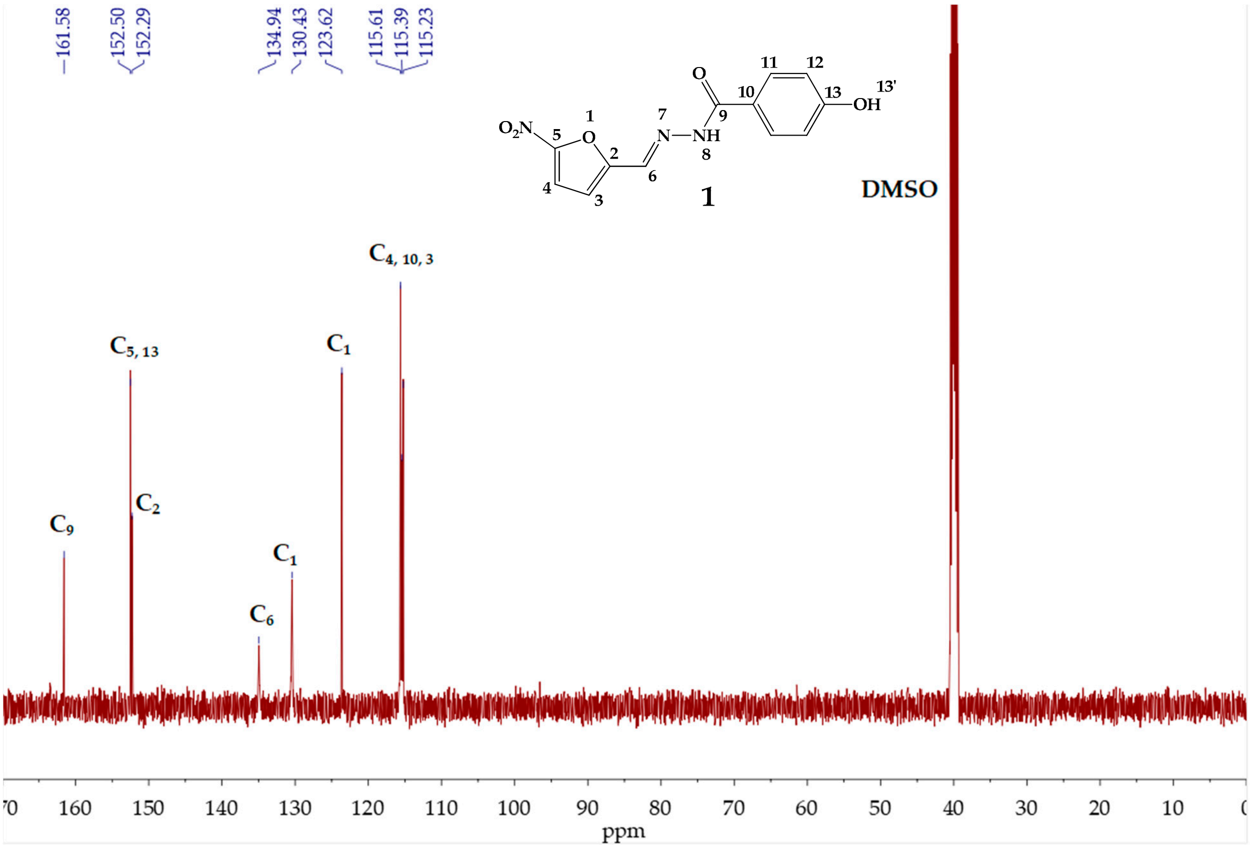

161.58
152.50
152.29
134.94
130.43
123.62
115.61
115.39
115.23
O
11
12
10
13
13'
OH
9
7
N
NH
8
O_2N
5
O
1
2
6
4
3
1
DMSO
$C_{4, 10, 3}$
$C_{5, 13}$
C_1
C_2
C_9
C_1
C_6
160
150
140
130
120
110
100
90
80
70
60
50
40
30
20
10
ppm

b

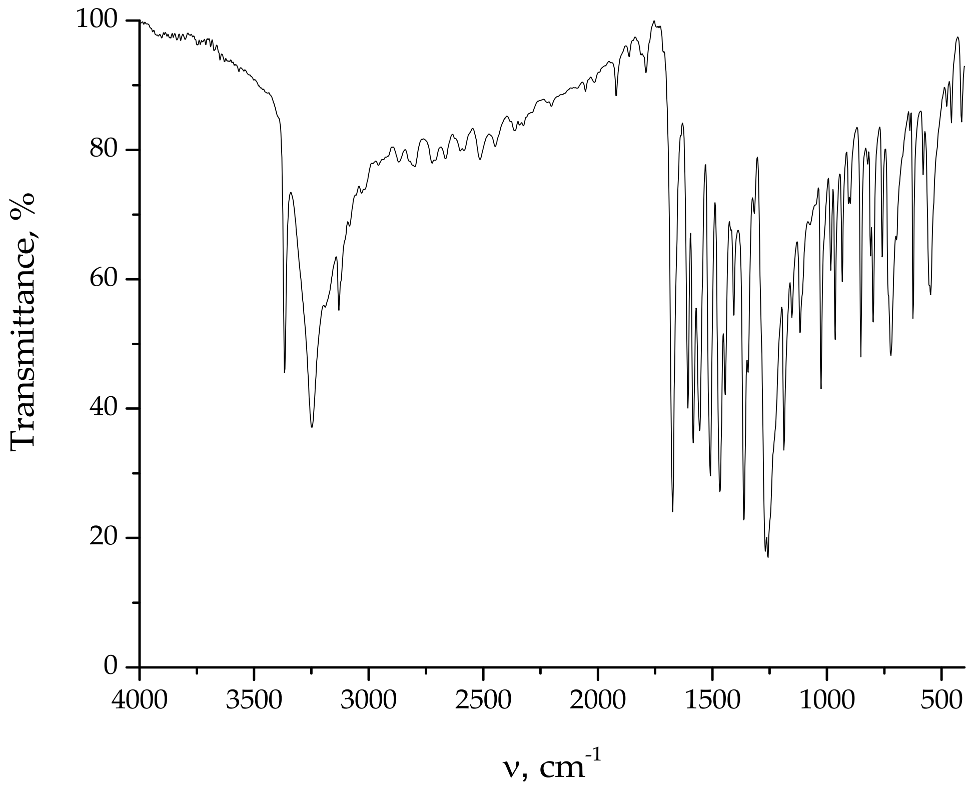

c

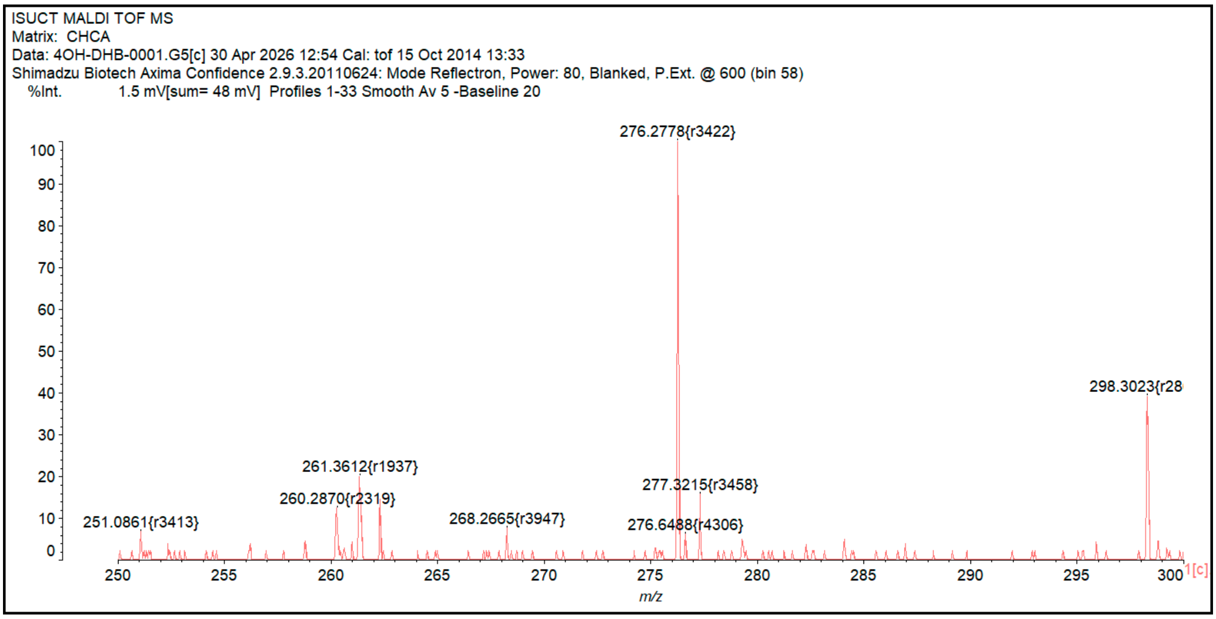

d

Figure S1. ^{1}H (a), ^{13}C (b) NMR, IR (c) and mass (d) spectra of 4-hydroxy-N'-((5-nitrofuran-2-yl)methylene)benzohydrazide (nifuroxazide) **1**

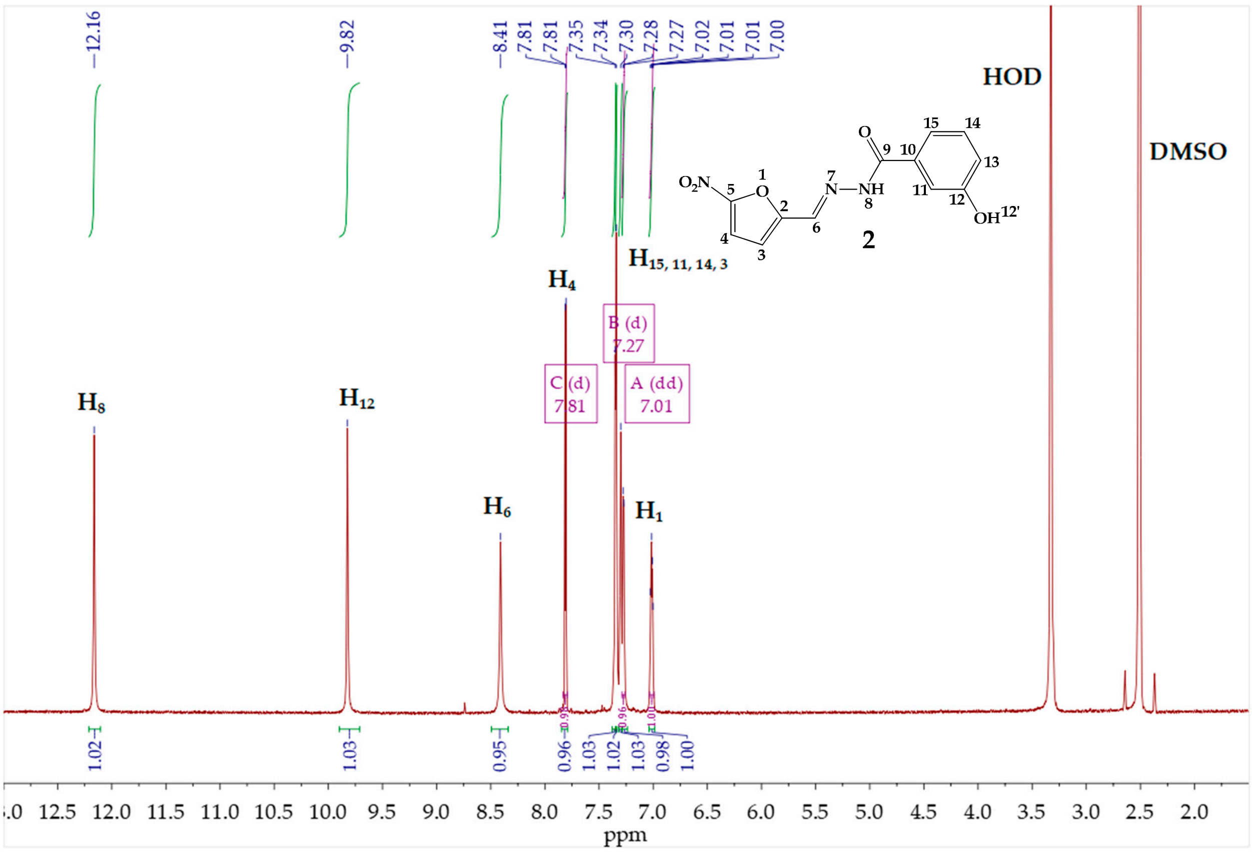

12.16
9.82
8.41
7.81
7.81
7.35
7.34
7.30
7.28
7.27
7.02
7.01
7.01
7.00
HOD
DMSO
O
15
14
9
10
O_2N
5
1
O
7
N—NH
8
11
13
12
OH12'
2
4
6
3
2
$H_{15, 11, 14, 3}$
H_4
B (d)
7.27
C (d)
7.81
A (dd)
7.01
H_8
H_{12}
H_6
H_1
1.02
1.03
0.95
0.96
1.03
1.02
1.03
0.98
1.00
12.5 12.0 11.5 11.0 10.5 10.0 9.5 9.0 8.5 8.0 7.5 7.0 6.5 6.0 5.5 5.0 4.5 4.0 3.5 3.0 2.5 2.0
ppm

a

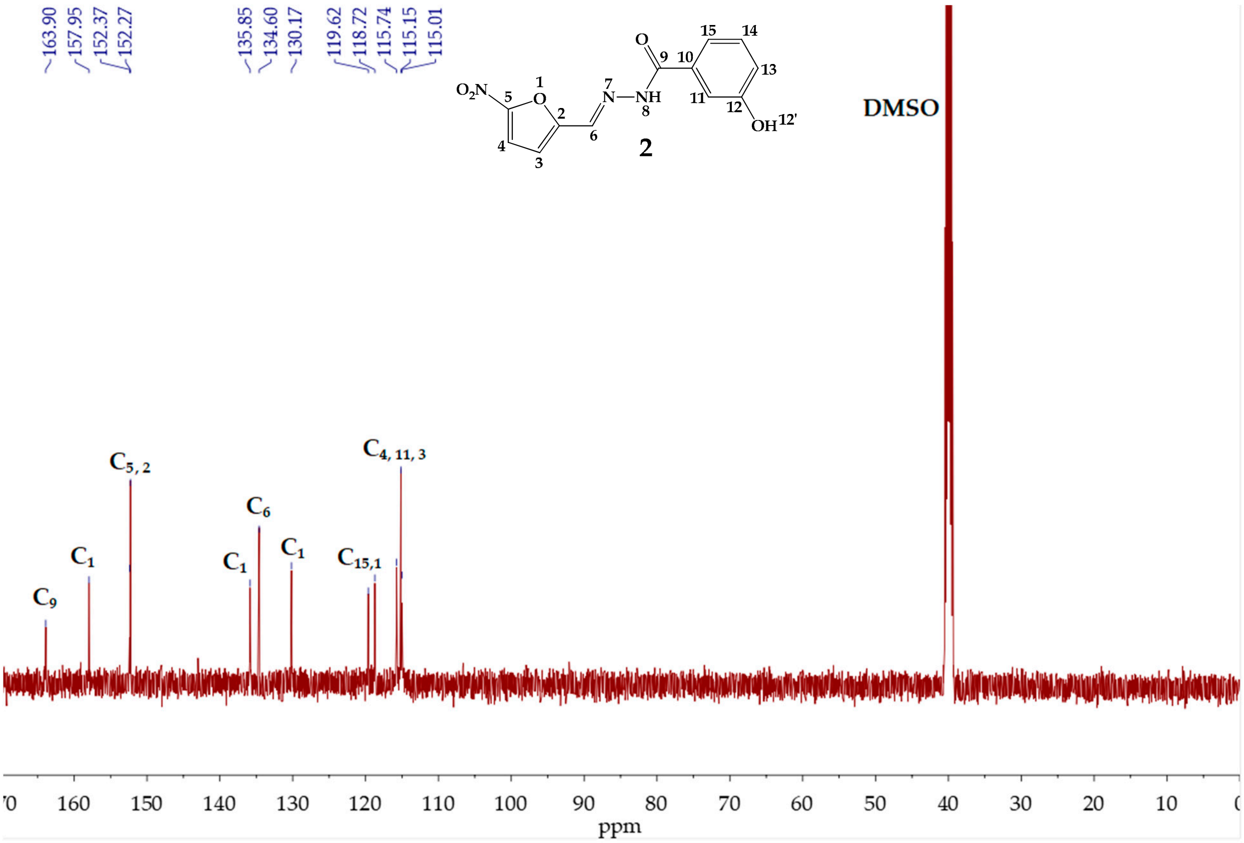

163.90
157.95
152.37
152.27
135.85
134.60
130.17
119.62
118.72
115.74
115.15
115.01
O
15
14
9
10
13
O_2N
5
1
O
7
N—NH
8
11
12
OH12'
2
4
6
3
2
DMSO
$C_{5, 2}$
$C_{4, 11, 3}$
C_6
C_1
C_1
C_1
$C_{15,1}$
C_9
160 150 140 130 120 110 100 90 80 70 60 50 40 30 20 10
ppm

b

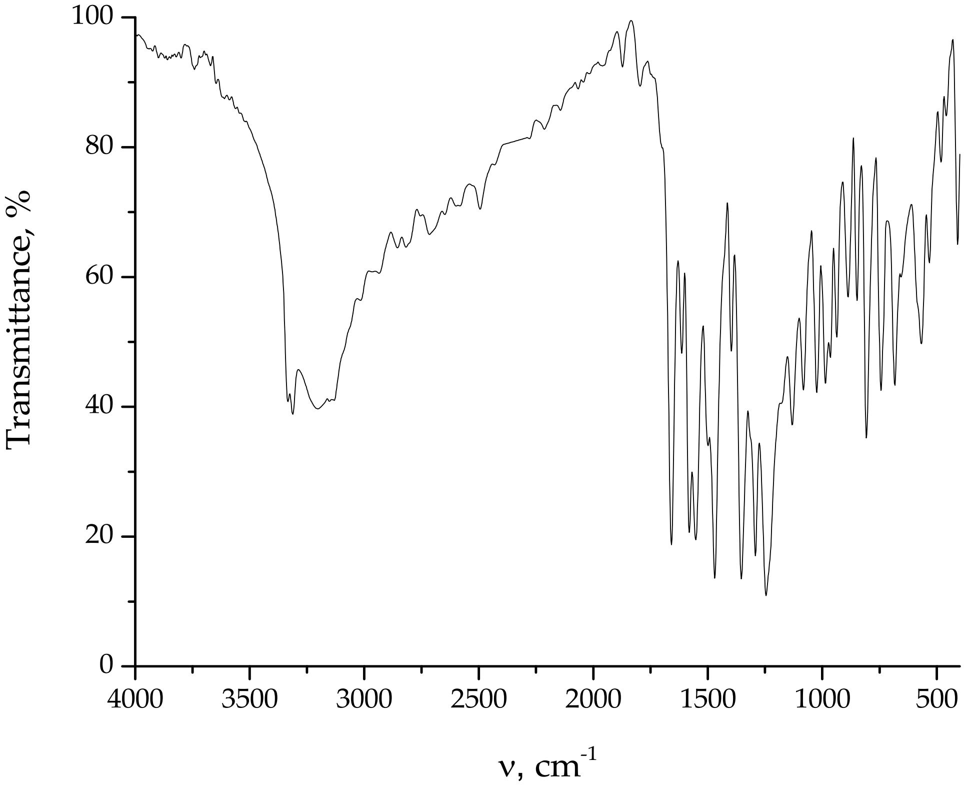

c

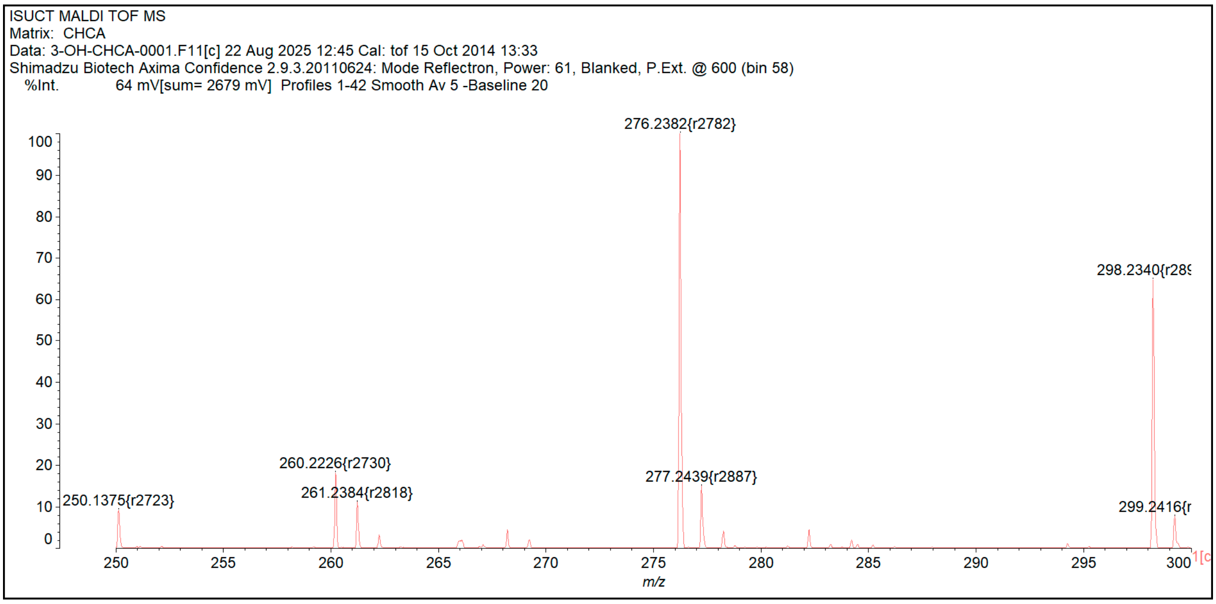

d

Figure S2. ^{1}H (a), ^{13}C (b) NMR, IR (c) and mass (d) spectra of 3-hydroxy-N'-((5-nitrofuran-2-yl)methylene)benzohydrazide **2**

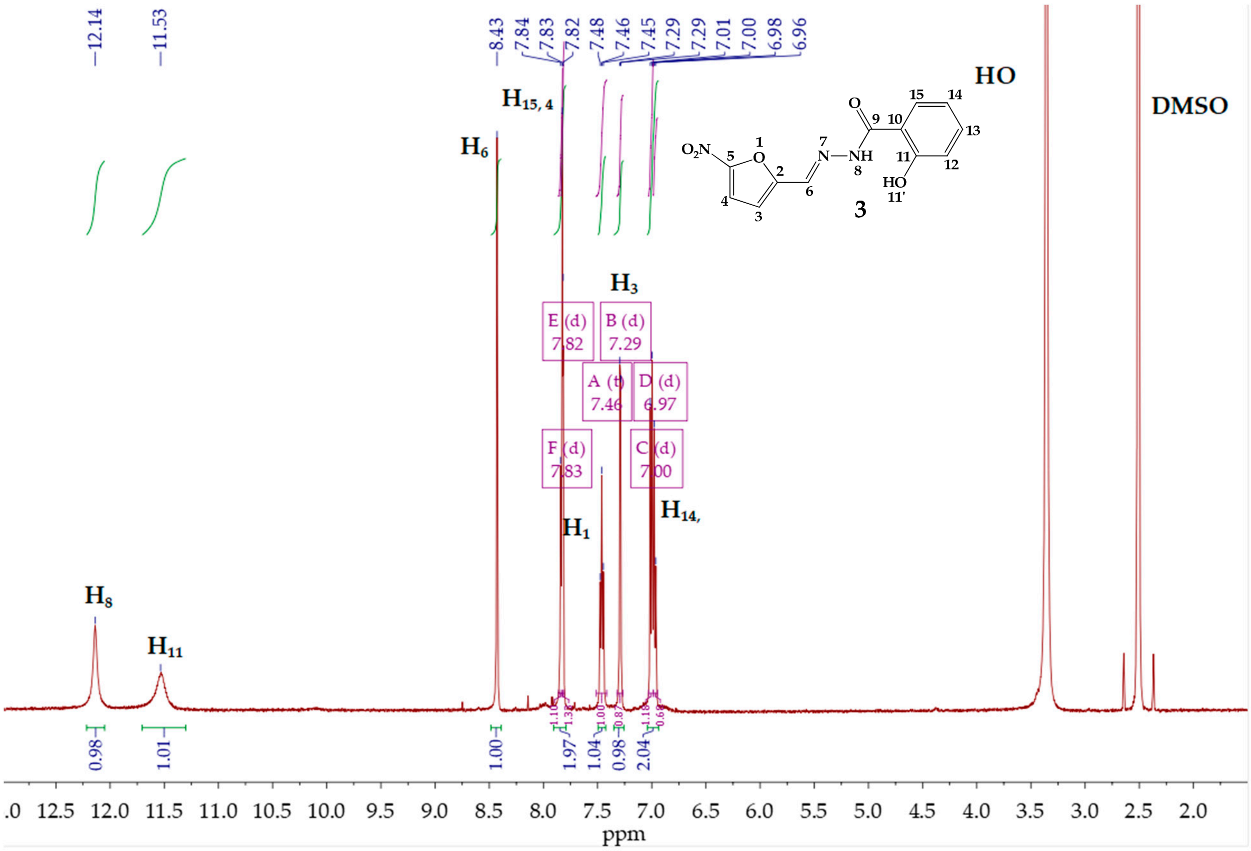

12.14
11.53
8.43
7.84
7.83
7.82
7.48
7.46
7.45
7.29
7.29
7.01
7.00
6.98
6.96
H15, 4
H6
HO
DMSO
O2N
3
H3
E (d) 7.82
B (d) 7.29
A (t) 7.46
D (d) 6.97
F (d) 7.83
C (d) 7.00
H1
H14,
H8
H11
0.98
1.01
1.00
1.97
1.04
0.98
2.04
ppm

a

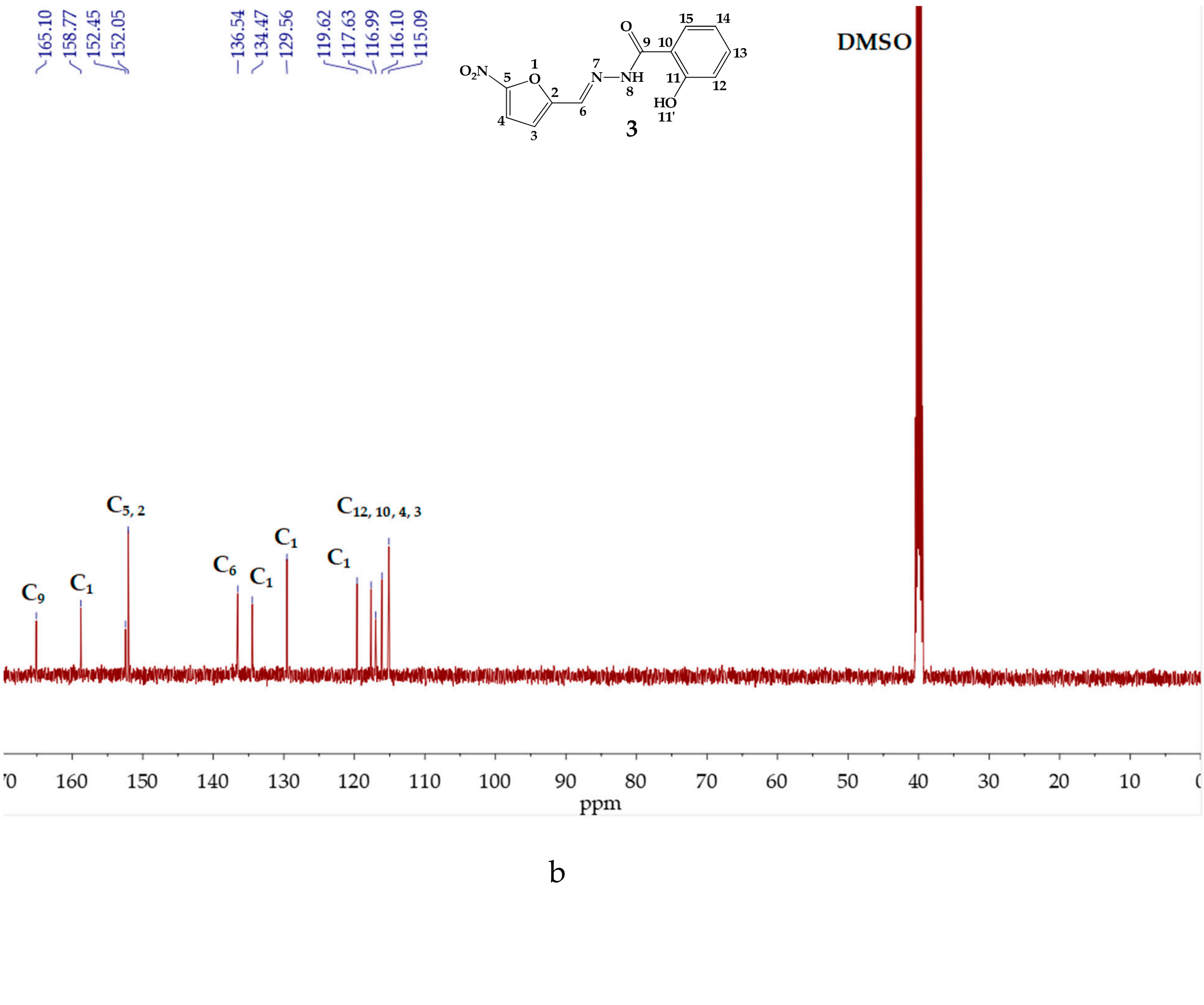

165.10
158.77
152.45
152.05
136.54
134.47
129.56
119.62
117.63
116.99
116.10
115.09
O2N
3
DMSO
C9
C1
C5, 2
C6
C1
C1
C1
C12, 10, 4, 3
ppm

b

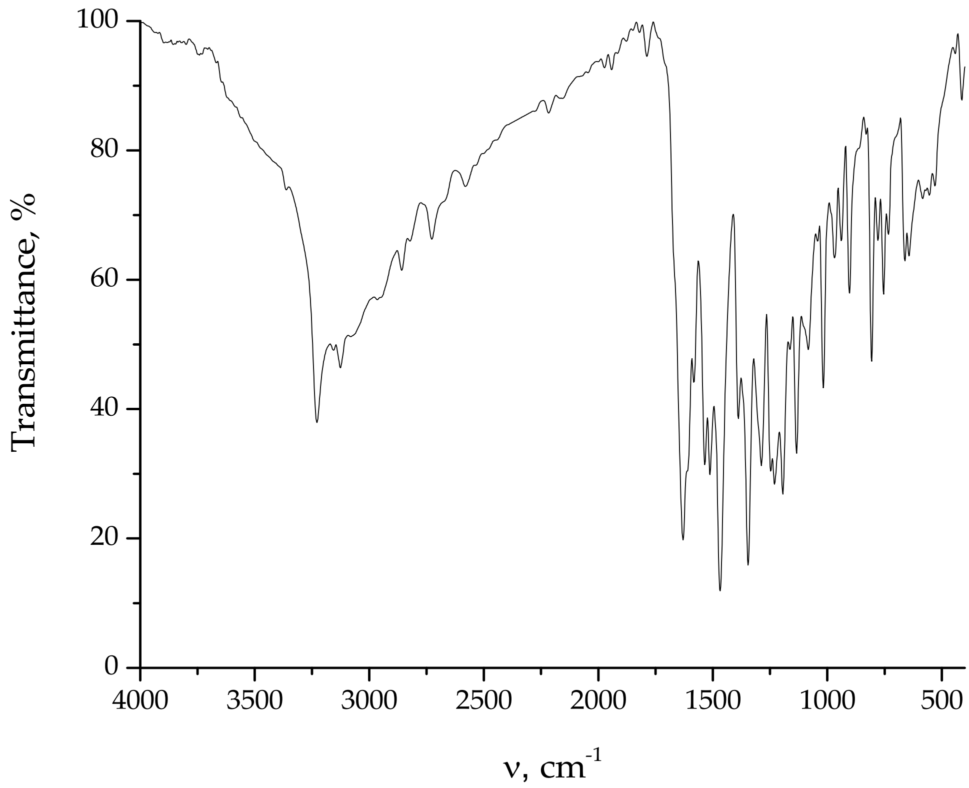

c

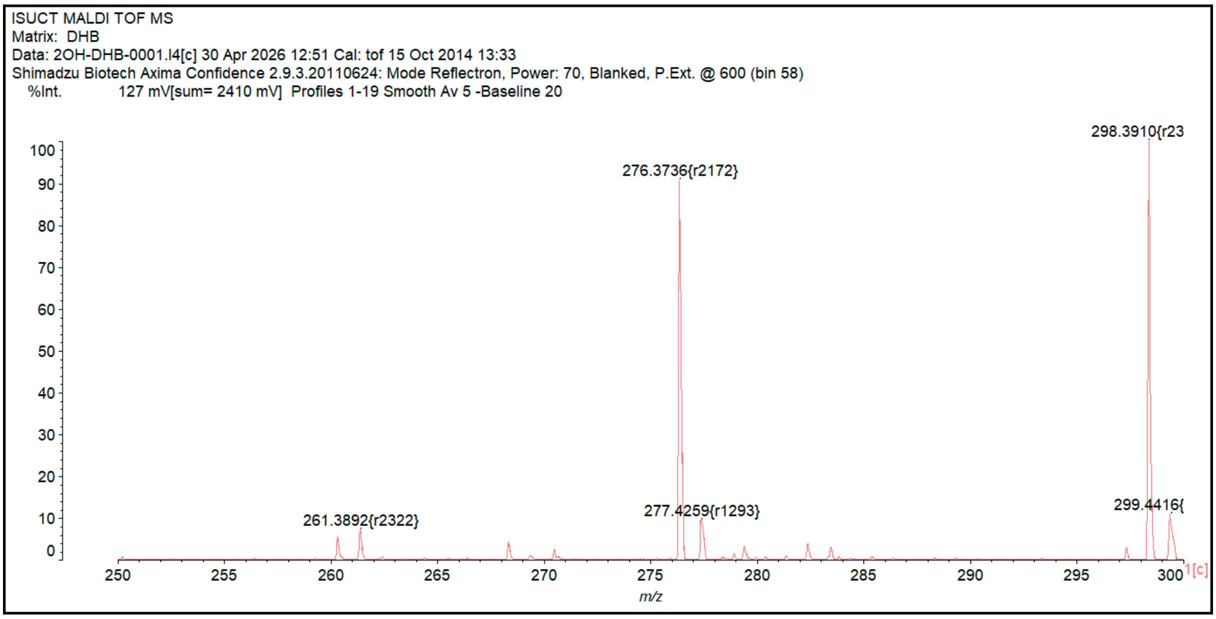

d

Figure S3. ^{1}H (a), ^{13}C (b) NMR, IR (c) and mass (d) spectra of 2-hydroxy-N'-((5-nitrofuran-2-yl)methylene)benzohydrazide **3**

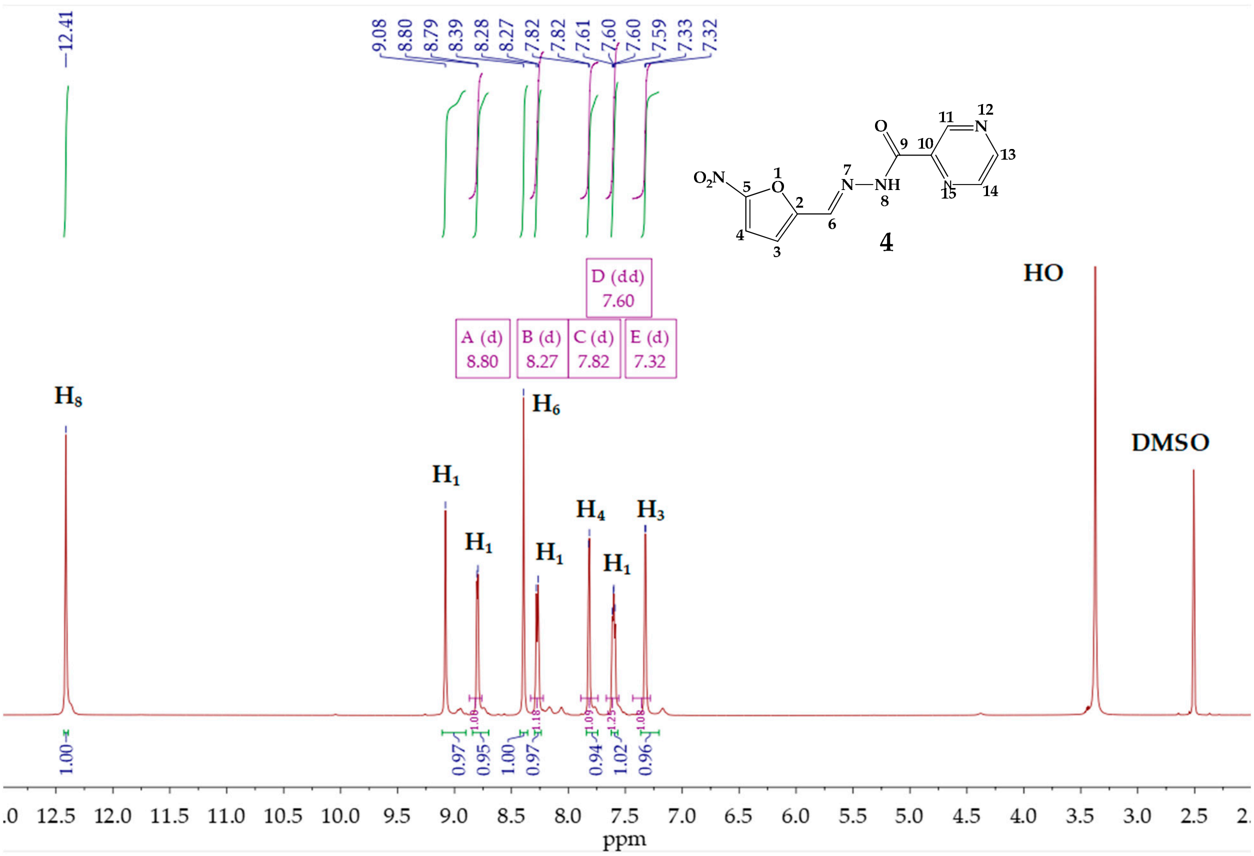
12.41
9.08 8.80 8.79 8.39 8.28 8.27 7.82 7.82 7.61 7.60 7.60 7.59 7.33 7.32
4
D (dd) 7.60
A (d) 8.80
B (d) 8.27
C (d) 7.82
E (d) 7.32
HO
DMSO
H_8
H_1
H_6
H_4
H_3
1.00
0.97
0.95
1.00
0.97
0.94
1.02
0.96
ppm

a

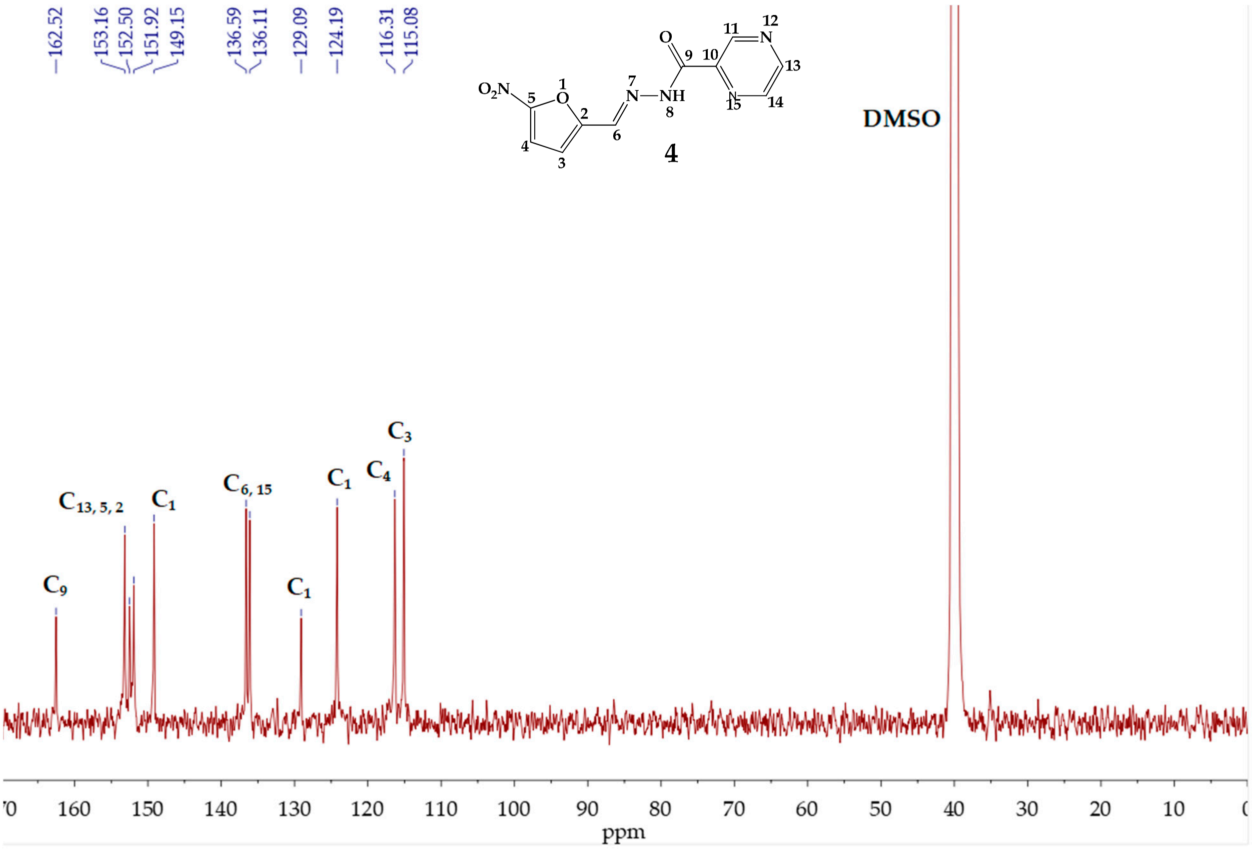
162.52
153.16 152.50 151.92 149.15
136.59 136.11
129.09
124.19
116.31 115.08
4
DMSO
C_9
$C_{13, 5, 2}$
C_1
$C_{6, 15}$
C_4
C_3
ppm

b

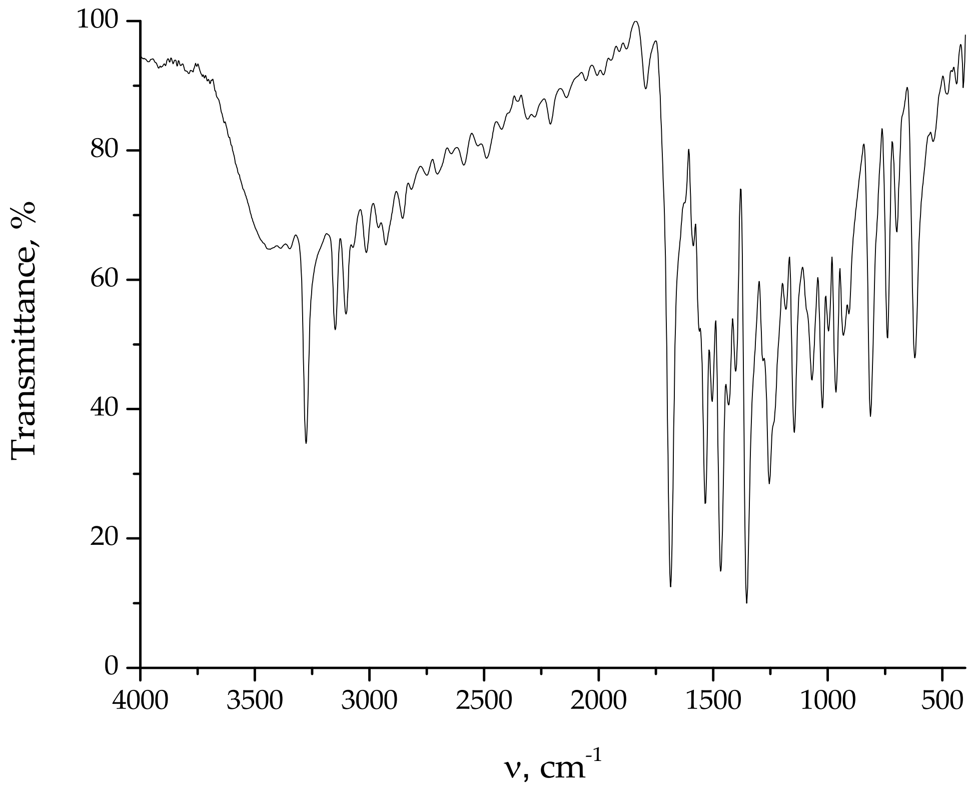

c

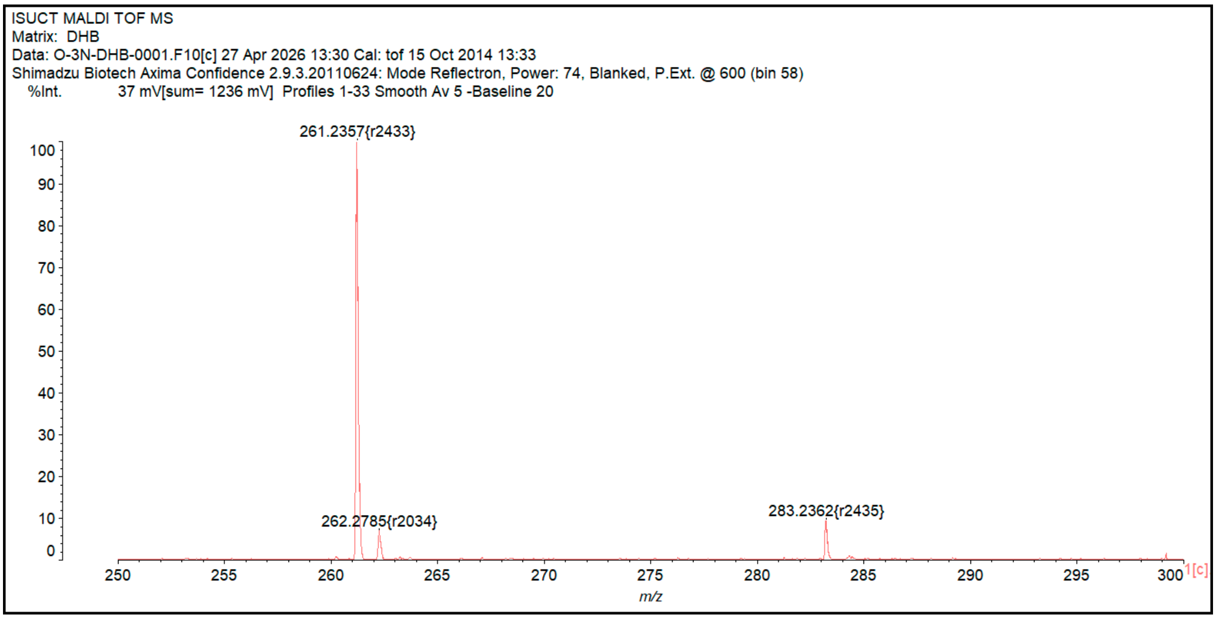

d

Figure S4. ^{1}H (a), ^{13}C (b) NMR, IR (c) and mass (d) spectra of N'-((5-nitrofuran-2-yl)methylene)pyridine-3-carbohydrazide **4**

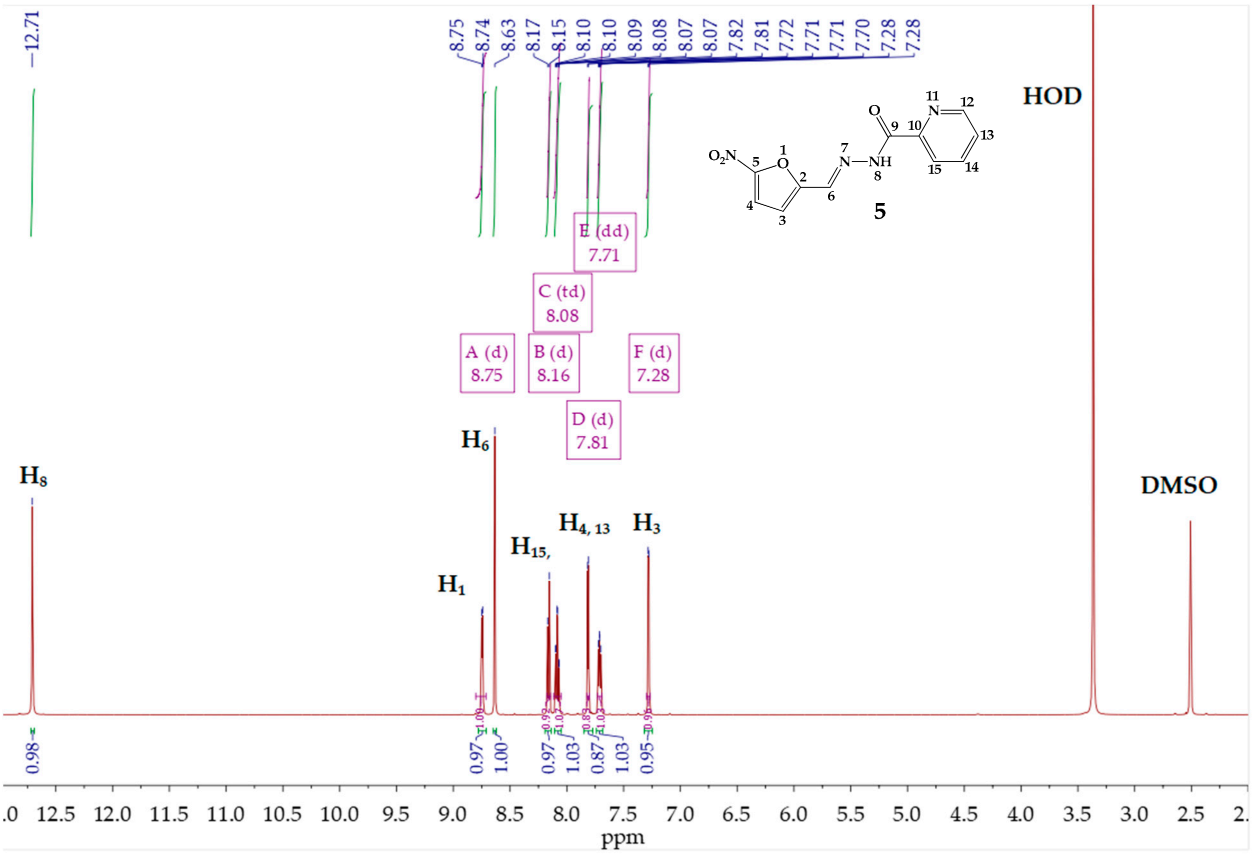

HOD
DMSO
H8
H6
H1
H15,
H4, 13
H3
A (d) 8.75
B (d) 8.16
C (td) 8.08
D (d) 7.81
E (dd) 7.71
F (d) 7.28
5
ppm

a

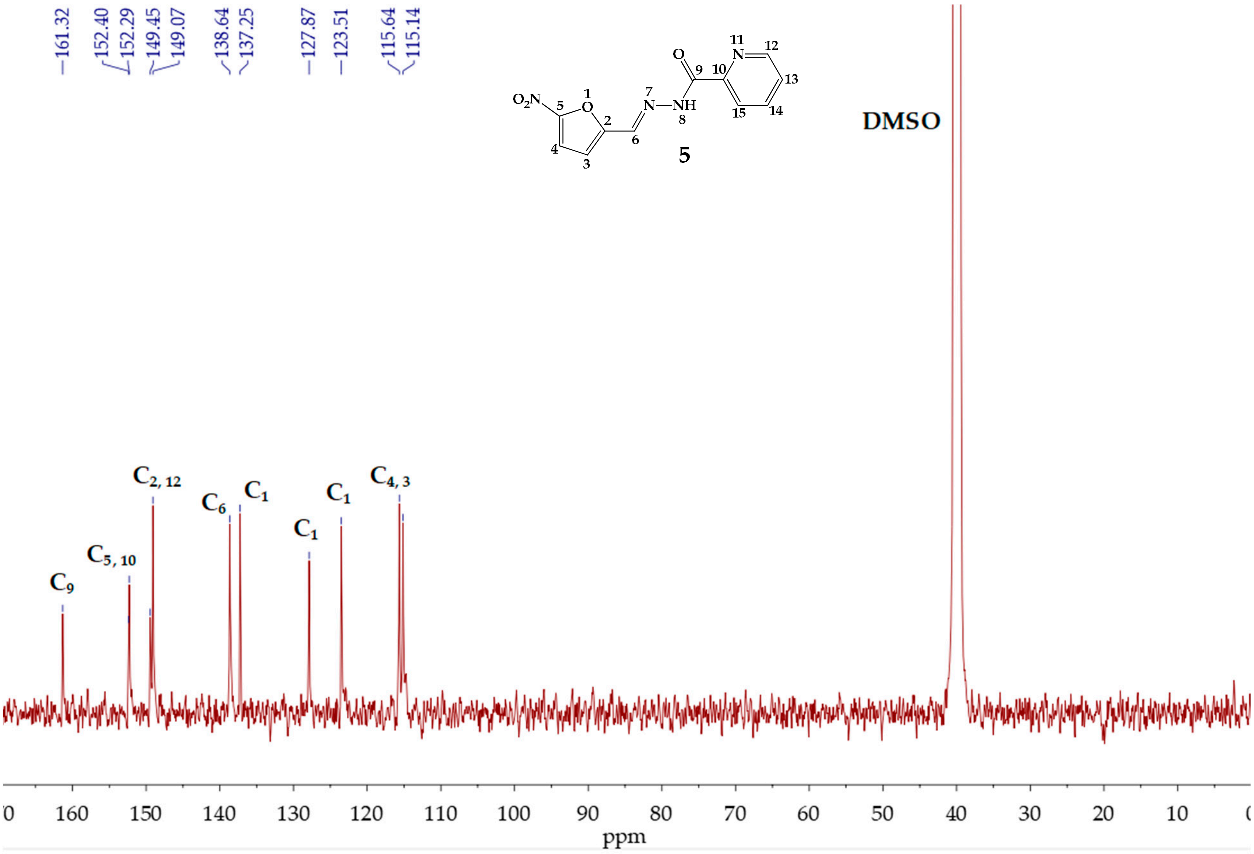

DMSO
C9
C5, 10
C2, 12
C6
C1
C1
C1
C4, 3
5
ppm

b

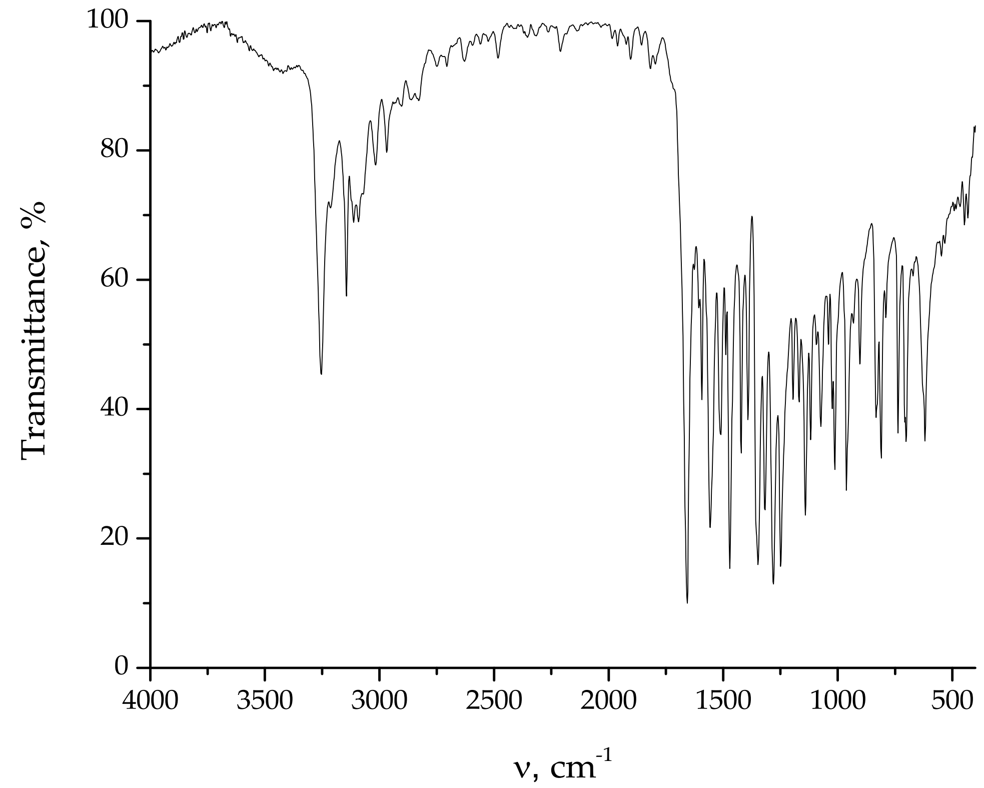

c

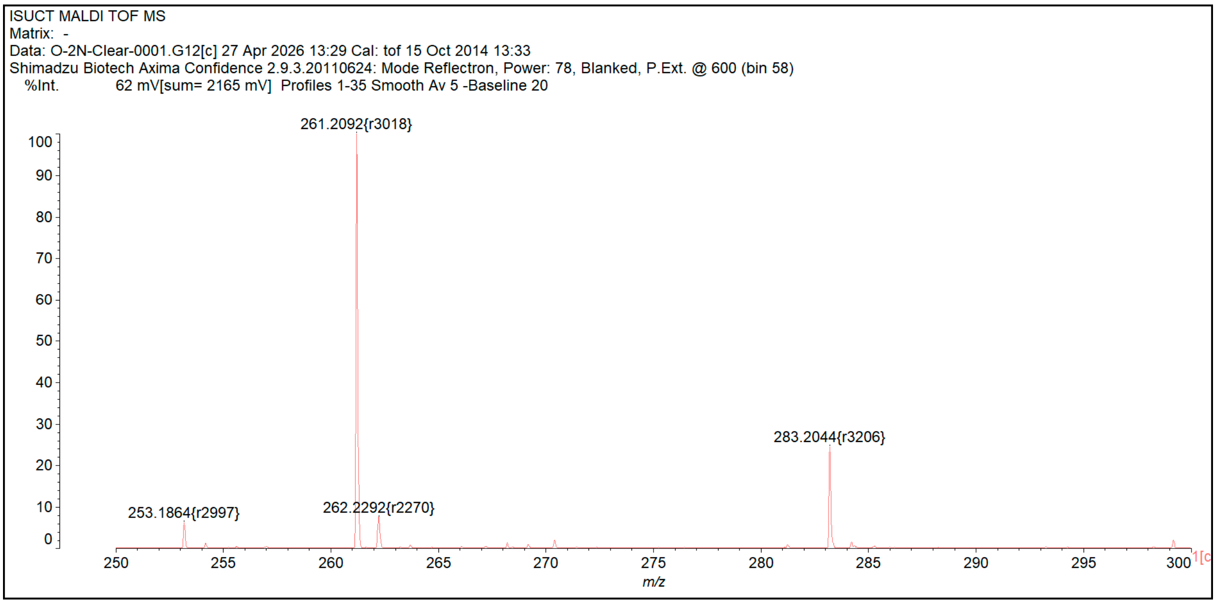

d

Figure S5. ^{1}H (a), ^{13}C (b) NMR, IR (c) and mass (d) spectra of N'-((5-nitrofuran-2-yl)methylene)pyridine-2-carbohydrazide **5**

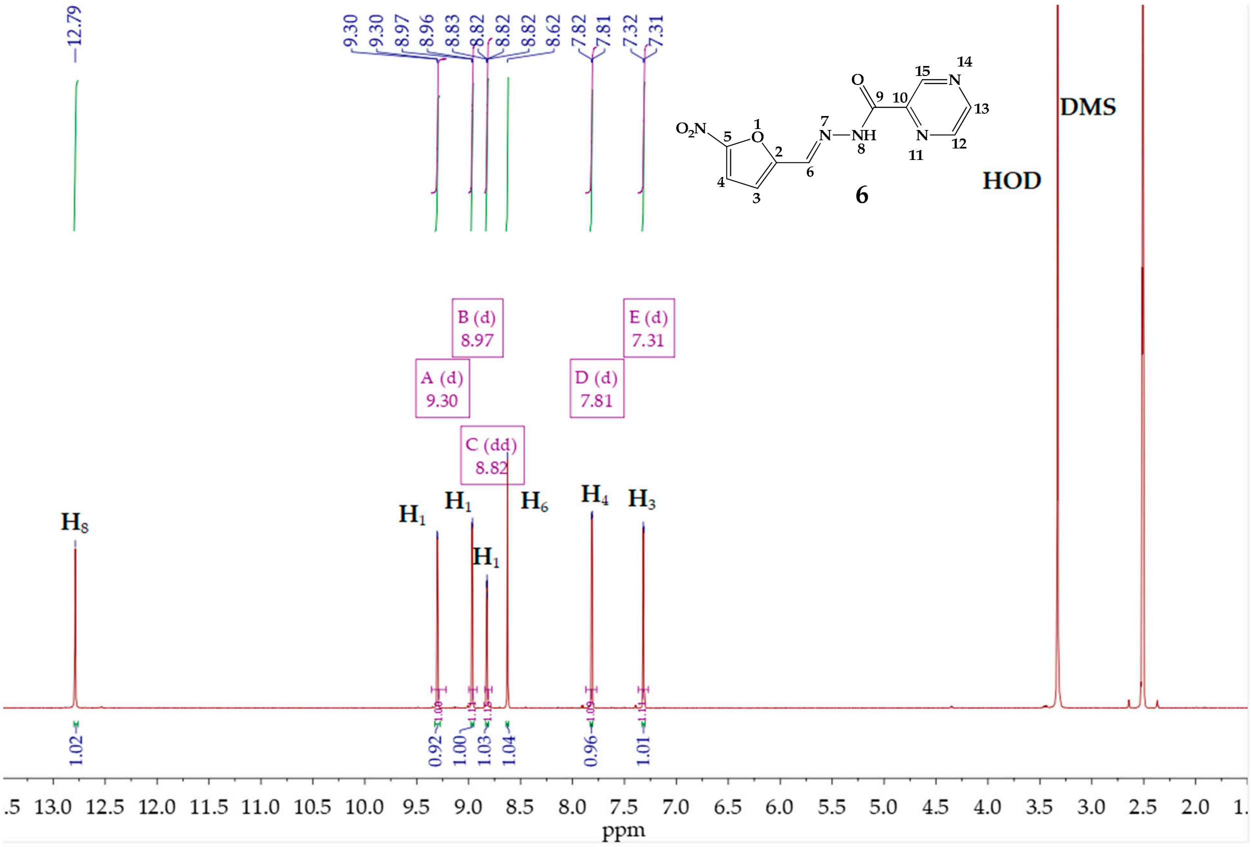
12.79
9.30 9.30 8.97 8.96 8.83 8.82 8.82 8.82 8.62 7.82 7.81 7.32 7.31
DMS
HOD
6
A (d) 9.30
B (d) 8.97
C (dd) 8.82
D (d) 7.81
E (d) 7.31
H8
H1
H1
H1
H6
H4
H3
1.02
0.92
1.00
1.03
1.04
0.96
1.01
ppm

a

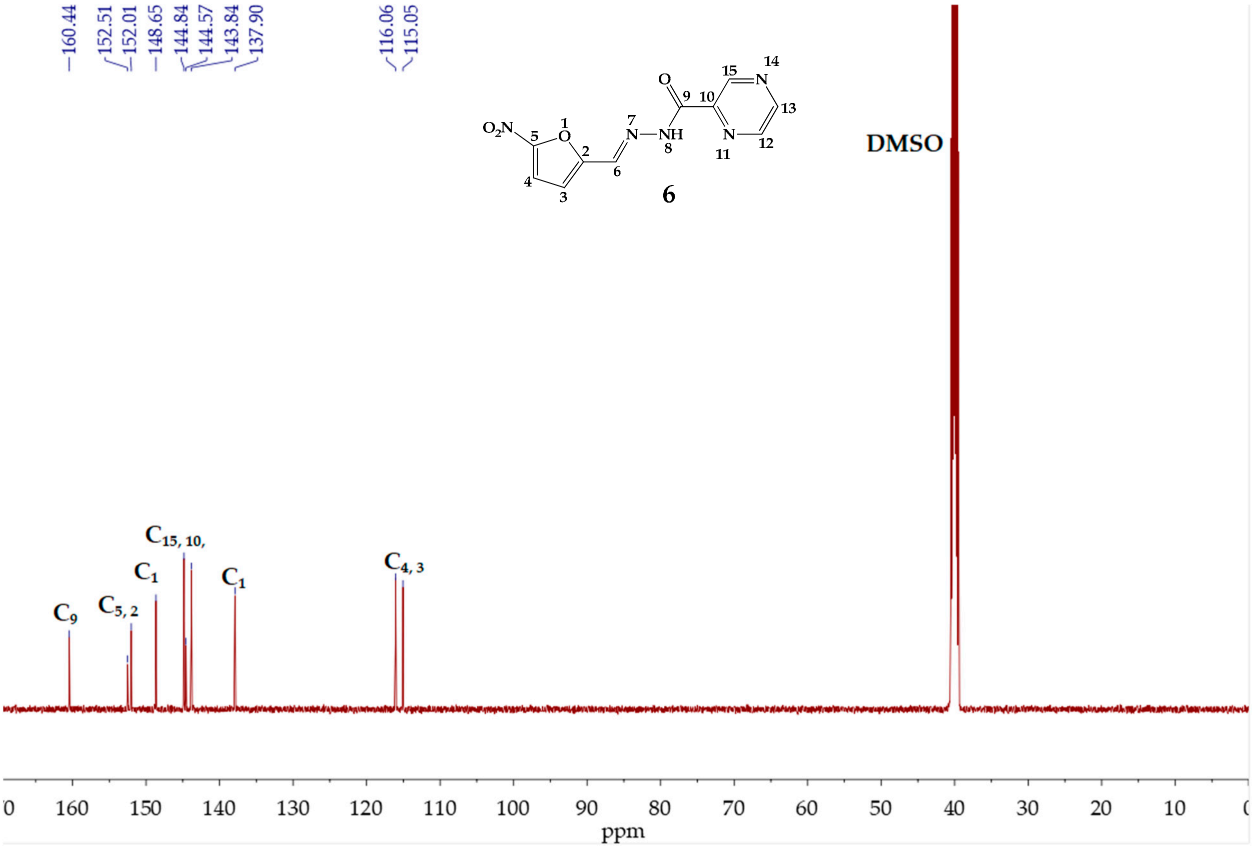
160.44
152.51
152.01
148.65
144.84
144.57
143.84
137.90
116.06
115.05
DMSO
6
C9
C5, 2
C1
C15, 10,
C1
C4, 3
ppm

b

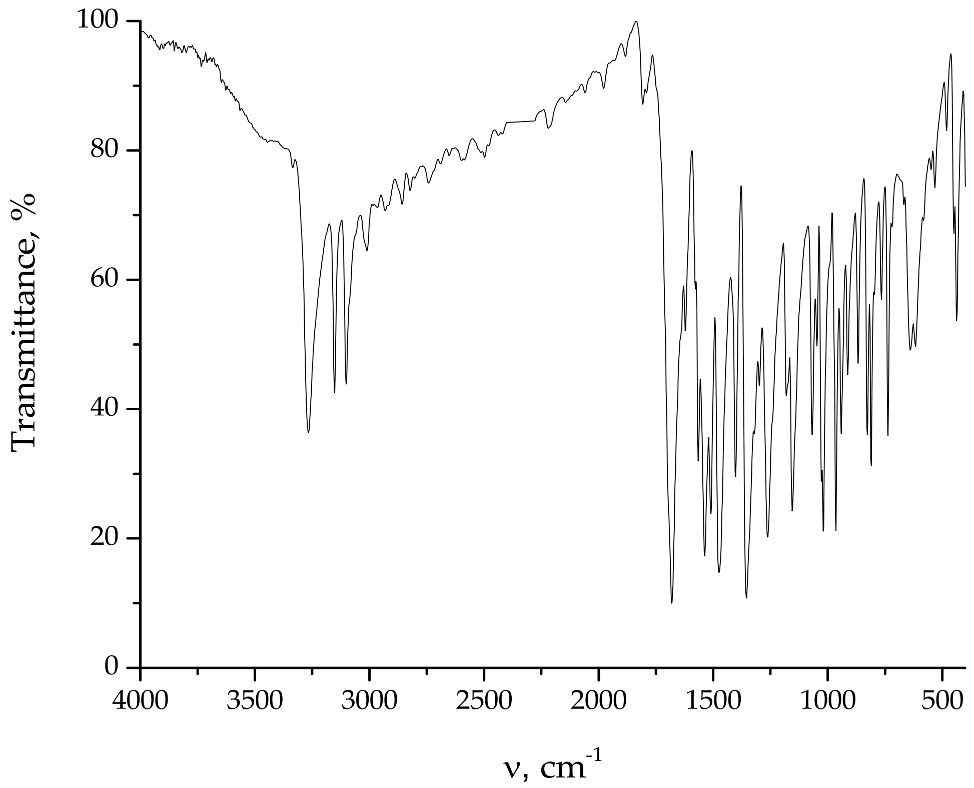

c

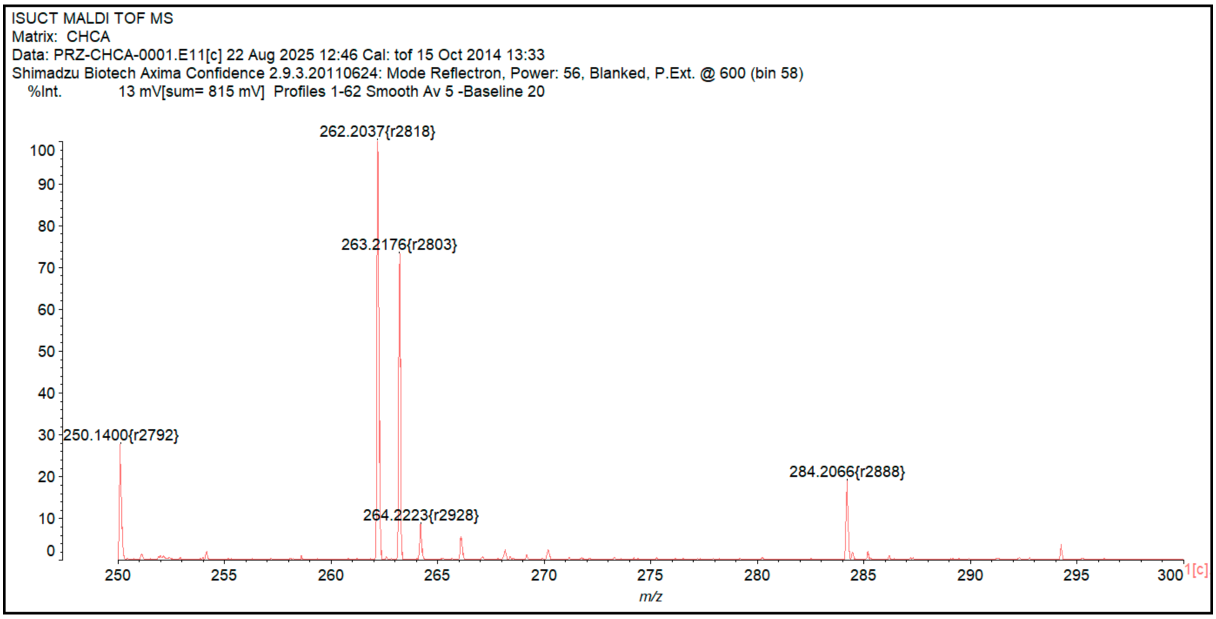

d

Figure S6. ^{1}H (a), ^{13}C (b) NMR, IR (c) and mass (d) spectra of N'-((5-nitrofuran-2-yl)methylene)pyrazine-2-carbohydrazide **6**

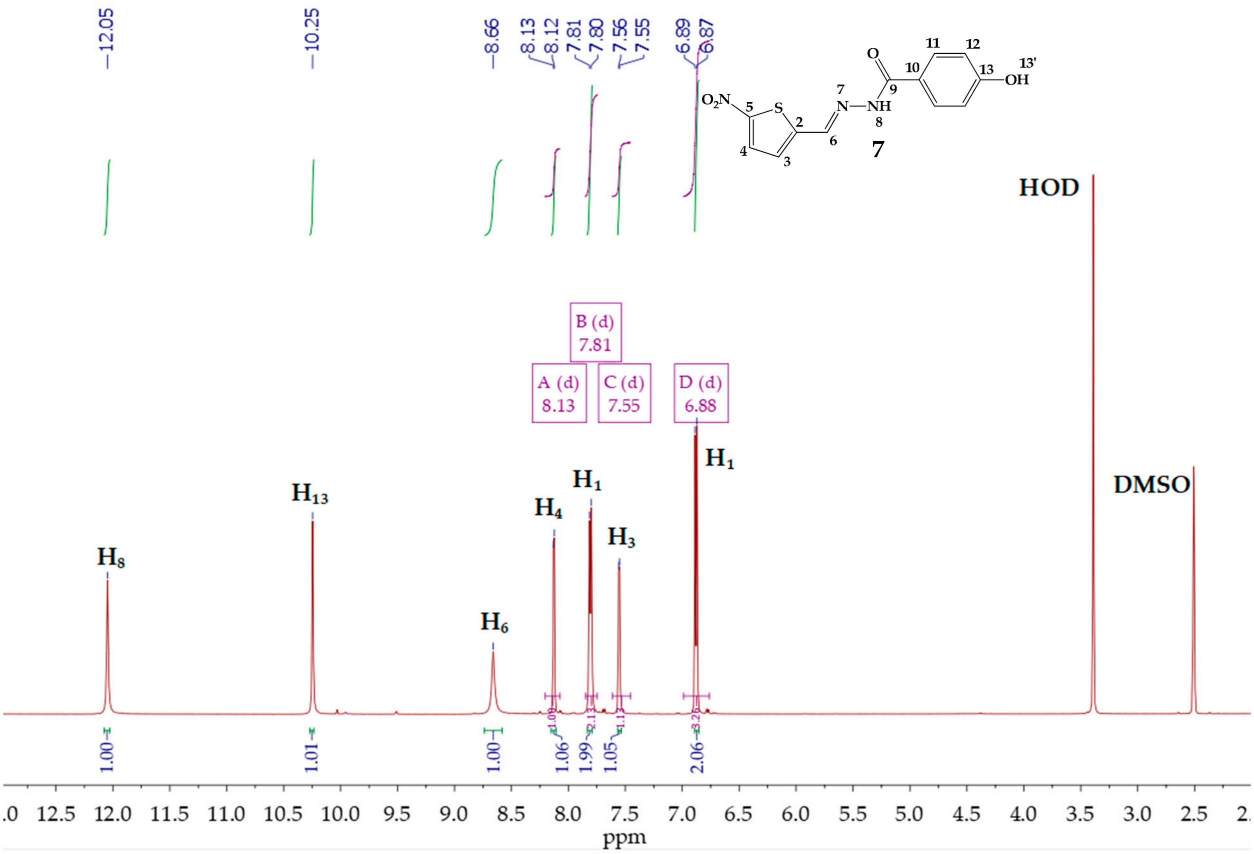
12.05
10.25
8.66
8.13
8.12
7.81
7.80
7.56
7.55
6.89
6.87
7
HOD
DMSO
B (d) 7.81
A (d) 8.13
C (d) 7.55
D (d) 6.88
H8
H13
H6
H4
H1
H3
H1
1.00
1.01
1.00
1.06
1.99
1.05
2.06
ppm

a

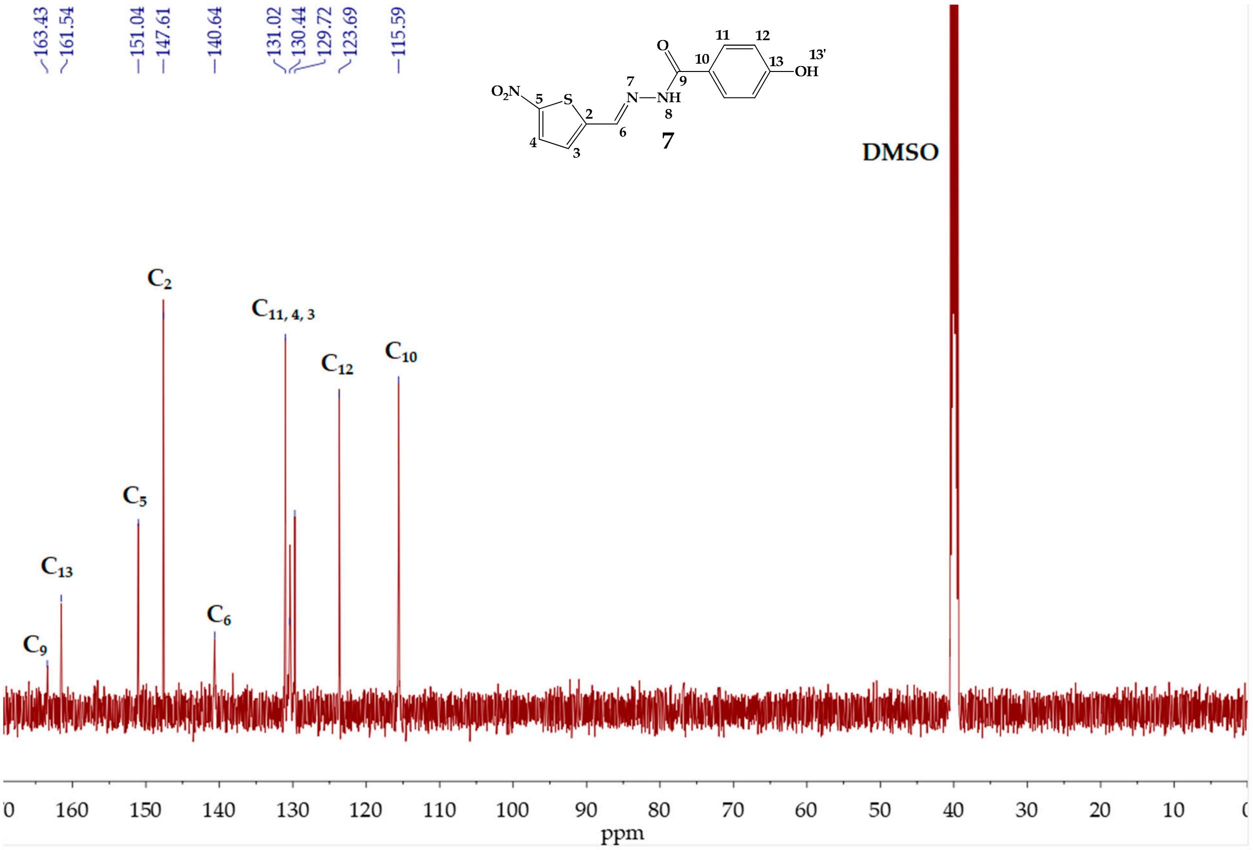
163.43
161.54
151.04
147.61
140.64
131.02
130.44
129.72
123.69
115.59
7
DMSO
C2
C11, 4, 3
C12
C10
C5
C13
C6
C9
ppm

b

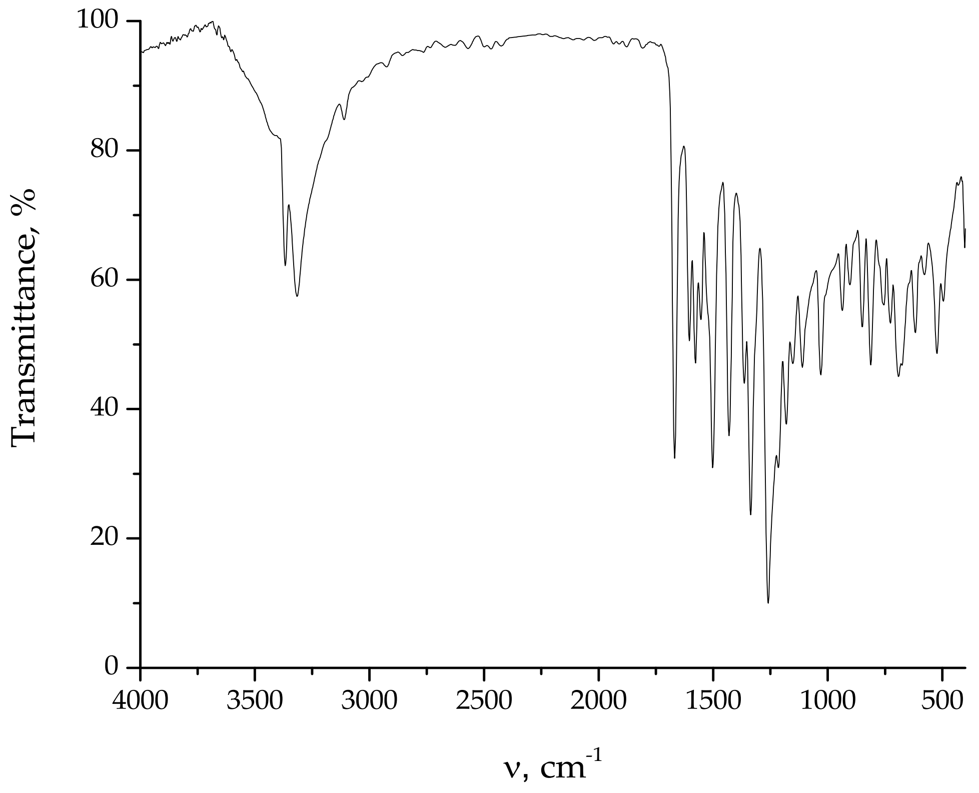

c

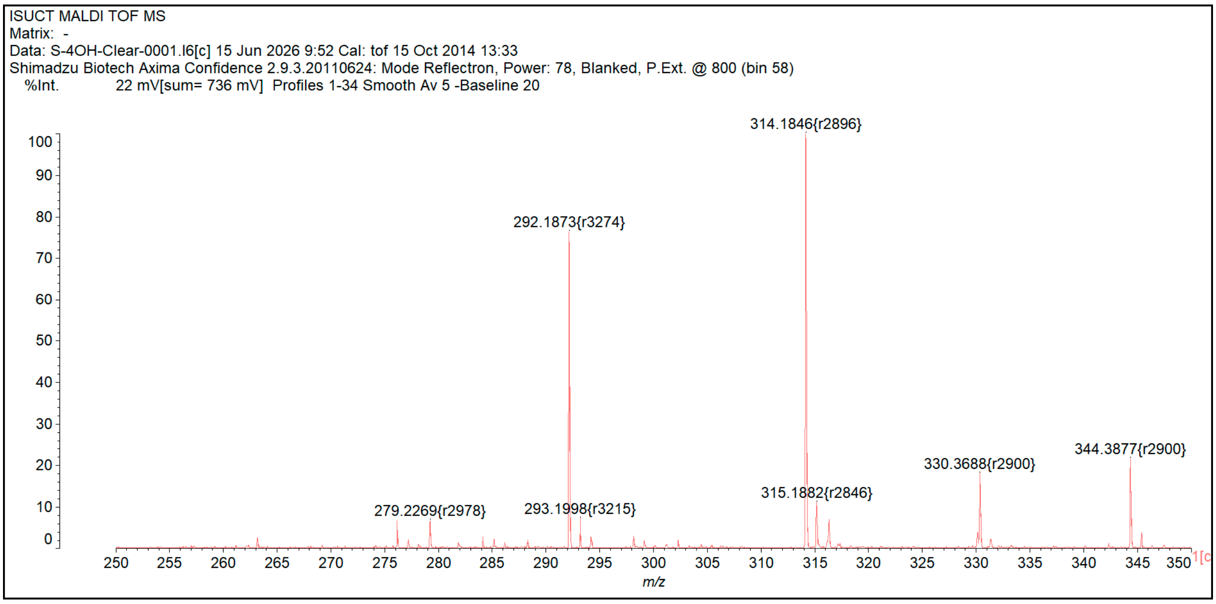

d

Figure S7. ^{1}H (a), ^{13}C (b) NMR, IR (c) and mass (d) spectra of 4-hydroxy-N'-((5-nitrothiophen-2-yl)methylene)benzohydrazide **7**

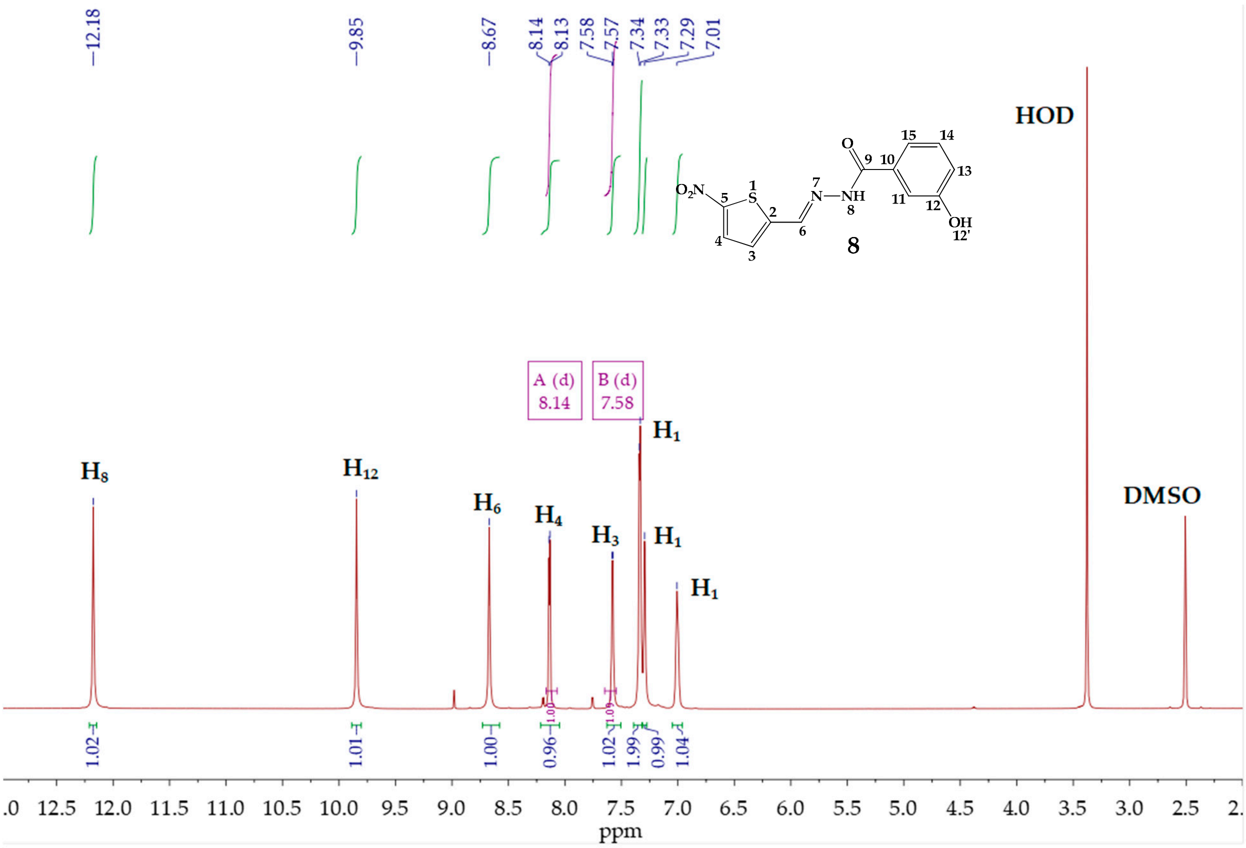

12.18
9.85
8.67
8.14
8.13
7.58
7.57
7.34
7.33
7.29
7.01
HOD
DMSO
A (d) 8.14
B (d) 7.58
H8
H12
H6
H4
H3
H1
H1
H1
1.02
1.01
1.00
0.96
1.00
1.02
1.09
1.99
0.99
1.04
ppm
O2N
8

a

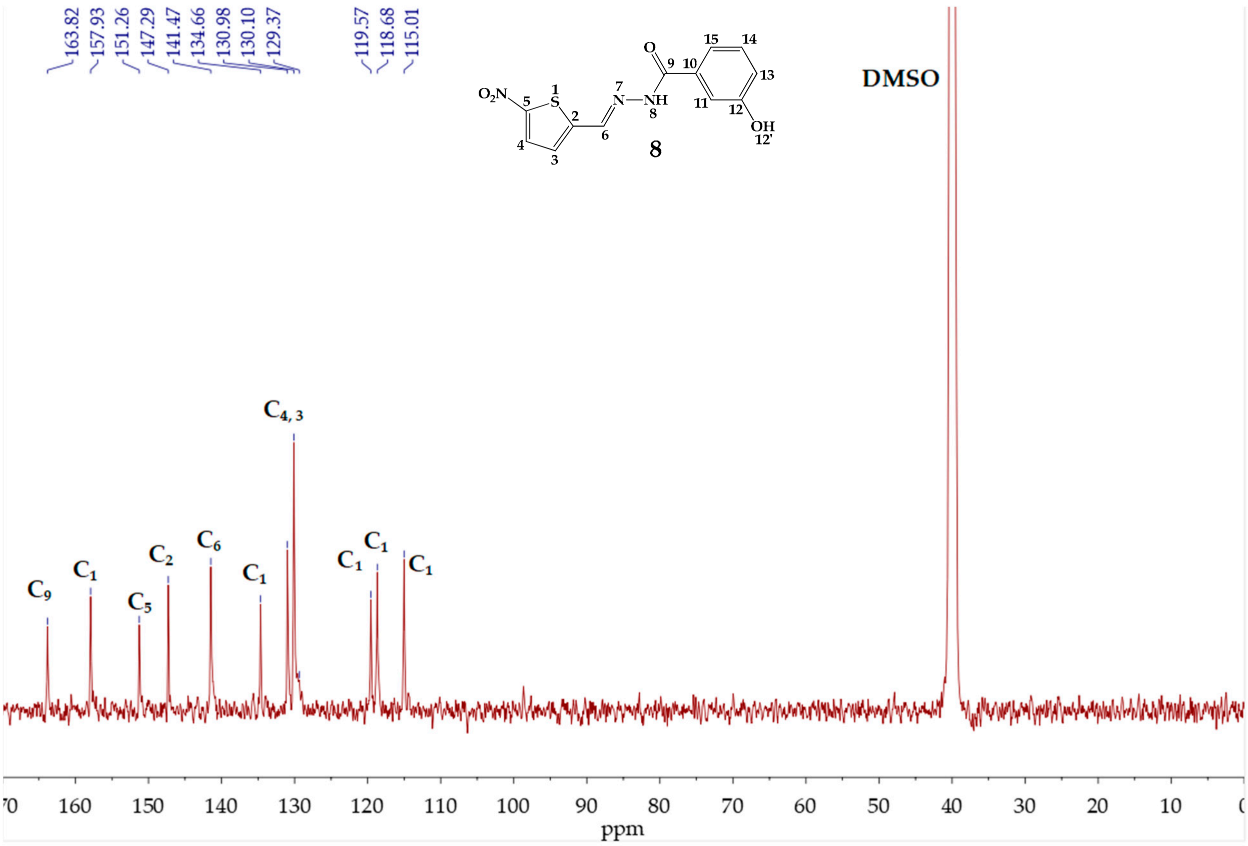

163.82
157.93
151.26
147.29
141.47
134.66
130.98
130.10
129.37
119.57
118.68
115.01
DMSO
C4, 3
C9
C1
C5
C2
C6
C1
C1
C1
C1
ppm
O2N
8

b

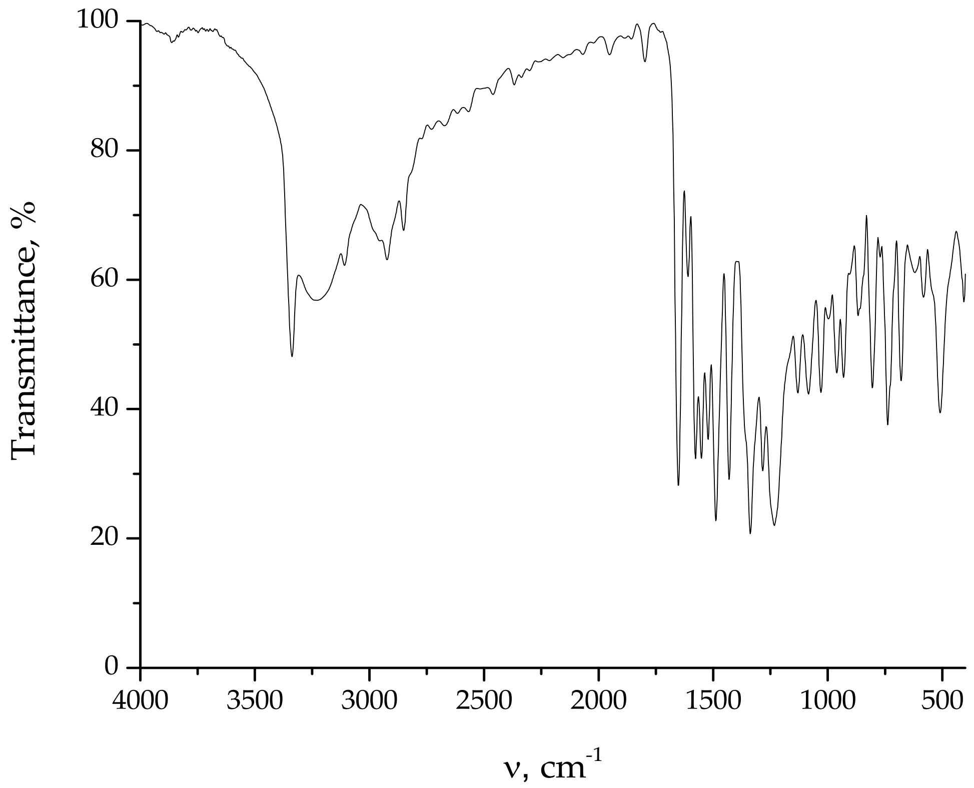

c

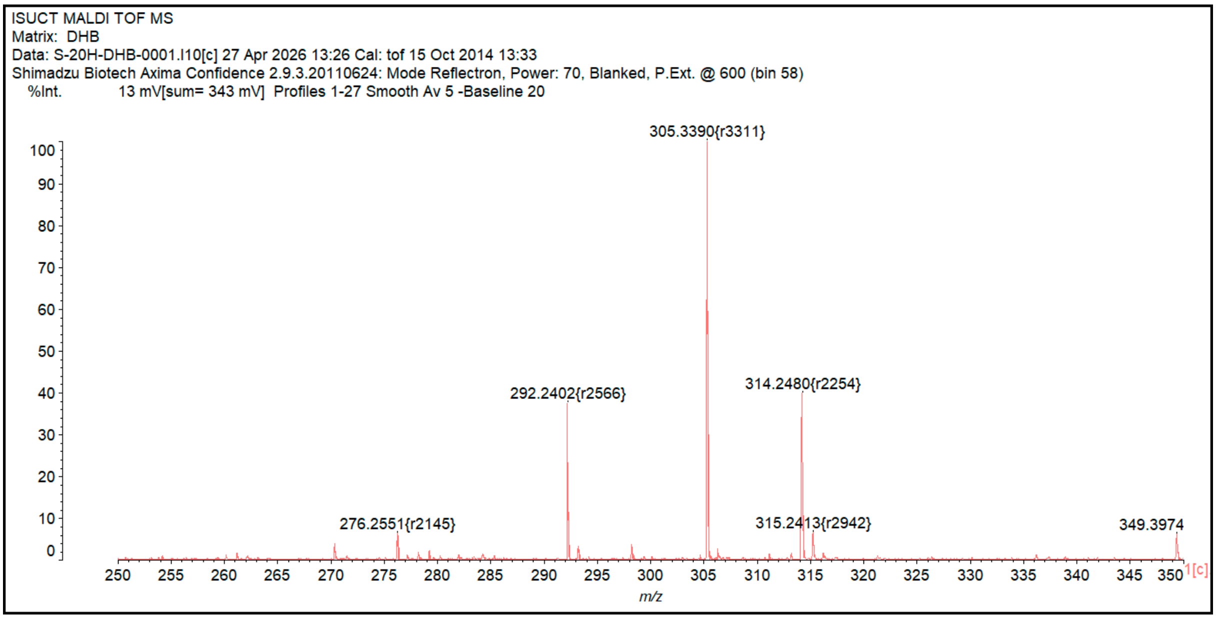

d

Figure S8. ^{1}H (a), ^{13}C (b) NMR, IR (c) and mass (d) spectra of 3-hydroxy-N'-((5-nitrothiophen-2-yl)methylene)benzohydrazide **8**

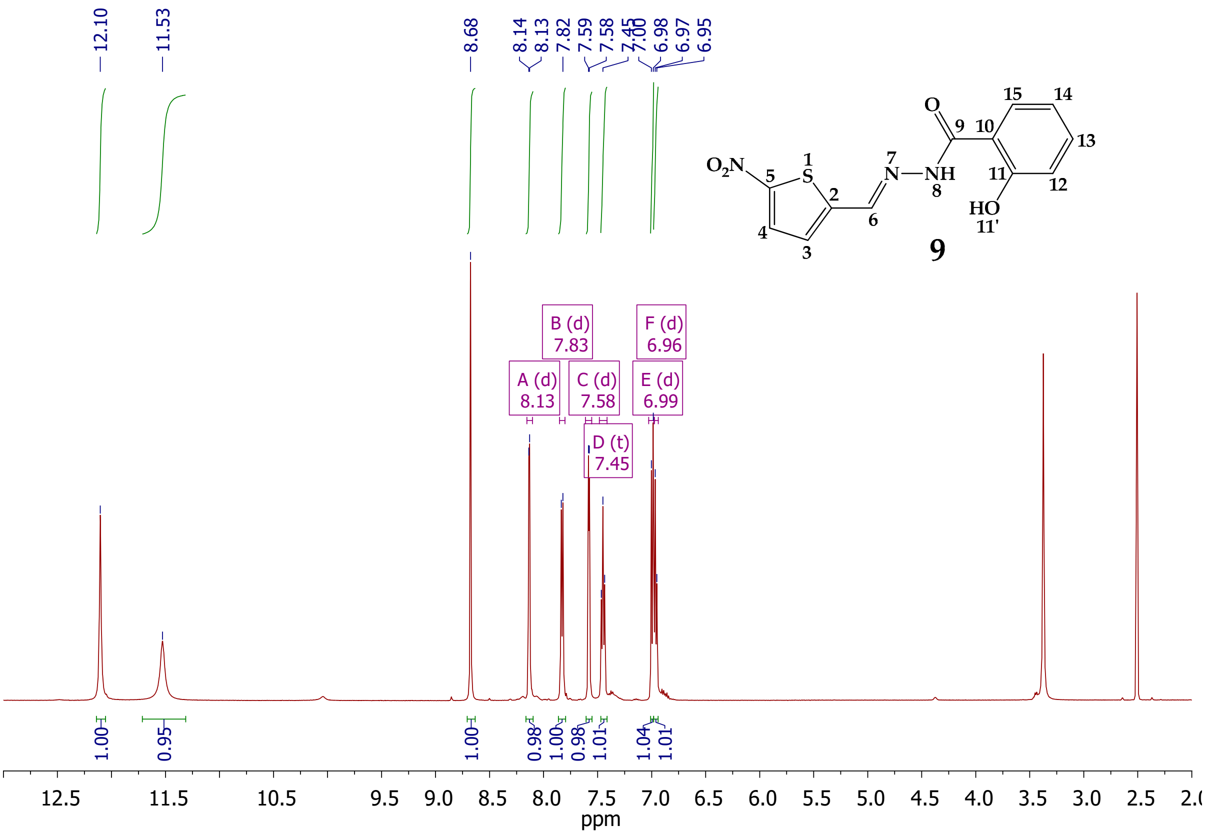
12.10
11.53
8.68
8.14
8.13
7.82
7.59
7.58
7.45
7.00
6.98
6.97
6.95
A (d) 8.13
B (d) 7.83
C (d) 7.58
D (t) 7.45
E (d) 6.99
F (d) 6.96
1.00
0.95
1.00
0.98
1.00
0.98
1.01
1.04
1.01
ppm
9

a

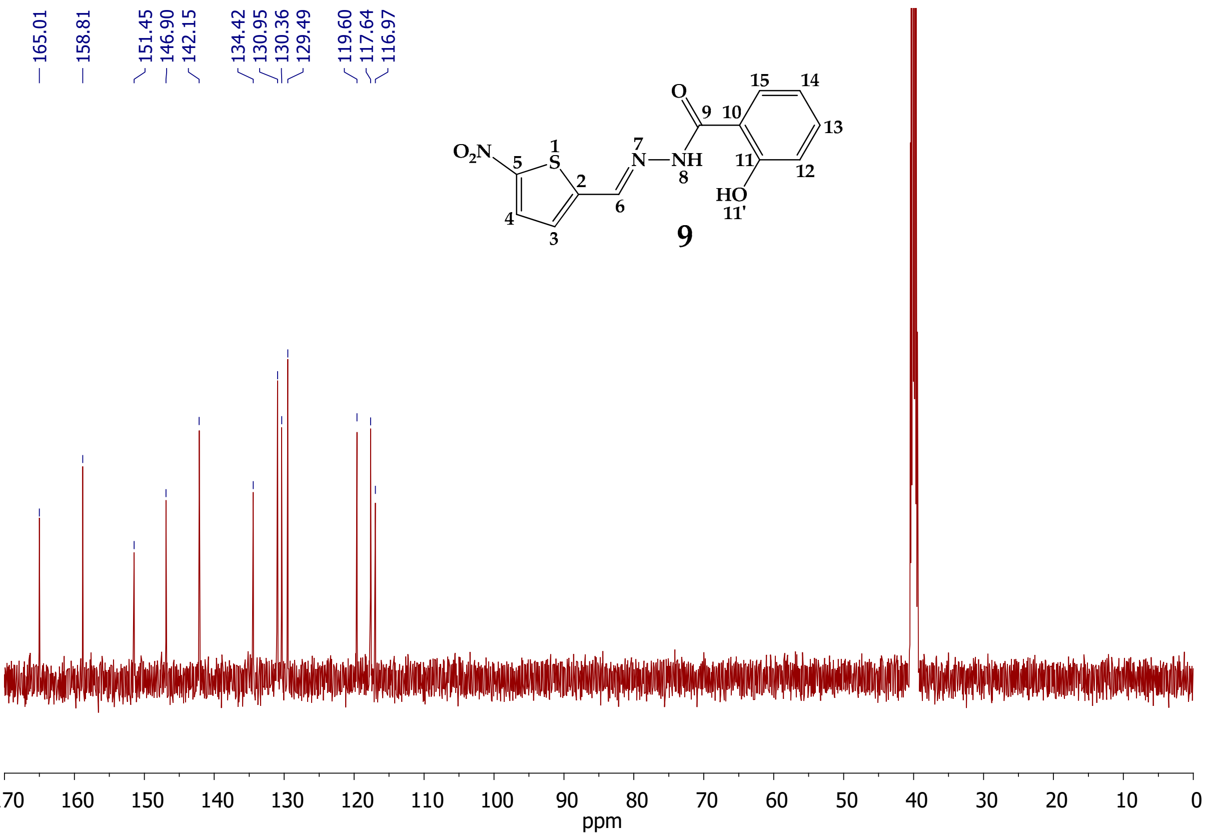
165.01
158.81
151.45
146.90
142.15
134.42
130.95
130.36
129.49
119.60
117.64
116.97
ppm
9

b

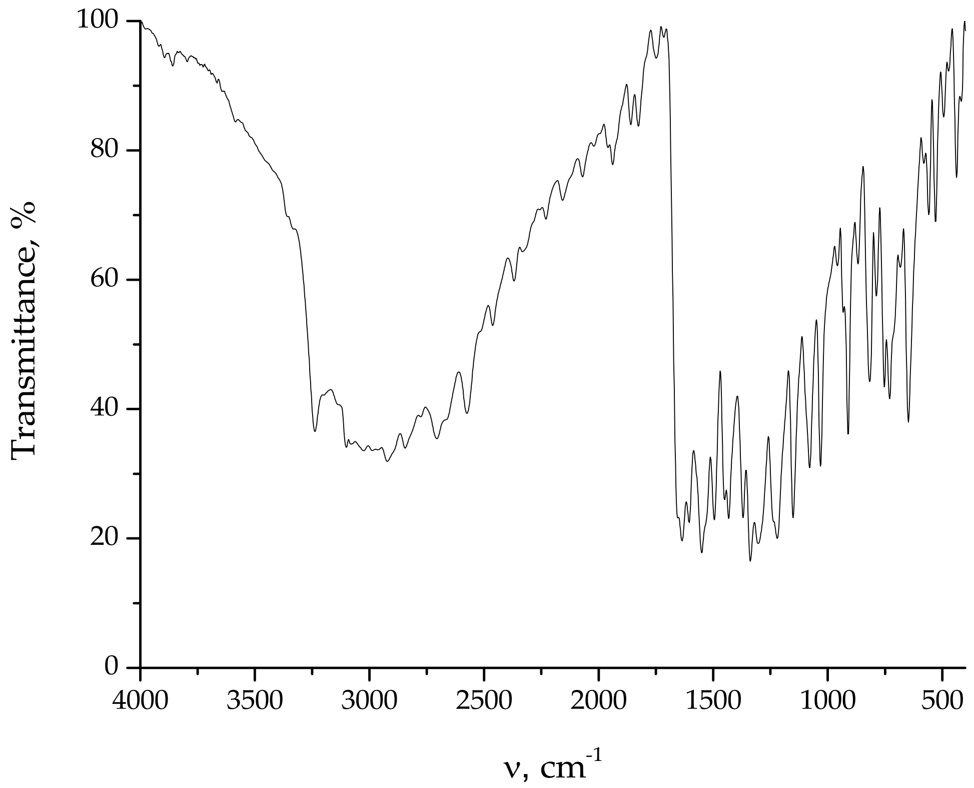

c

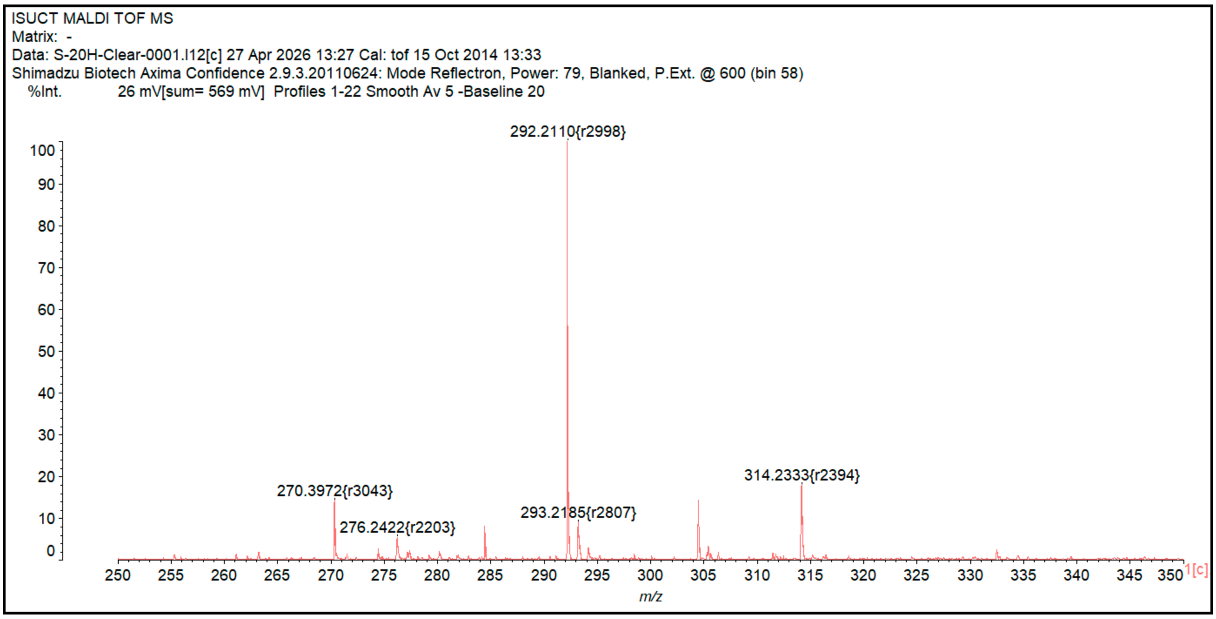

d

Figure S9. ^{1}H (a), ^{13}C (b) NMR, IR (c) and mass (d) spectra of 2-hydroxy-N'-((5-nitrothiophen-2-yl)methylene)benzohydrazide **9**

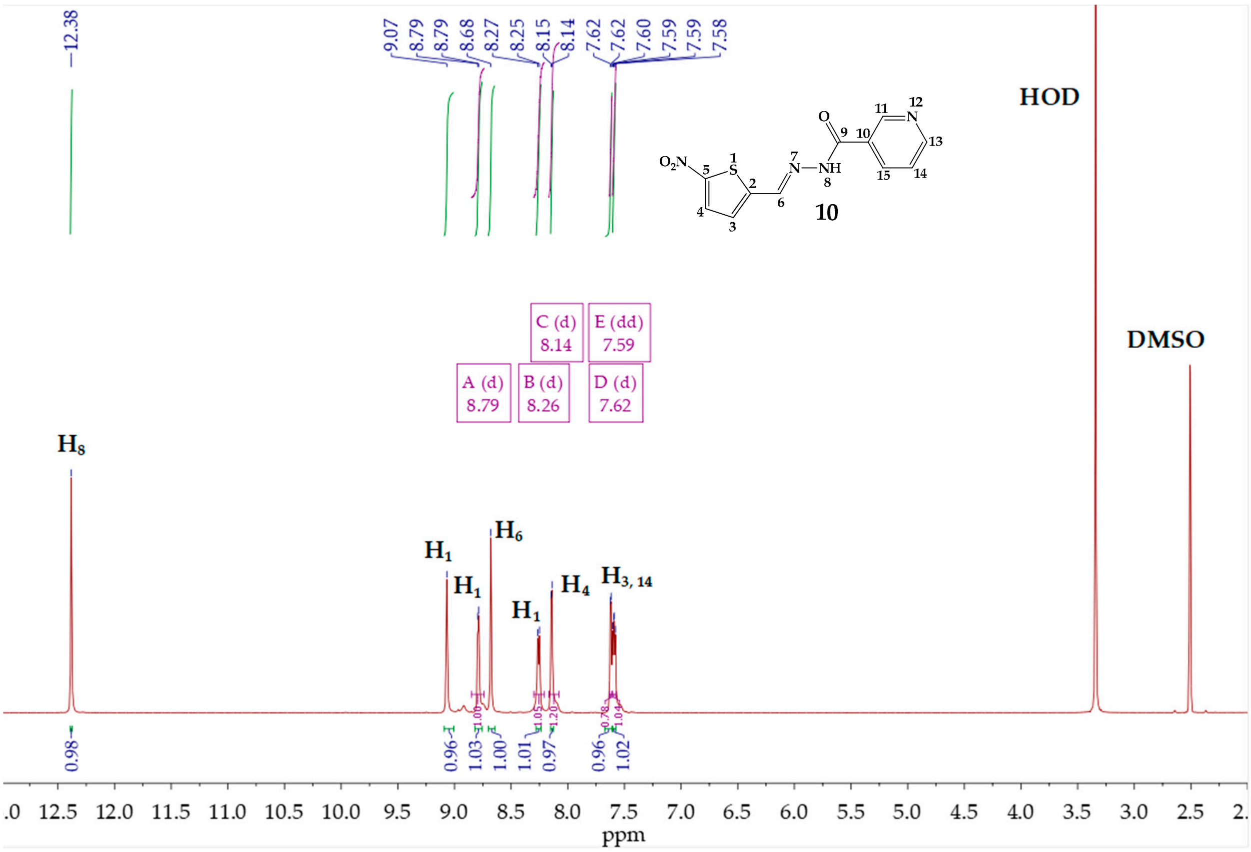

12.38
9.07
8.79
8.79
8.68
8.27
8.25
8.15
8.14
7.62
7.62
7.60
7.59
7.59
7.58
HOD
DMSO
10
C (d) 8.14
E (dd) 7.59
A (d) 8.79
B (d) 8.26
D (d) 7.62
H8
H1
H6
H1
H4
H1
H3, 14
0.98
0.96
1.03
1.00
1.01
0.97
0.96
1.02
ppm

a

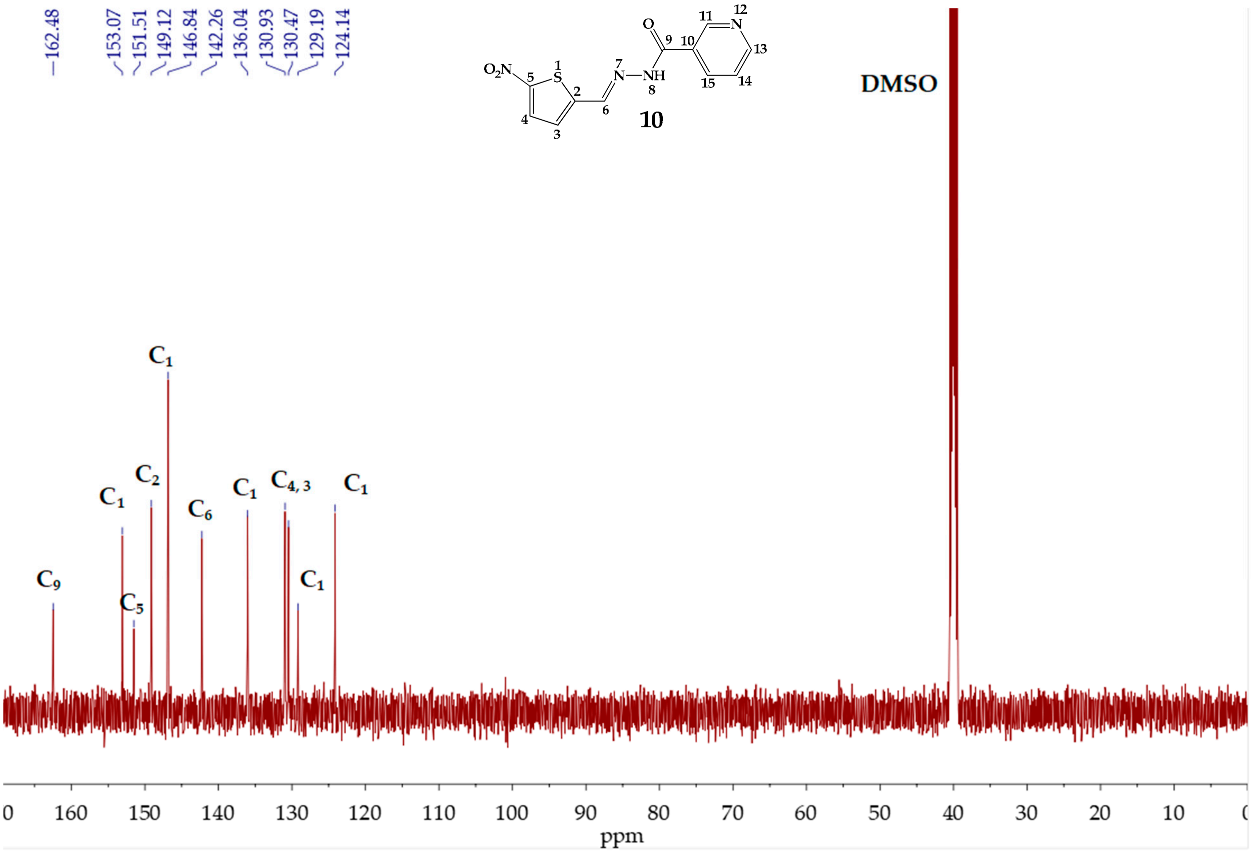

162.48
153.07
151.51
149.12
146.84
142.26
136.04
130.93
130.47
129.19
124.14
10
DMSO
C1
C2
C1
C6
C1
C4, 3
C1
C9
C5
C1
ppm

b

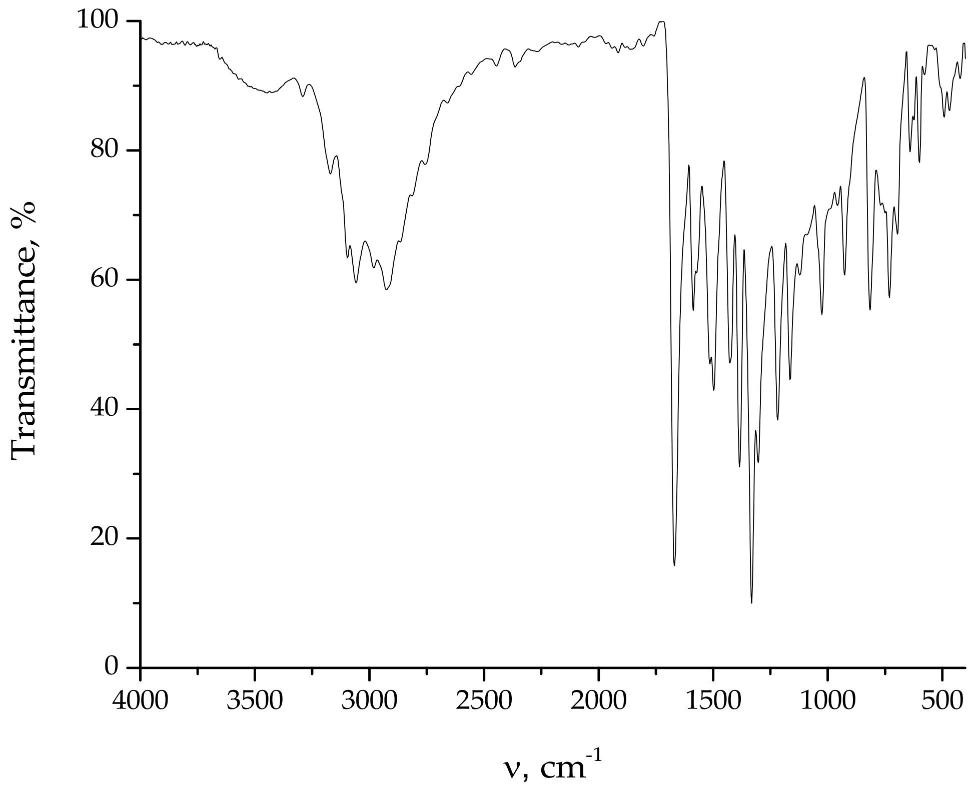

c

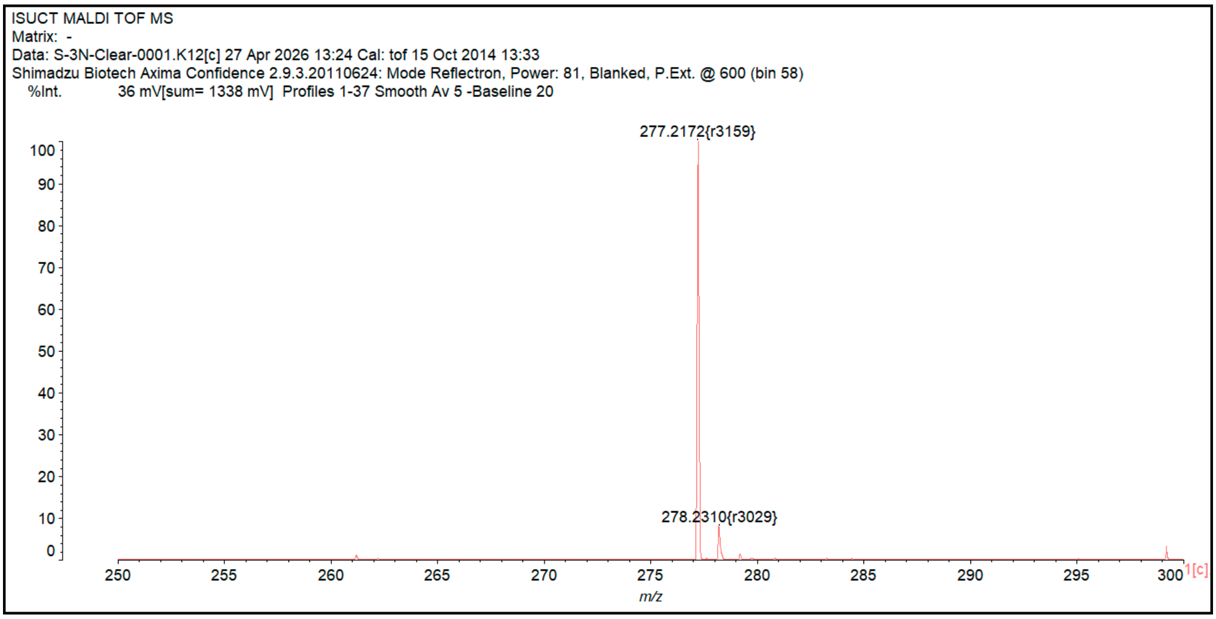

d

Figure S10. ^{1}H (a), ^{13}C (b) NMR, IR (c) and mass (d) spectra of N'-((5-nitrothiophen-2-yl)methylene)pyridine-3-carbohydrazide **10**

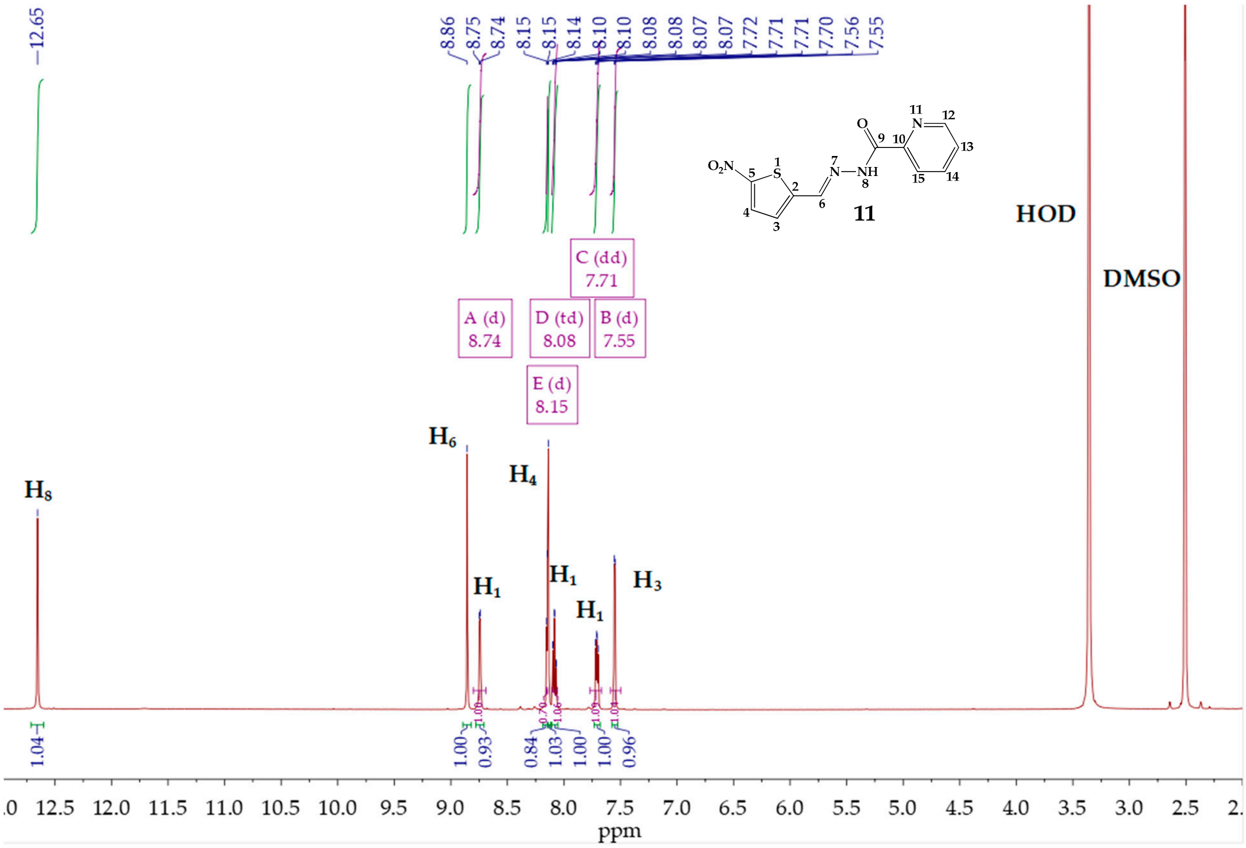

a

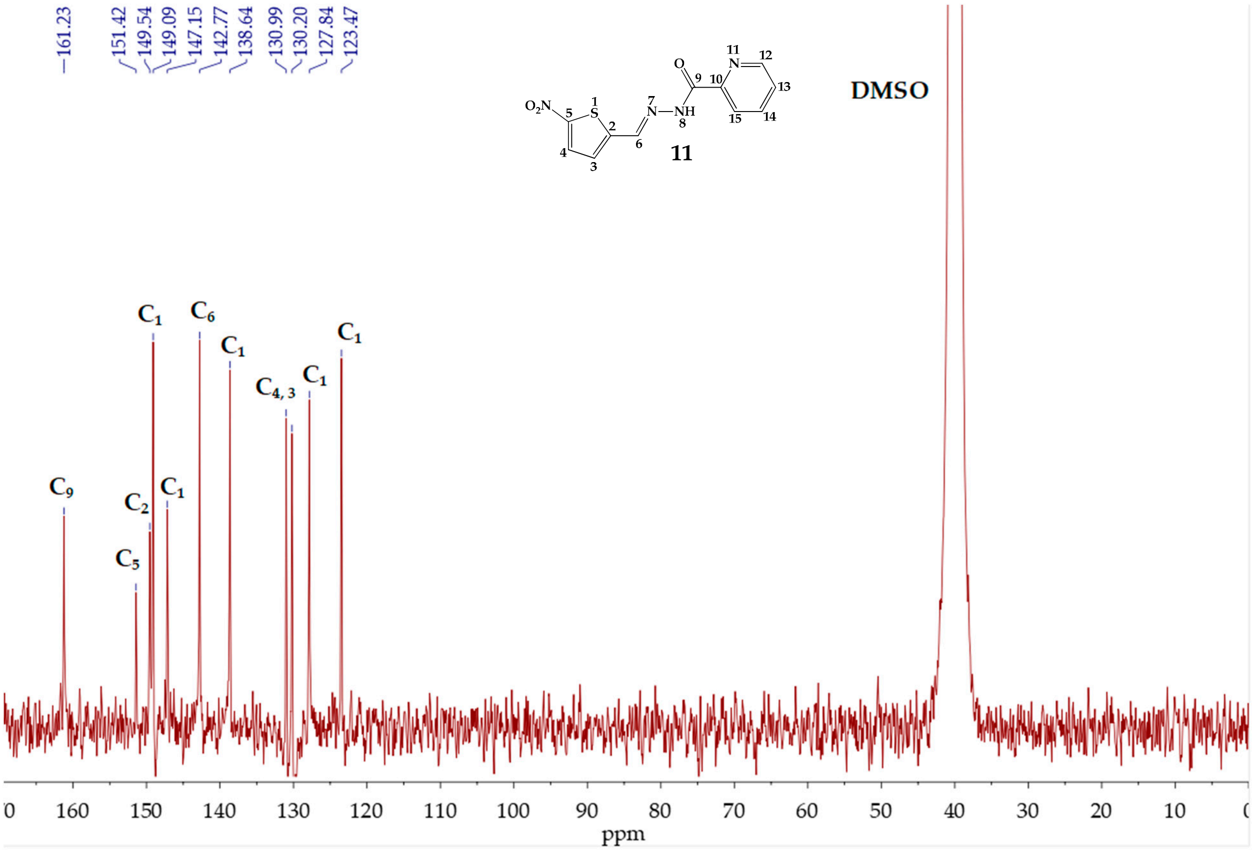

b

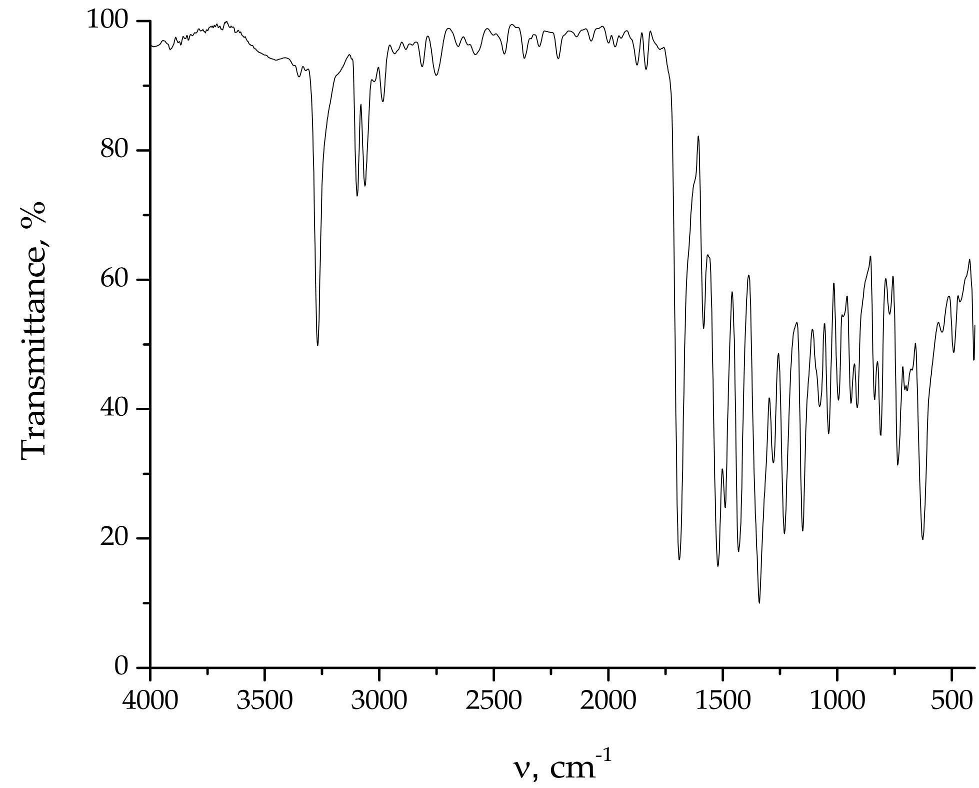

c

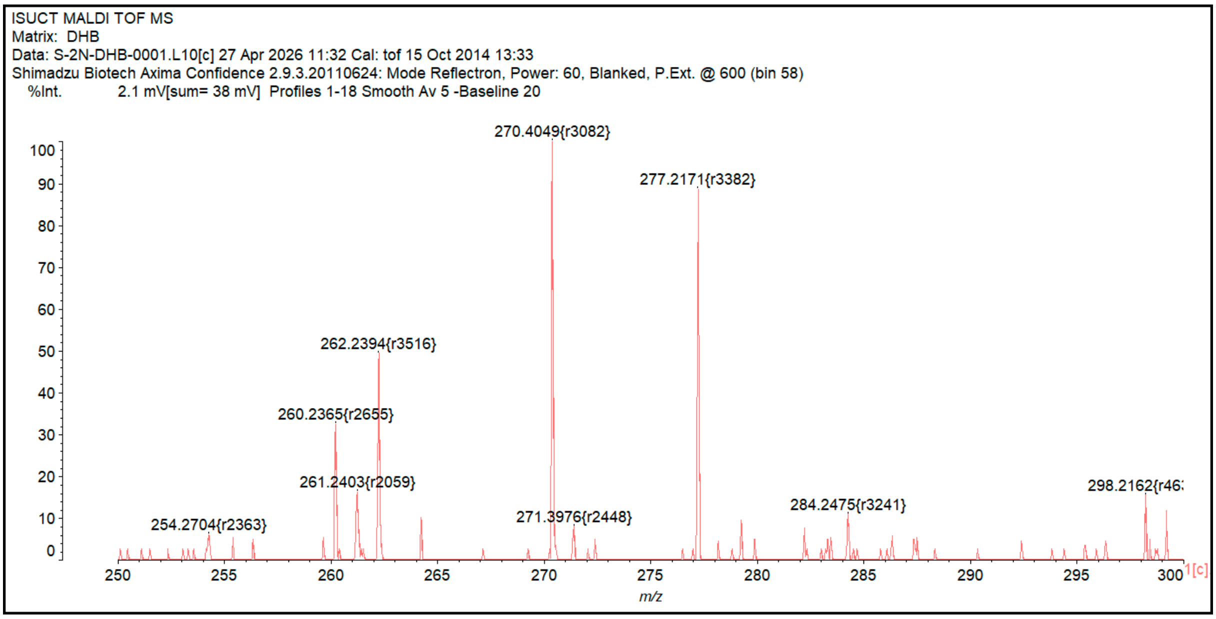

d

Figure S11. ^{1}H (a), ^{13}C (b) NMR, IR (c) and mass (d) spectra of N'-((5-nitrothiophen-2-yl)methylene)pyridine-2-carbohydrazide **11**

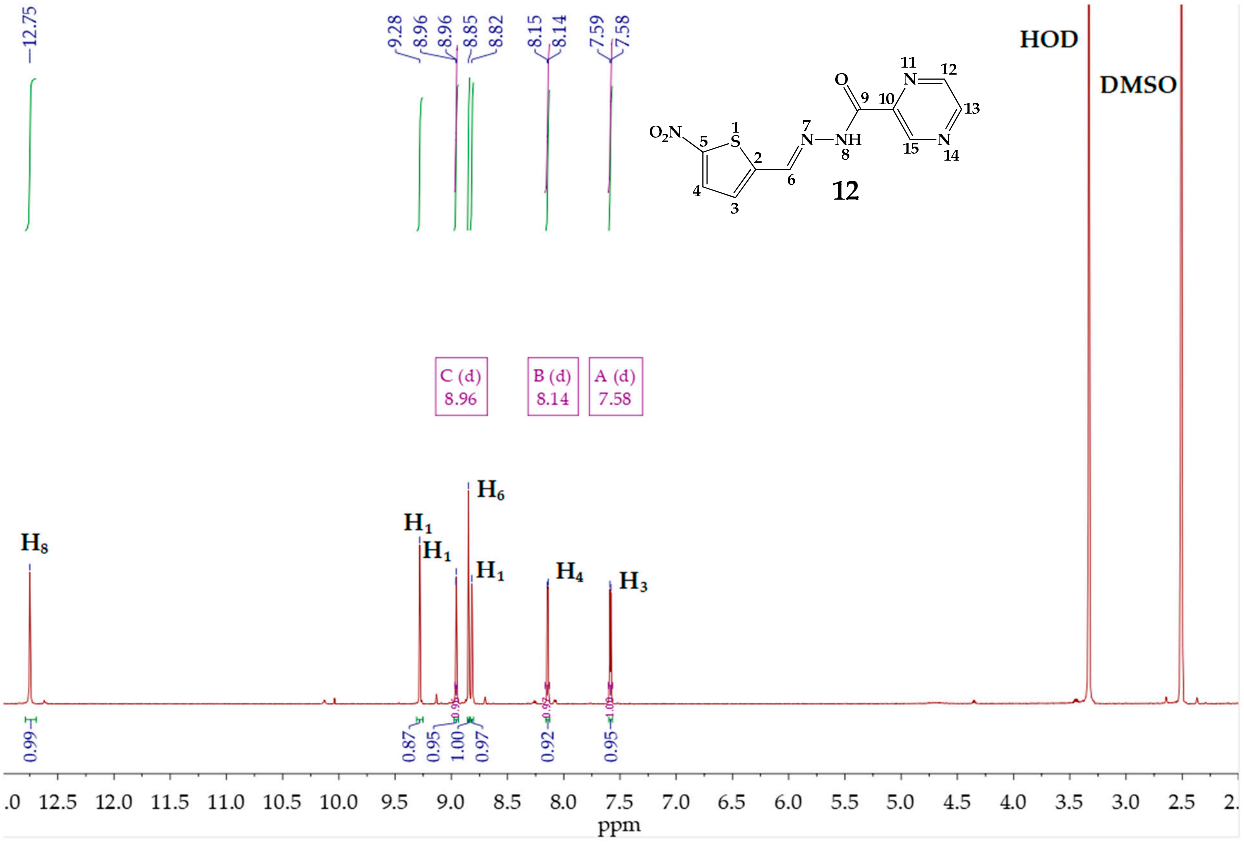

12.75
9.28
8.96
8.96
8.85
8.82
8.15
8.14
7.59
7.58
HOD
DMSO
12
C (d) 8.96
B (d) 8.14
A (d) 7.58
ppm

a

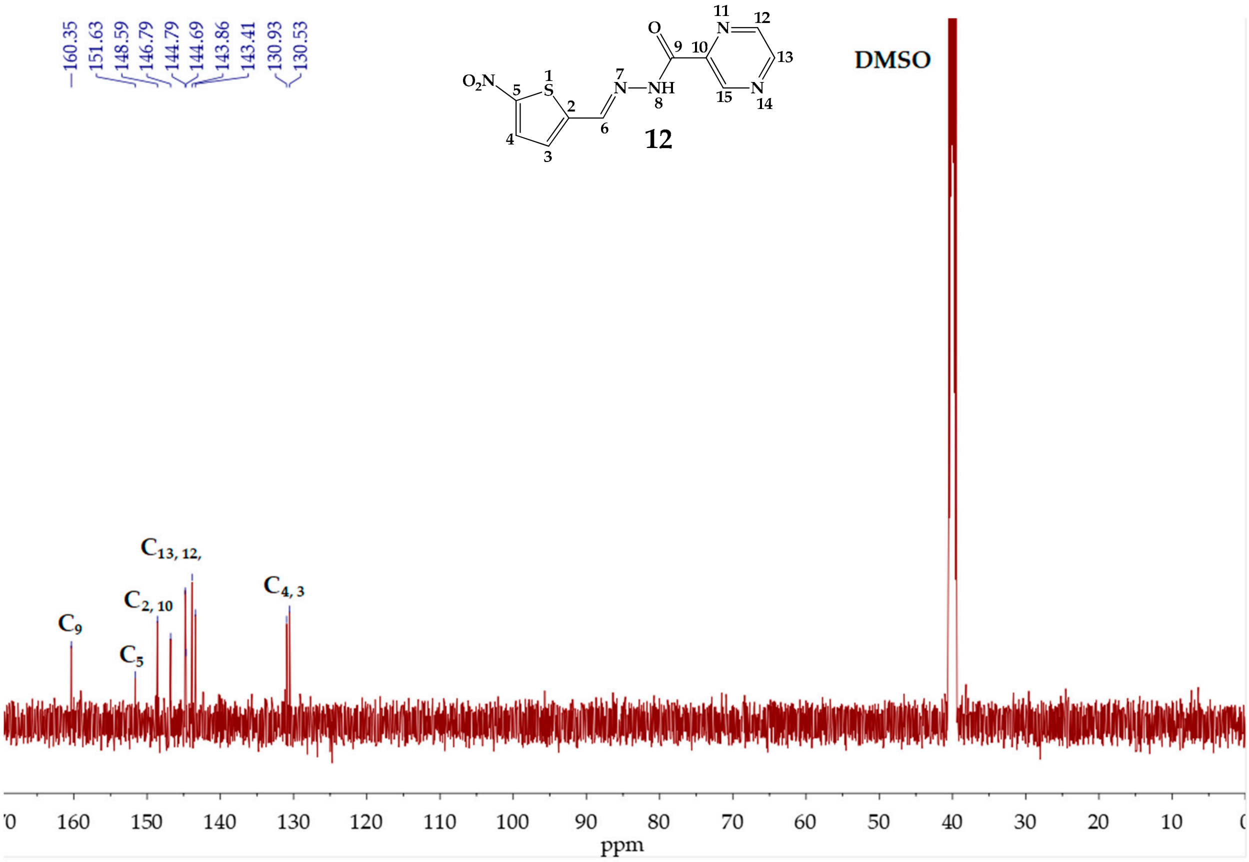

160.35
151.63
148.59
146.79
144.79
144.69
143.86
143.41
130.93
130.53
DMSO
12
ppm

b

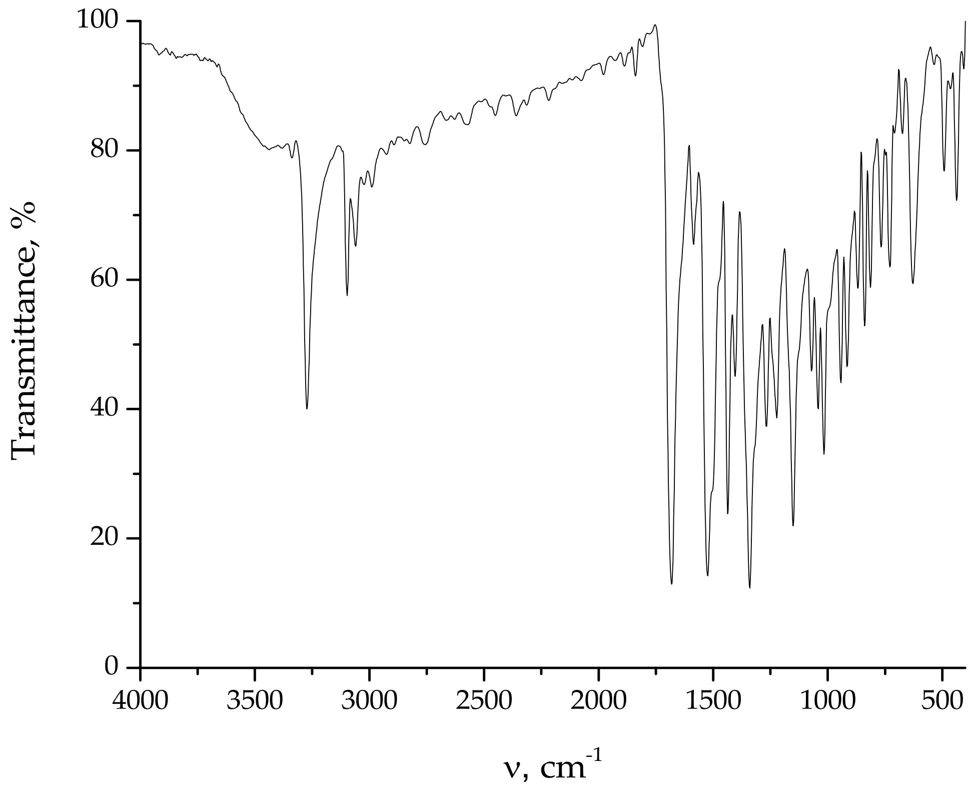

c

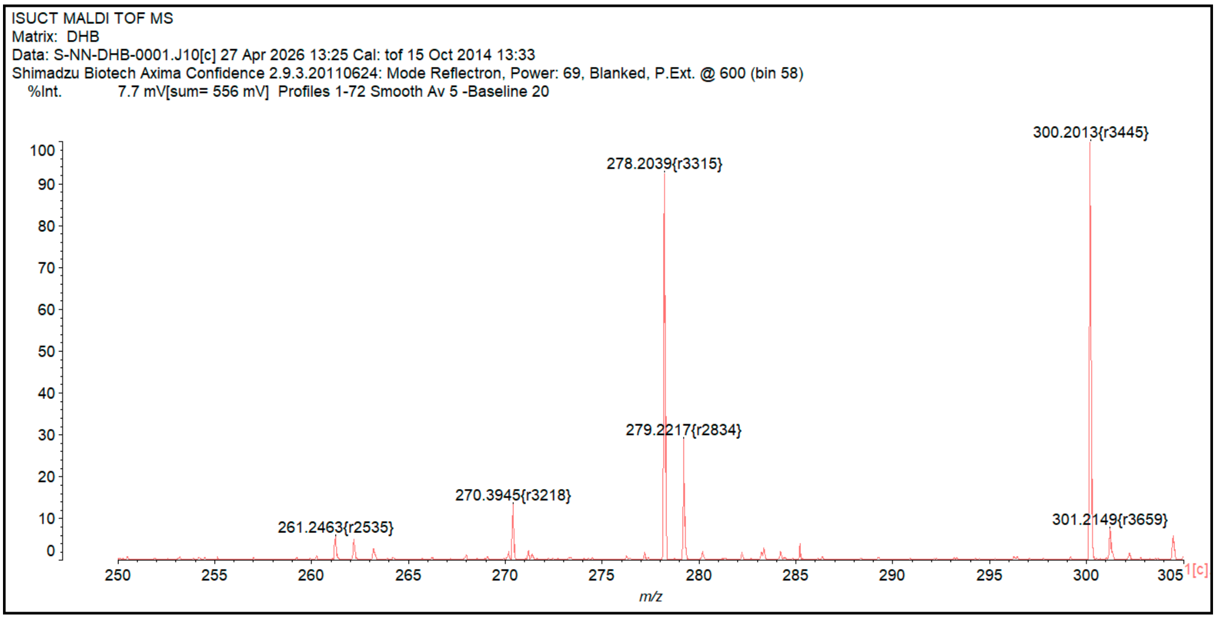

d

Figure S12. ^{1}H (a), ^{13}C (b) NMR, IR (c) and mass (d) spectra of N'-((5-nitrothiophen-2-yl)methylene)pyrazine-2-carbohydrazide **12**

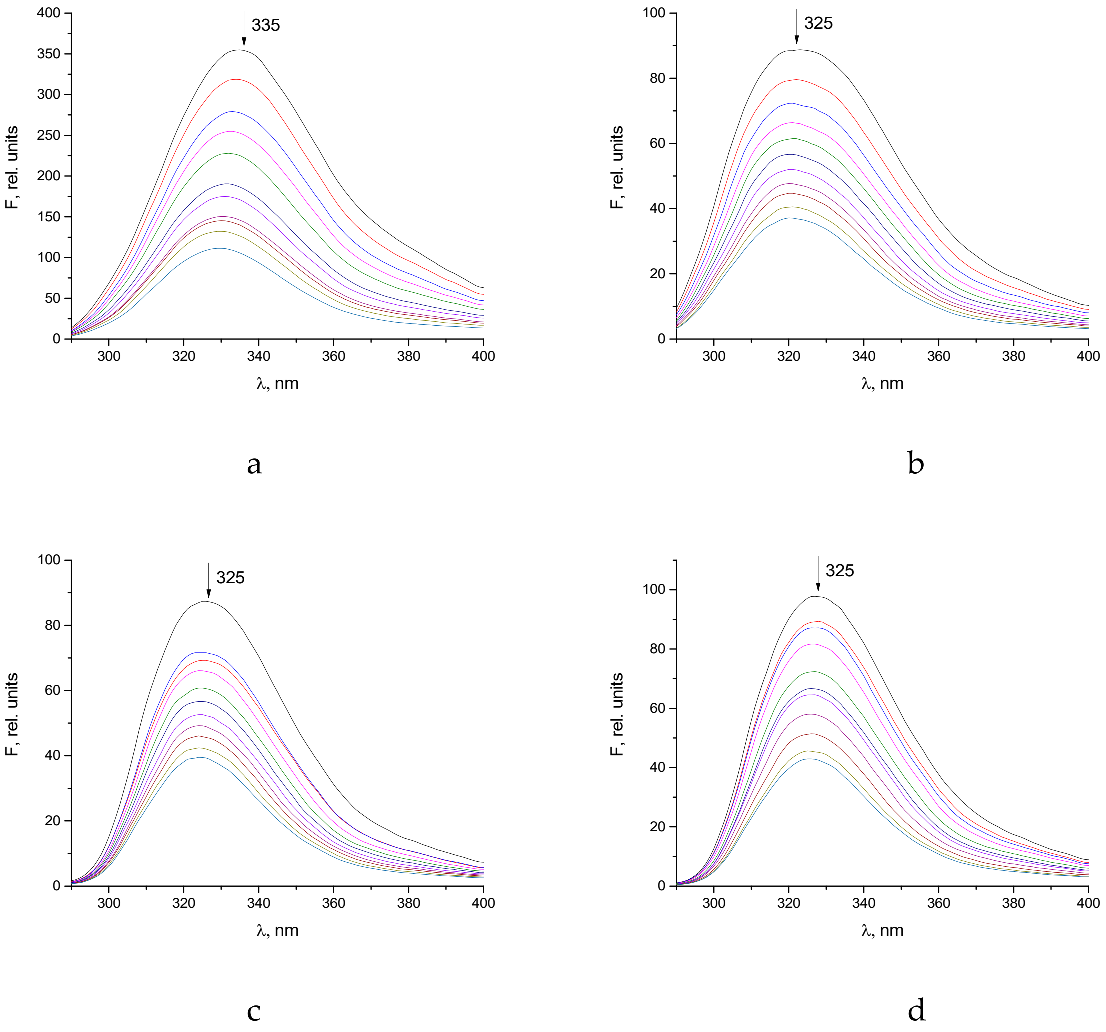

Figure S13. Inner filter-corrected fluorescent spectra of bovine serum albumin (a), human serum albumin (b), bovine γ-globulin (c), human γ-globulin (d) aqueous solutions (pH 6.86, T = 298.2 K, I = 0.05 M) titrated by nifuroxazide **1**

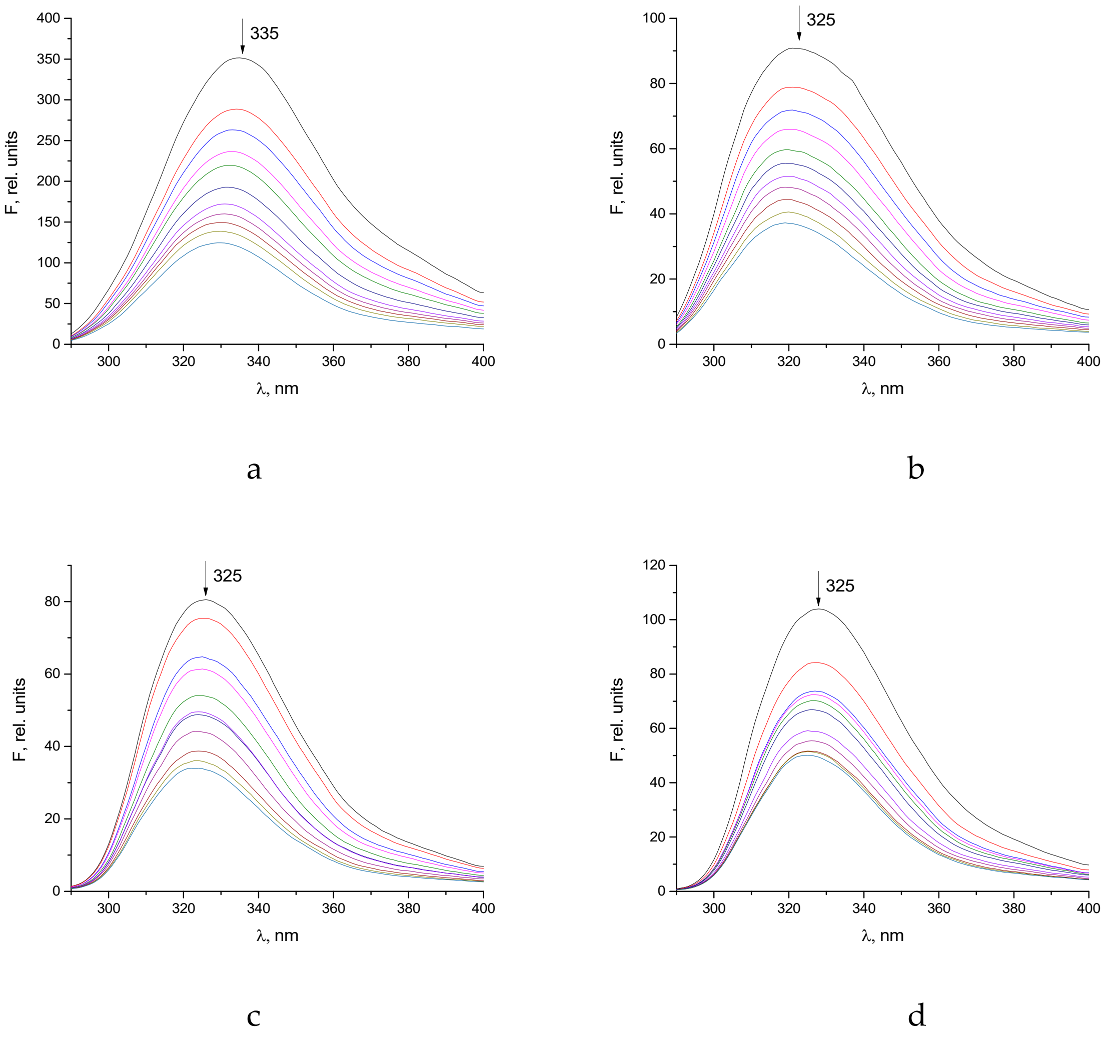

Figure S14. Inner filter-corrected fluorescent spectra of bovine serum albumin (a), human serum albumin (b), bovine γ-globulin (c), human γ-globulin (d) aqueous solutions (pH 6.86, T = 298.2 K, I = 0.05 M) titrated by hydrazone **2**

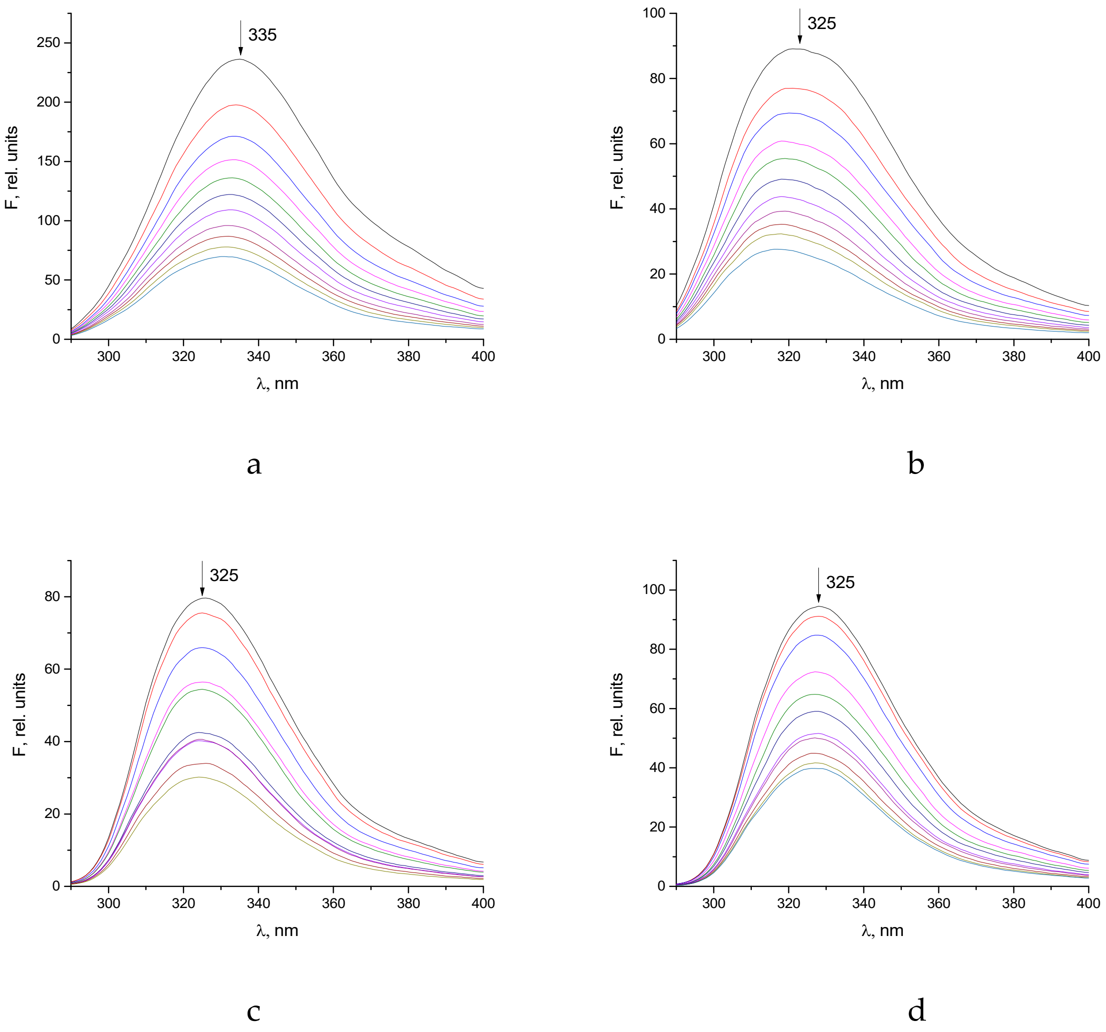

Figure S15. Inner filter-corrected fluorescent spectra of bovine serum albumin (a), human serum albumin (b), bovine γ-globulin (c), human γ-globulin (d) aqueous solutions (pH 6.86, T = 298.2 K, I = 0.05 M) titrated by hydrazone **3**

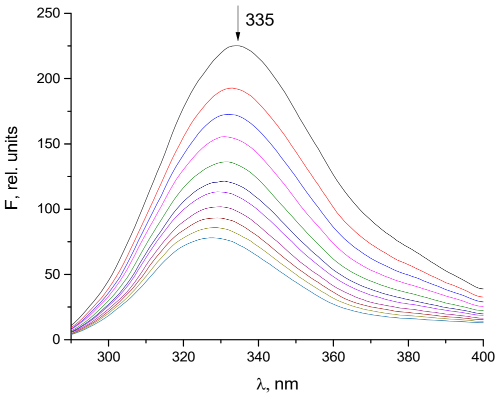

a

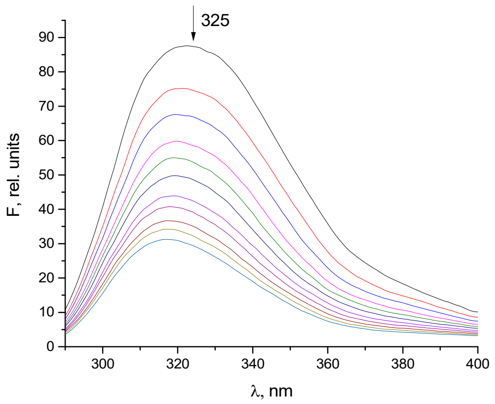

b

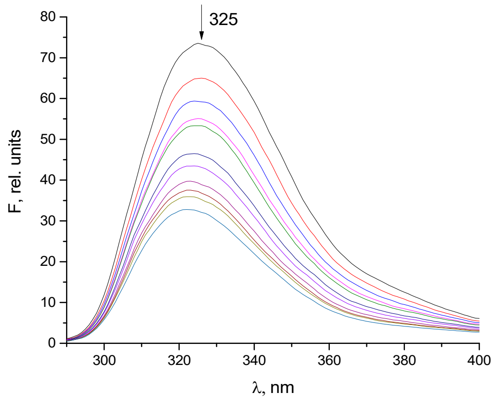

c

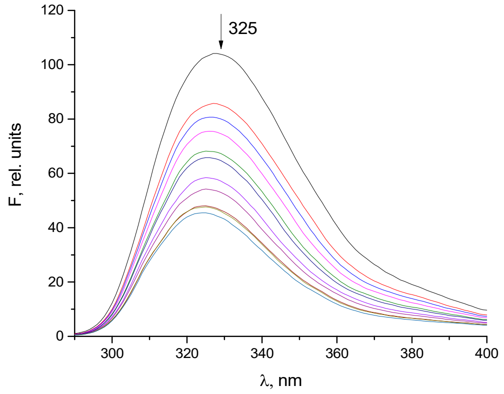

d

Figure S16. Inner filter-corrected fluorescent spectra of bovine serum albumin (a), human serum albumin (b), bovine γ-globulin (c), human γ-globulin (d) aqueous solutions (pH 6.86, T = 298.2 K, I = 0.05 M) titrated by hydrazone **4**

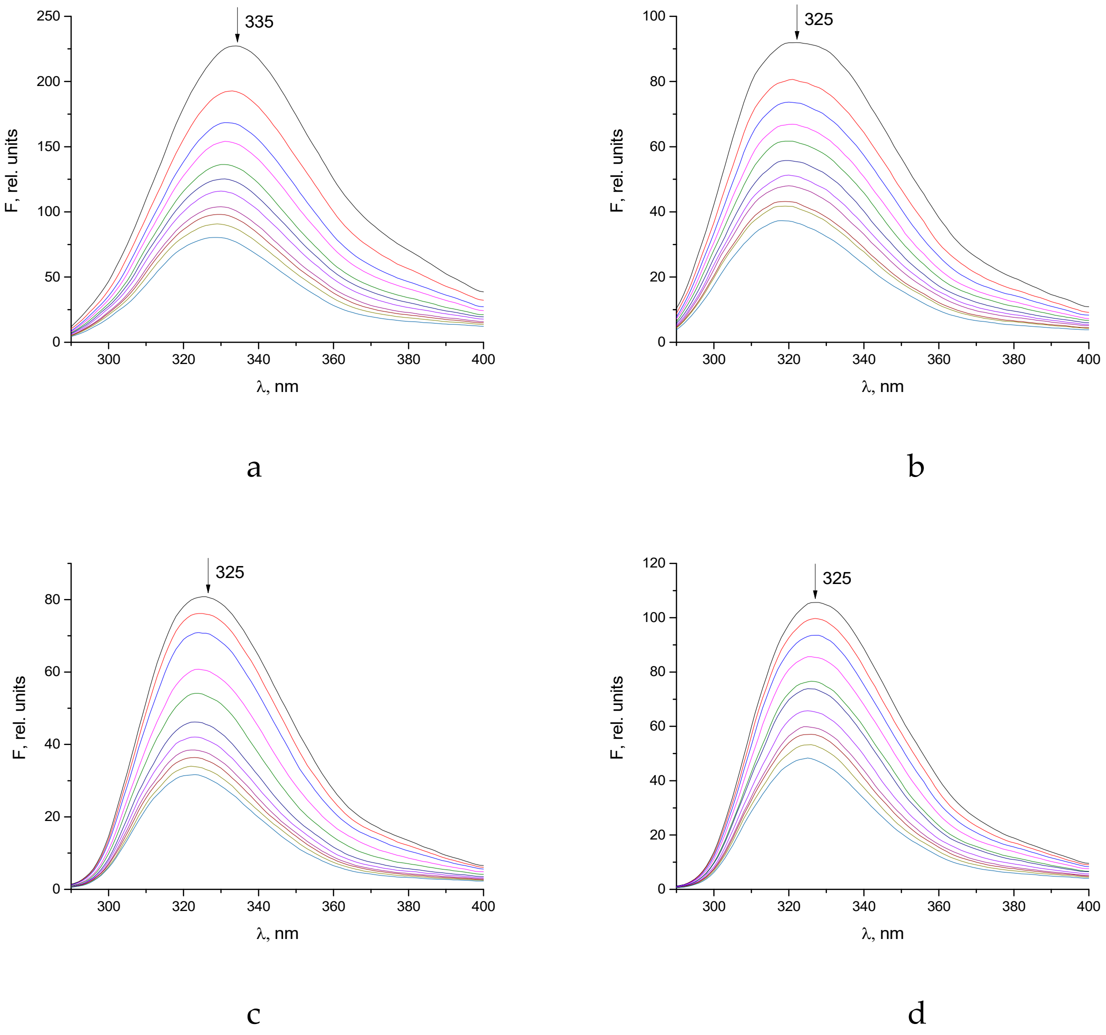

Figure S17. Inner filter-corrected fluorescent spectra of bovine serum albumin (a), human serum albumin (b), bovine γ-globulin (c), human γ-globulin (d) aqueous solutions (pH 6.86, T = 298.2 K, I = 0.05 M) titrated by hydrazone **5**

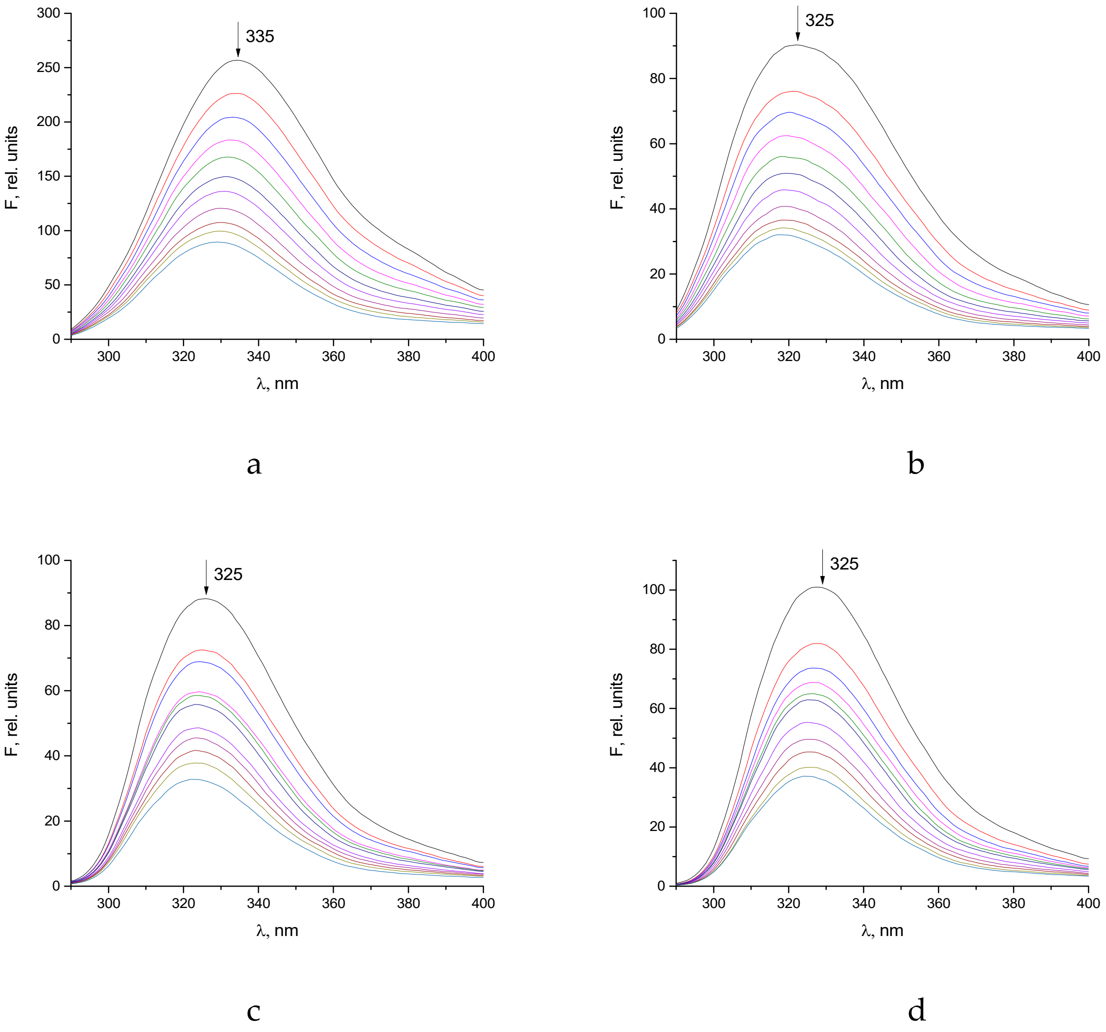

Figure S18. Inner filter-corrected fluorescent spectra of bovine serum albumin (a), human serum albumin (b), bovine γ-globulin (c), human γ-globulin (d) aqueous solutions (pH 6.86, T = 298.2 K, I = 0.05 M) titrated by hydrazone **6**

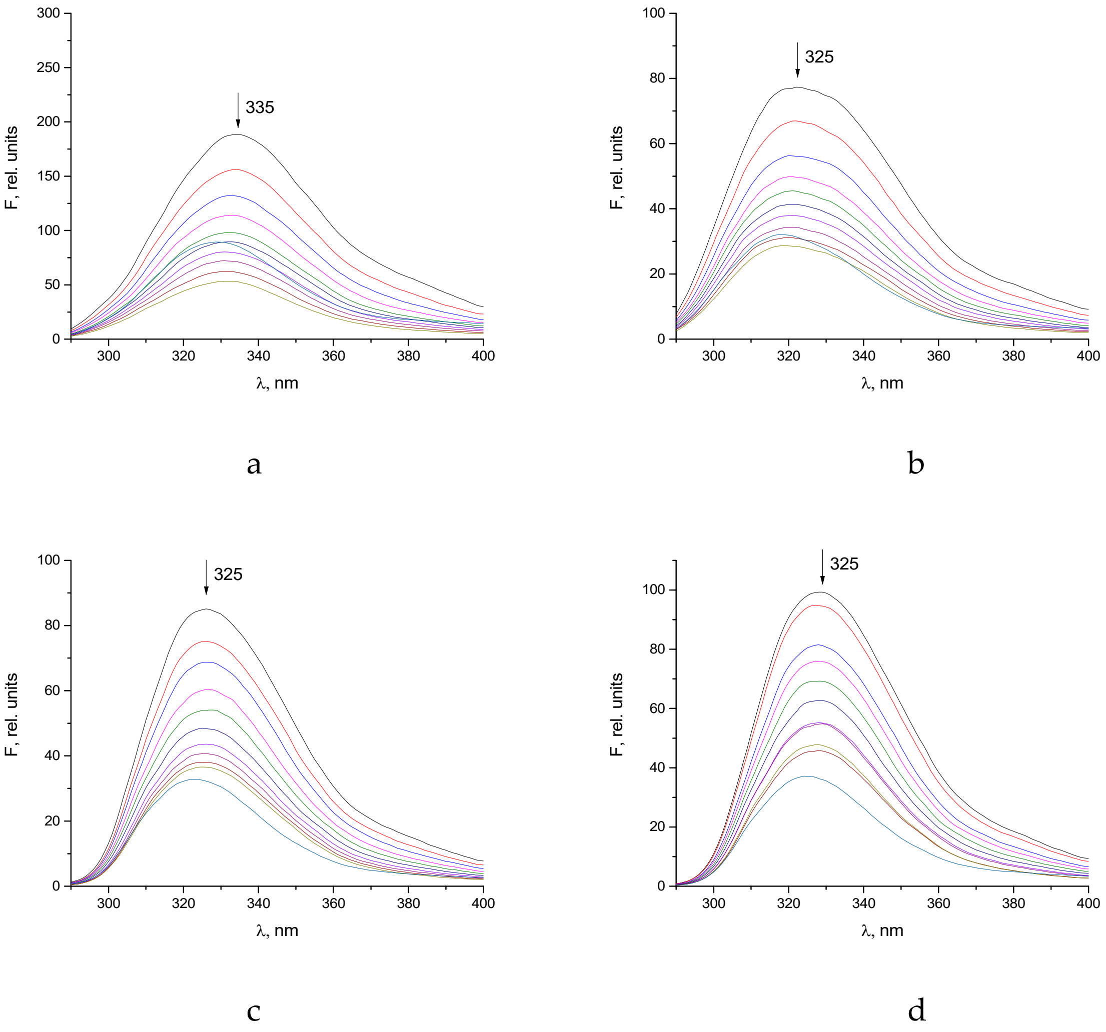

Figure S19. Inner filter-corrected fluorescent spectra of bovine serum albumin (a), human serum albumin (b), bovine γ-globulin (c), human γ-globulin (d) aqueous solutions (pH 6.86, T = 298.2 K, I = 0.05 M) titrated by hydrazone **7**

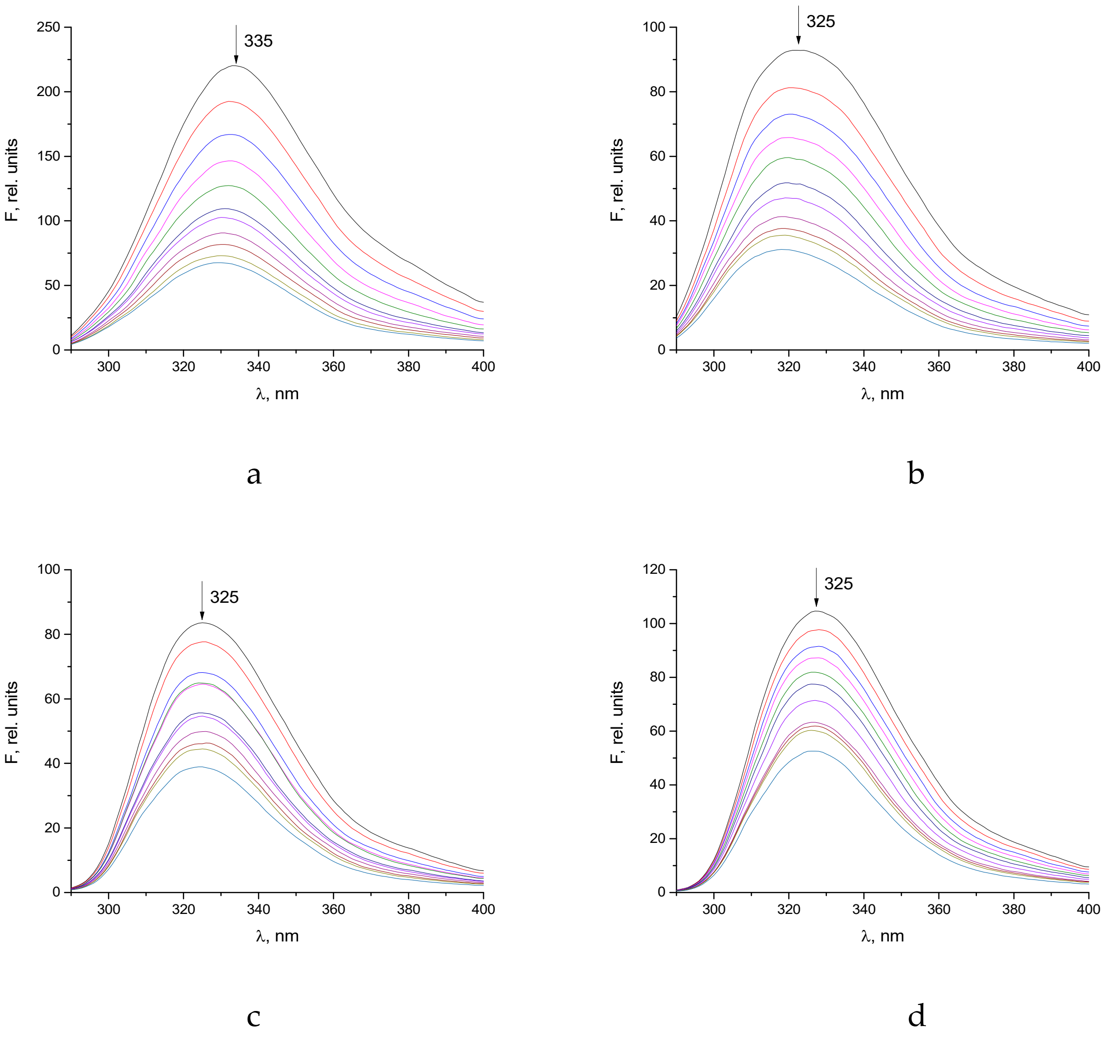

Figure S20. Inner filter-corrected fluorescent spectra of bovine serum albumin (a), human serum albumin (b), bovine γ-globulin (c), human γ-globulin (d) aqueous solutions (pH 6.86, T = 298.2 K, I = 0.05 M) titrated by hydrazone **8**

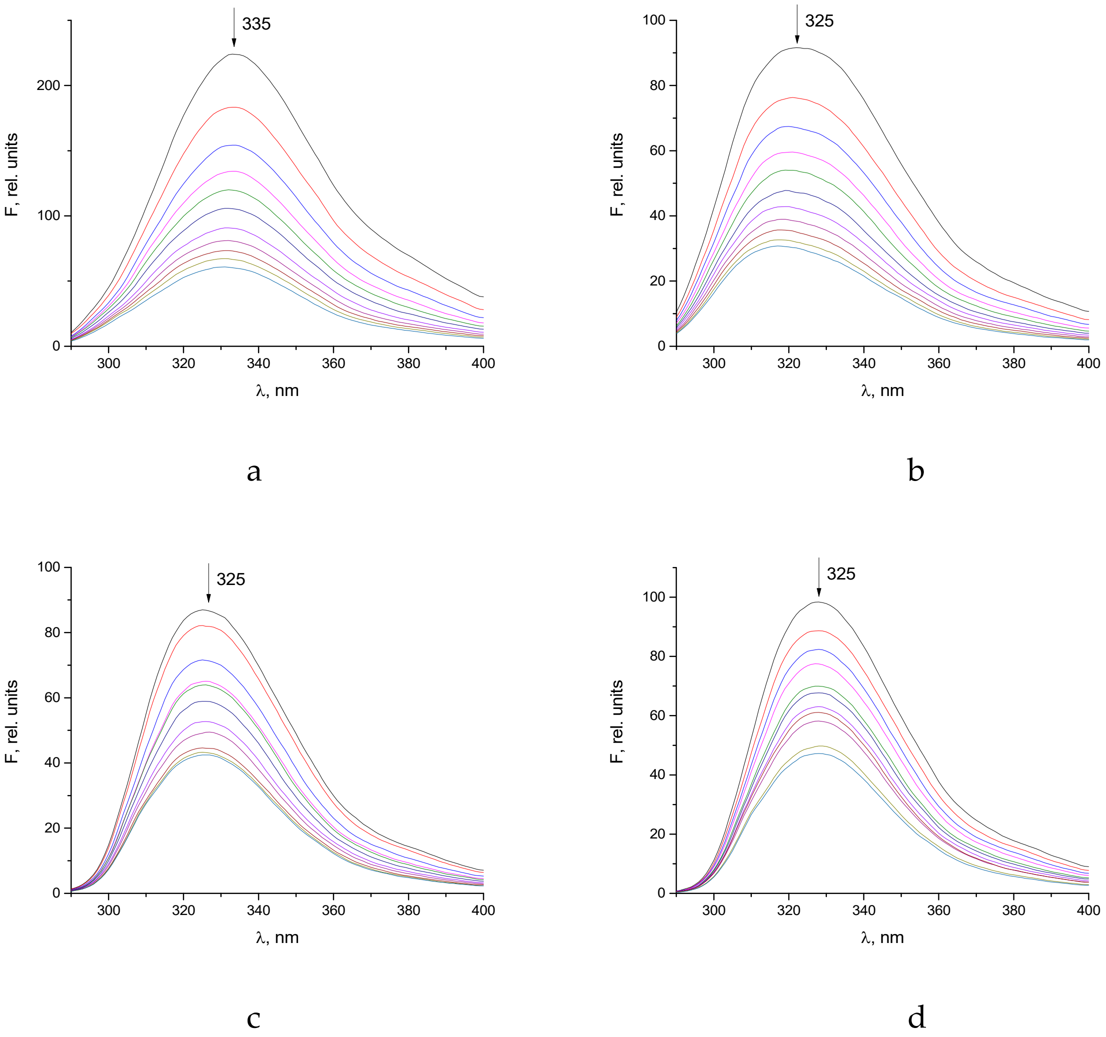

Figure S21. Inner filter-corrected fluorescent spectra of bovine serum albumin (a), human serum albumin (b), bovine γ-globulin (c), human γ-globulin (d) aqueous solutions (pH 6.86, T = 298.2 K, I = 0.05 M) titrated by hydrazone **9**

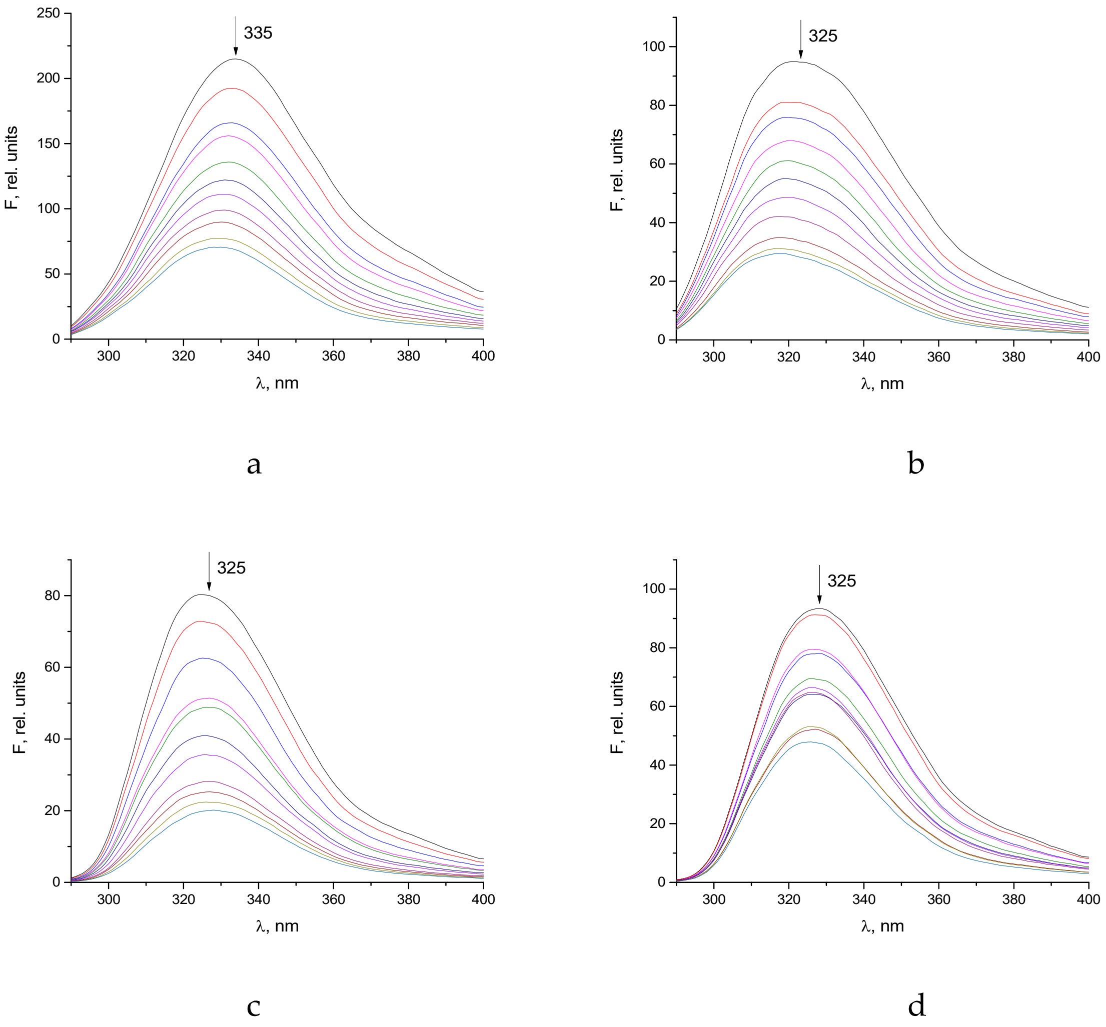

Figure S22. Inner filter-corrected fluorescent spectra of bovine serum albumin (a), human serum albumin (b), bovine γ-globulin (c), human γ-globulin (d) aqueous solutions (pH 6.86, T = 298.2 K, I = 0.05 M) titrated by hydrazone **10**

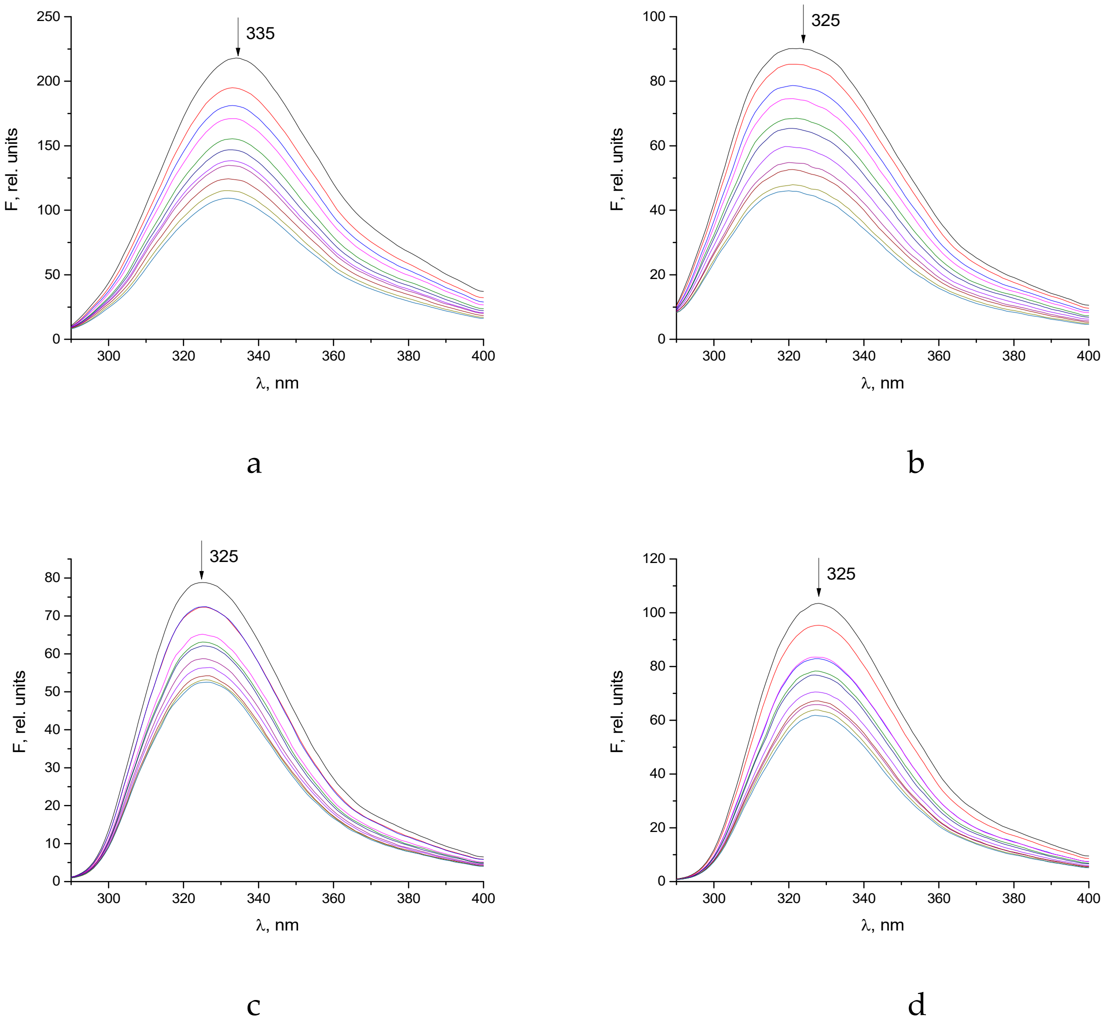

Figure S23. Inner filter-corrected fluorescent spectra of bovine serum albumin (a), human serum albumin (b), bovine γ-globulin (c), human γ-globulin (d) aqueous solutions (pH 6.86, T = 298.2 K, I = 0.05 M) titrated by hydrazone **11**

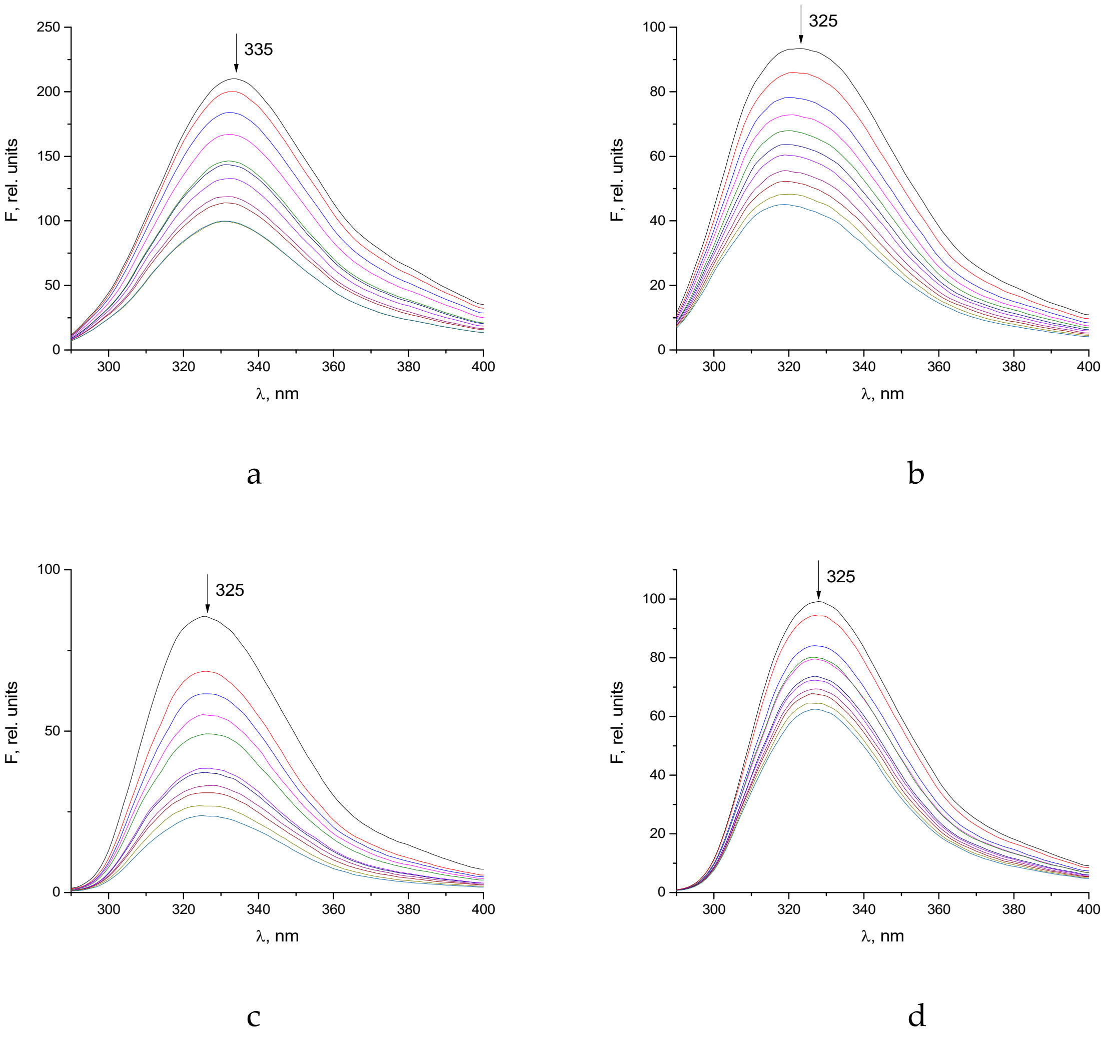

Figure S24. Inner filter-corrected fluorescent spectra of bovine serum albumin (a), human serum albumin (b), bovine γ-globulin (c), human γ-globulin (d) aqueous solutions (pH 6.86, T = 298.2 K, I = 0.05 M) titrated by hydrazone **12**

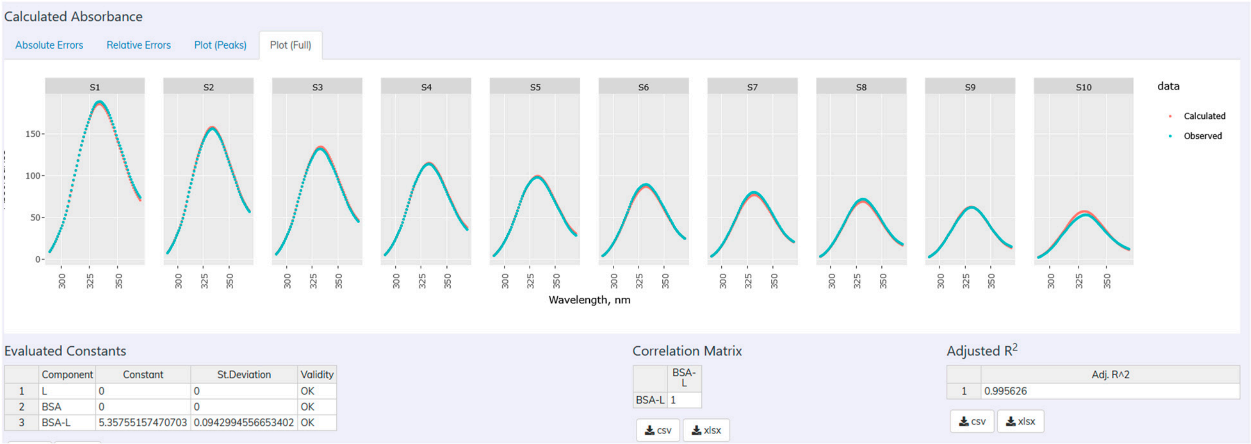

a

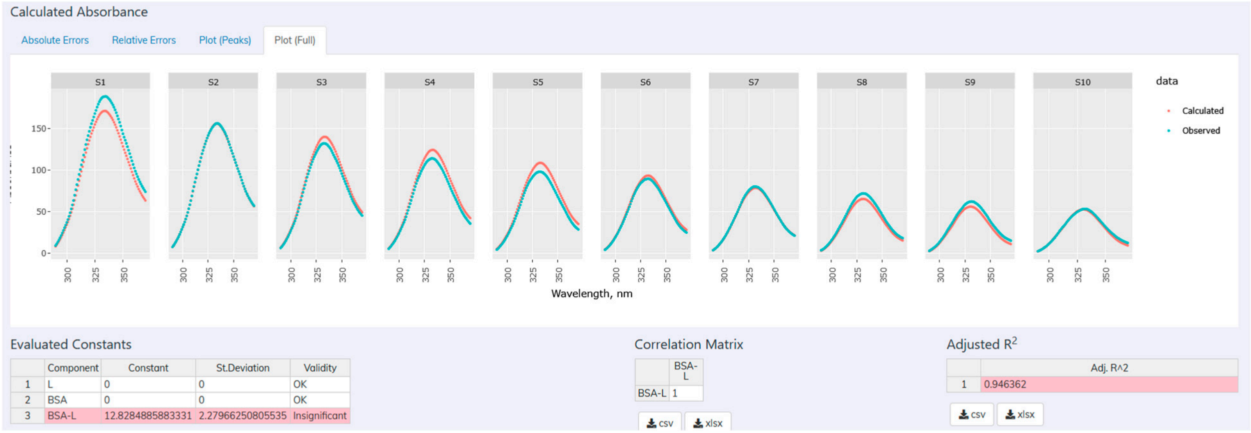

b

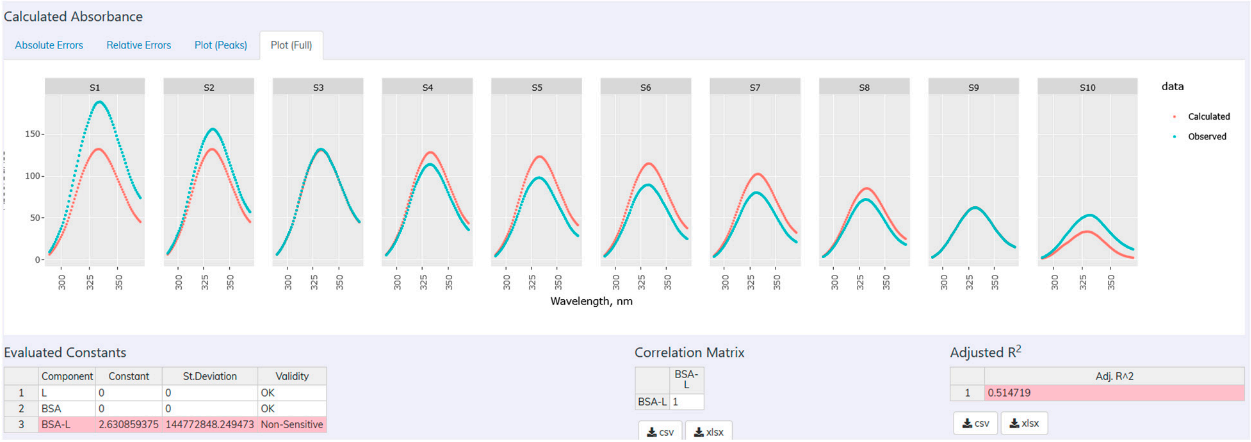

c

Figure S25. Testing of different stoichiometric models for the system of bovine serum albumin (P) + hydrazone **7** (L): a) P:L = 1:1; b) P:L = 1:2; c) P:L = 1:3

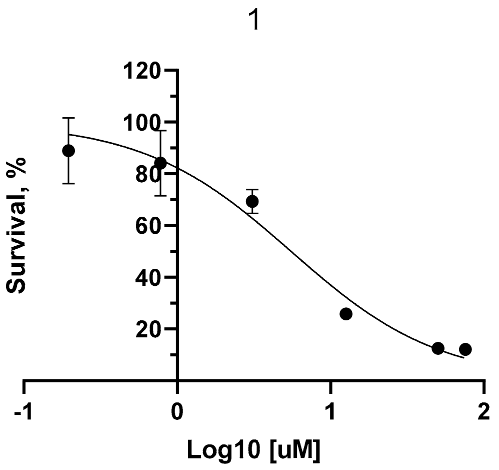
1
Survival, %
Log10 [uM]
120
100
80
60
40
20
-1
0
1
2
a

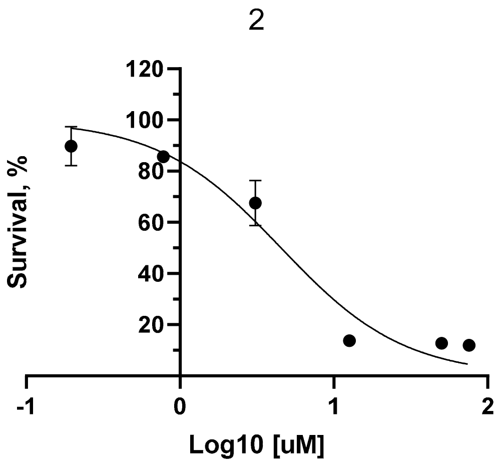
2
Survival, %
Log10 [uM]
120
100
80
60
40
20
-1
0
1
2
b

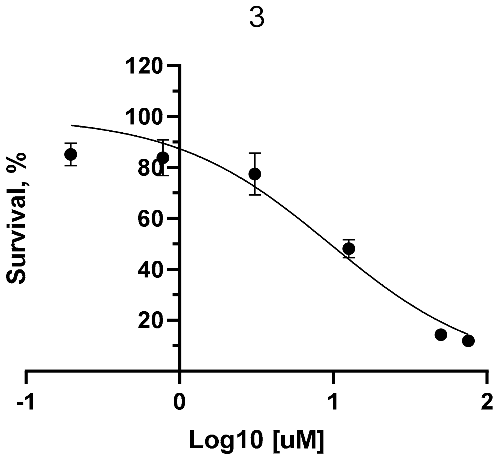
3
Survival, %
Log10 [uM]
120
100
80
60
40
20
-1
0
1
2
c

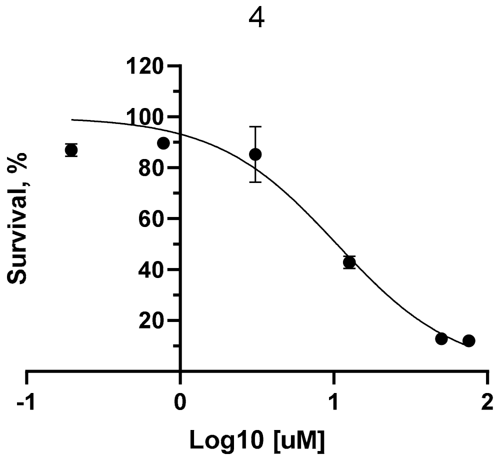
4
Survival, %
Log10 [uM]
120
100
80
60
40
20
-1
0
1
2
d

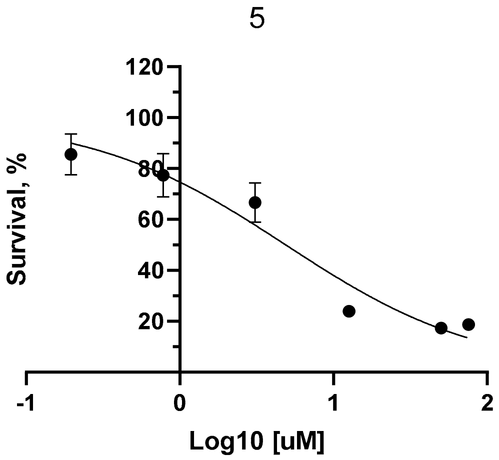
5
Survival, %
Log10 [uM]
120
100
80
60
40
20
-1
0
1
2
e

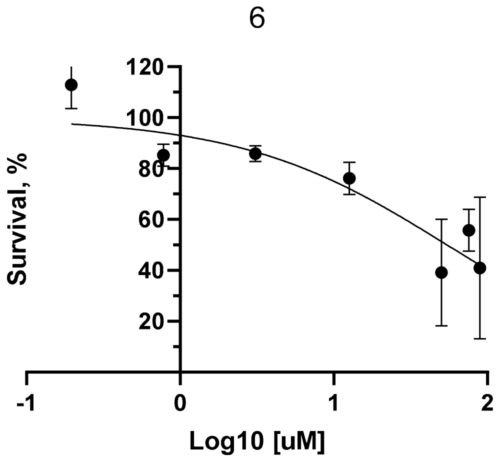
6
Survival, %
Log10 [uM]
120
100
80
60
40
20
-1
0
1
2
f

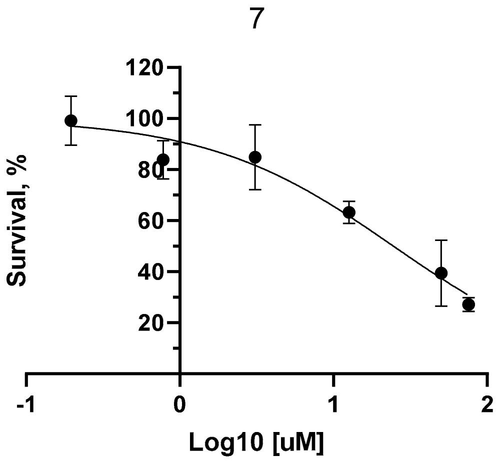
7
Survival, %
120
100
80
60
40
20
-1
0
1
2
Log10 [uM]

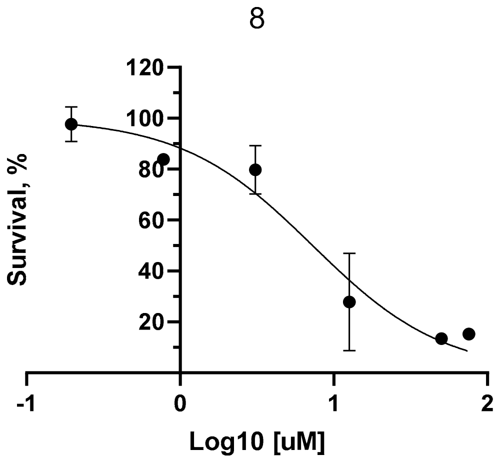
8
Survival, %
120
100
80
60
40
20
-1
0
1
2
Log10 [uM]

g

h

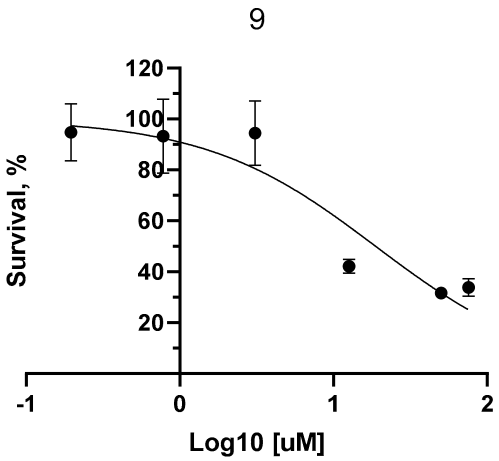
9
Survival, %
120
100
80
60
40
20
-1
0
1
2
Log10 [uM]

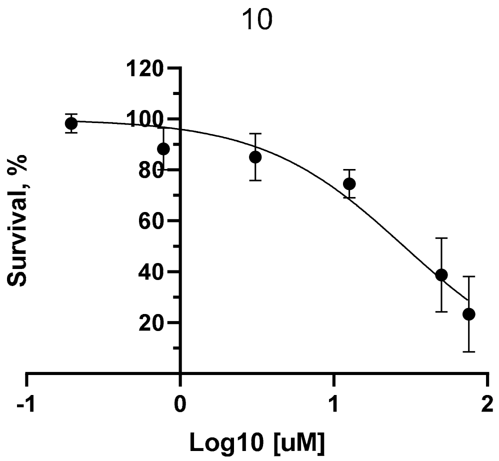
10
Survival, %
120
100
80
60
40
20
-1
0
1
2
Log10 [uM]

i

j

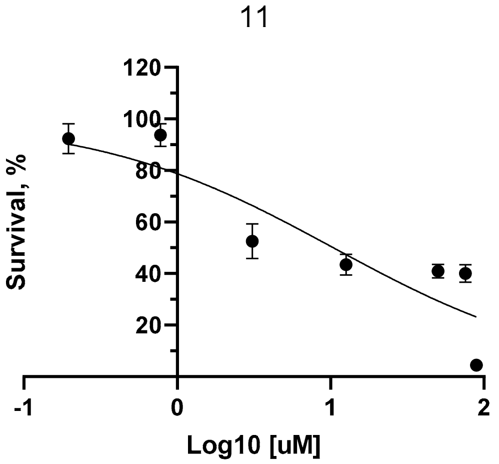
11
Survival, %
120
100
80
60
40
20
-1
0
1
2
Log10 [uM]

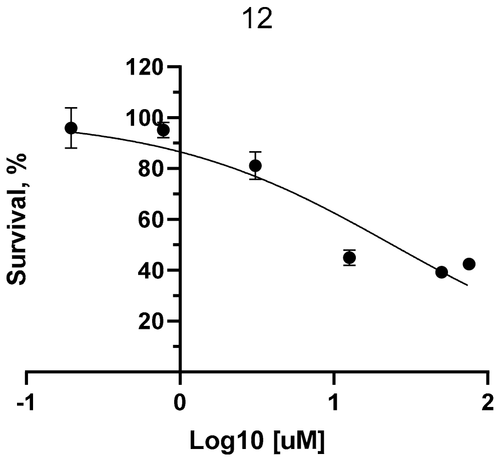
12
Survival, %
120
100
80
60
40
20
-1
0
1
2
Log10 [uM]

k

l

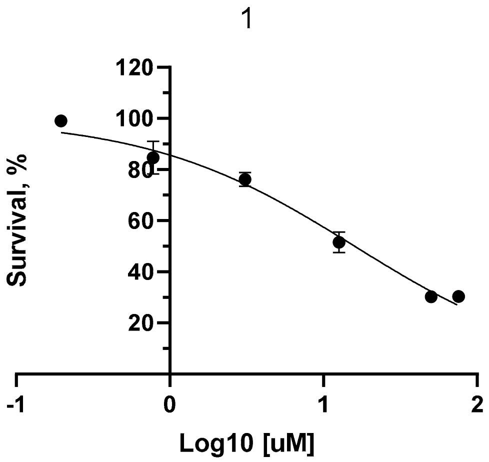
1
120
100
80
60
40
20
Survival, %
-1
0
1
2
Log10 [uM]

m

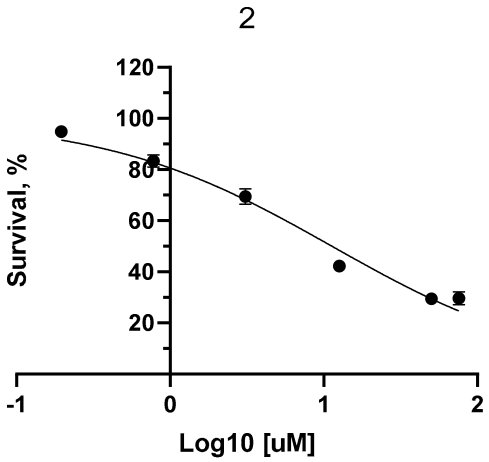
2
120
100
80
60
40
20
Survival, %
-1
0
1
2
Log10 [uM]

n

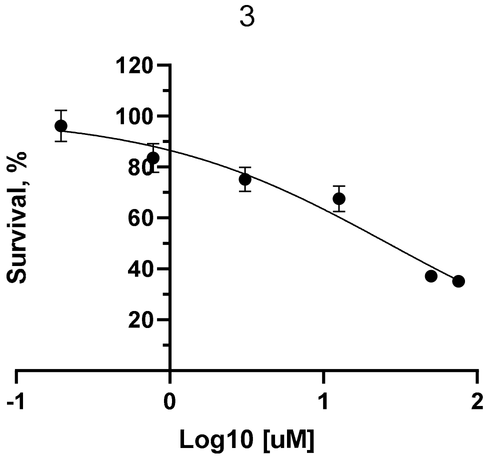
3
120
100
80
60
40
20
Survival, %
-1
0
1
2
Log10 [uM]

o

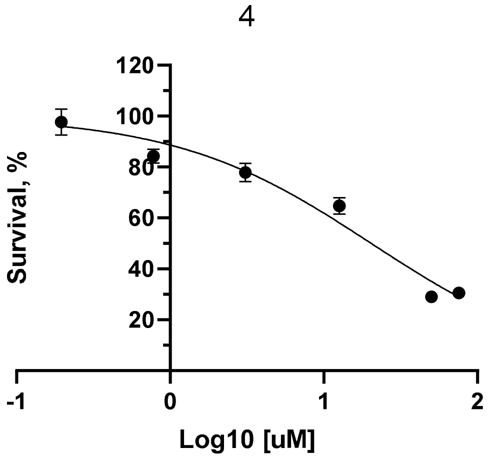
4
120
100
80
60
40
20
Survival, %
-1
0
1
2
Log10 [uM]

p

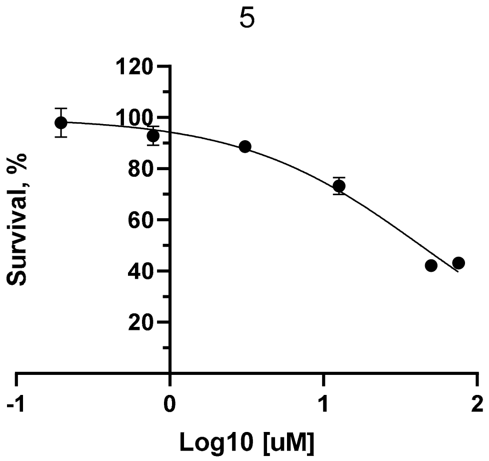
5
120
100
80
60
40
20
Survival, %
-1
0
1
2
Log10 [uM]

q

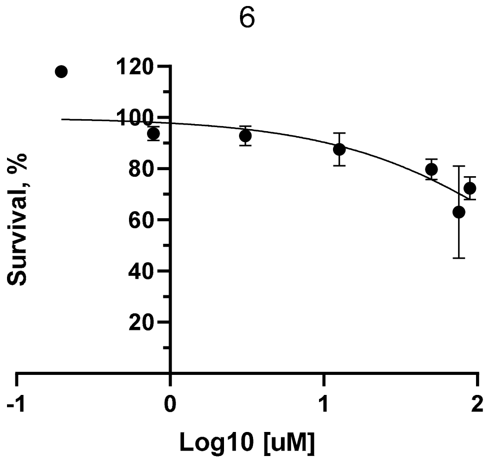
6
120
100
80
60
40
20
Survival, %
-1
0
1
2
Log10 [uM]

r

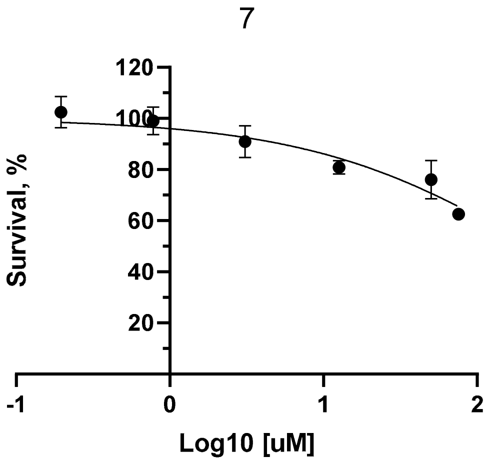
7
Survival, %
Log10 [uM]
120
100
80
60
40
20
-1
0
1
2

s

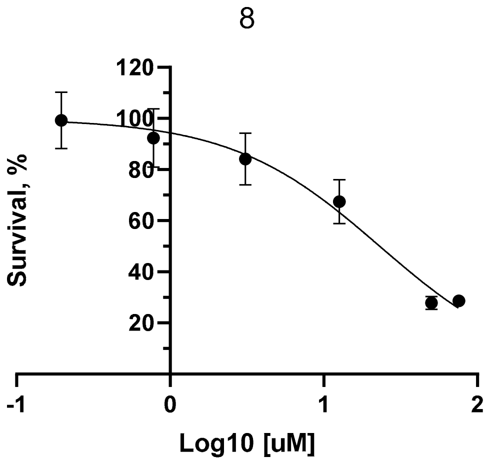
8
Survival, %
Log10 [uM]
120
100
80
60
40
20
-1
0
1
2

t

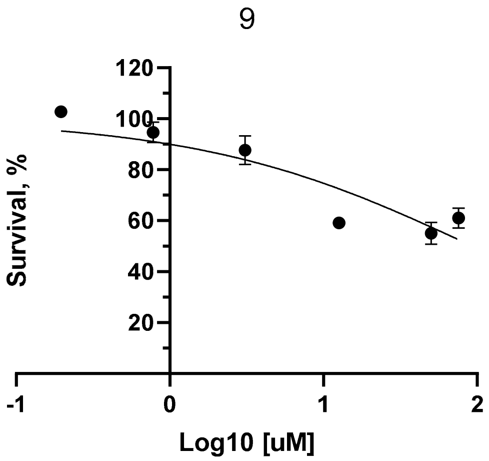
9
Survival, %
Log10 [uM]
120
100
80
60
40
20
-1
0
1
2

u

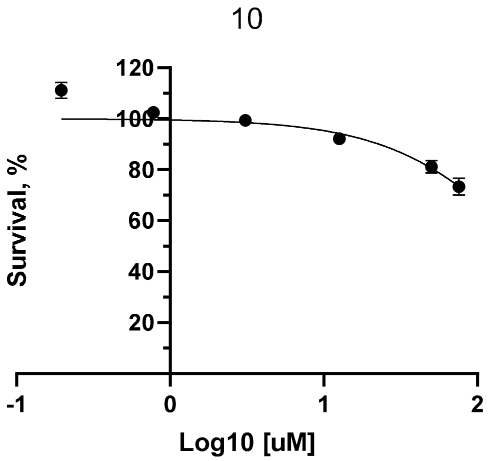
10
Survival, %
Log10 [uM]
120
100
80
60
40
20
-1
0
1
2

v

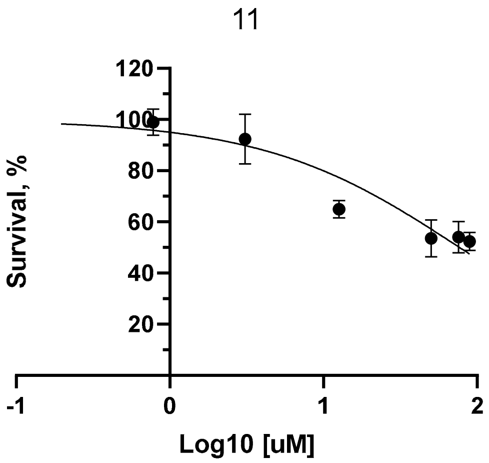
11
Survival, %
Log10 [uM]
120
100
80
60
40
20
-1
0
1
2

w

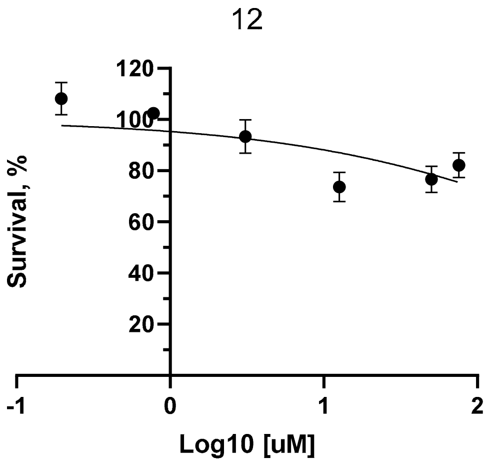
12
Survival, %
Log10 [uM]
120
100
80
60
40
20
-1
0
1
2

x

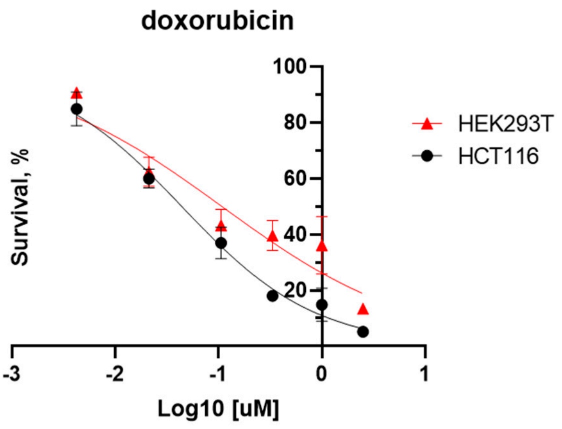

y

Figure S26. Dose-response curves for HCT116 (a-l) and HEK293 (m-x) cell lines treated by hydrazones **1** (a, m), **2** (b, n), **3** (c, o), **4** (d, p), **5** (e, q), **6** (f, r), **7** (g, s), **8** (h, t), **9** (i, u), **10** (j, v), **11** (k, w), **12** (l, x) and positive control, doxorubicin (y). Three independent experiments were performed to fit the curves and calculate IC_{50} means

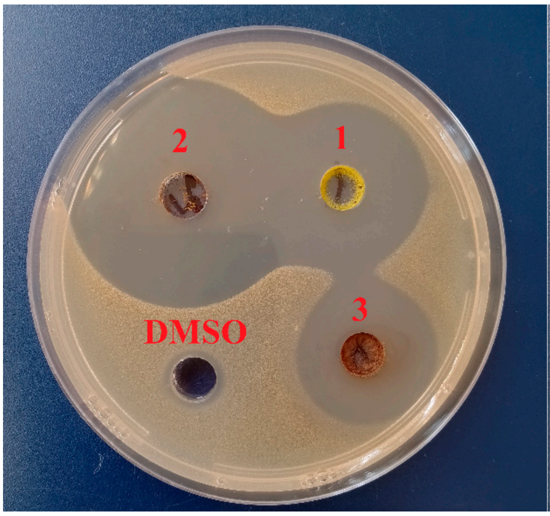

a

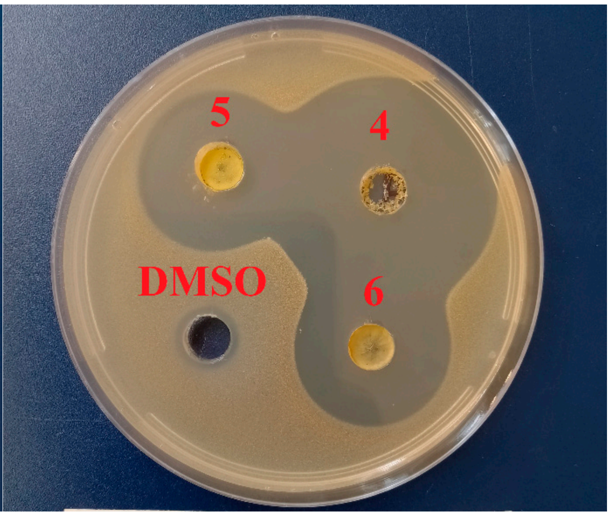

b

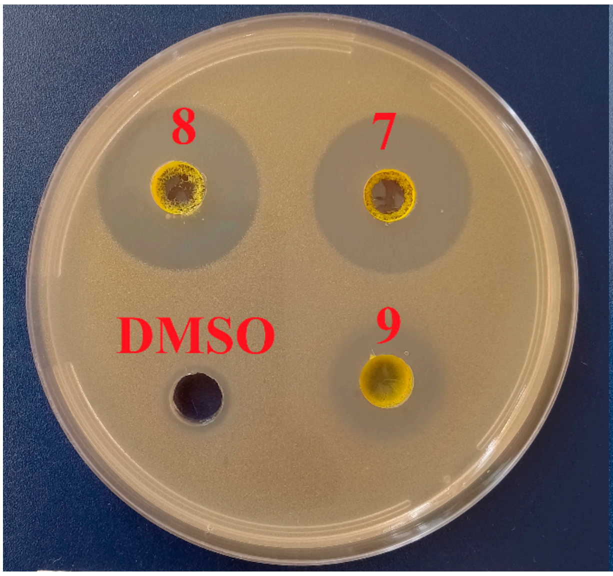

c

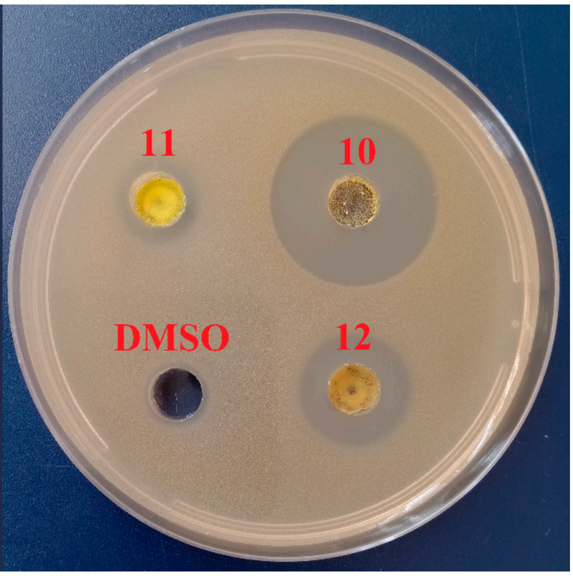

d

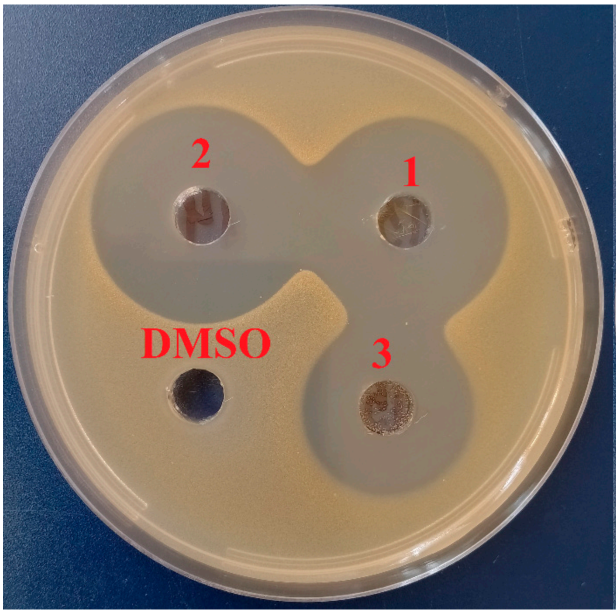

e

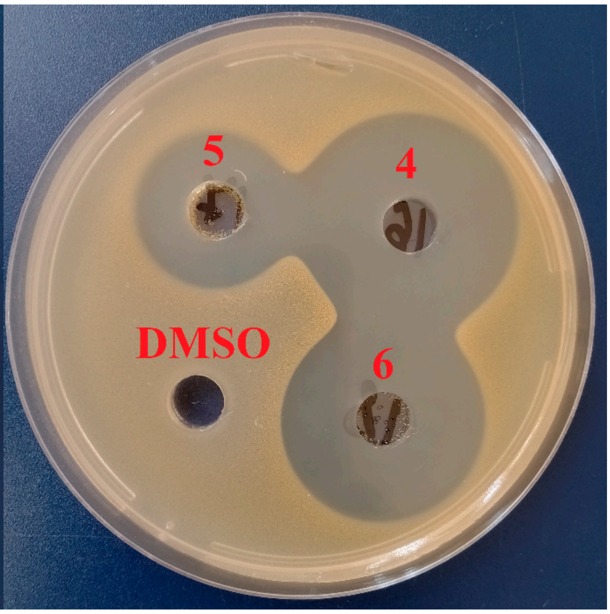

f

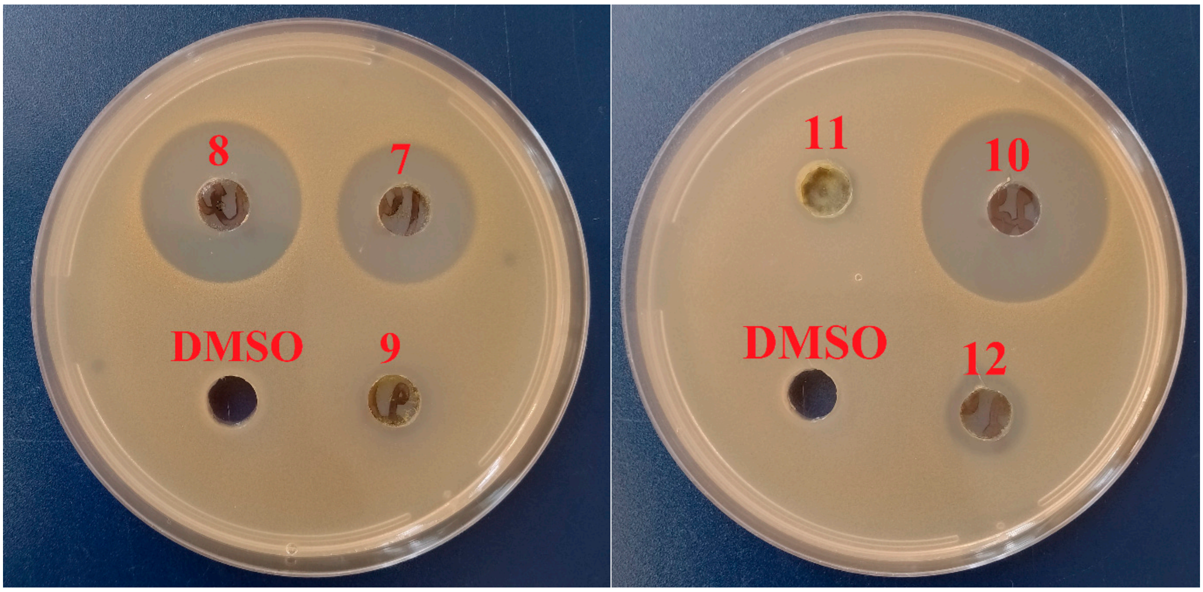

g h

Figure S27. Petri dishes containing *S. aureus* treated by hydrazones **1-12**. Concentrations of hydrazones **1-12** are 10 µg mL^{-1} (a-d) and 1 µg mL^{-1} (e-h)

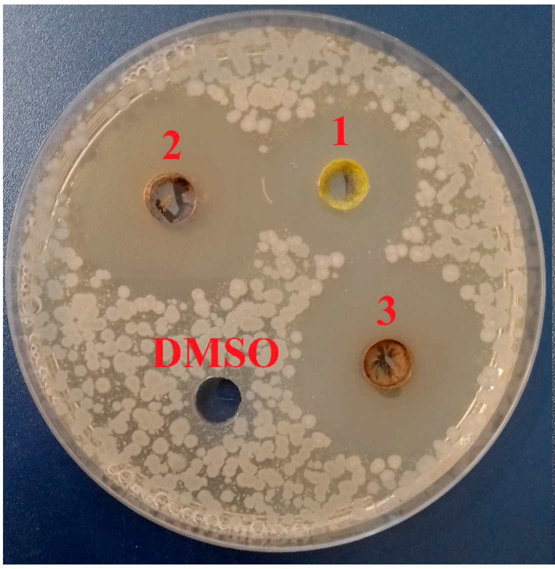

a

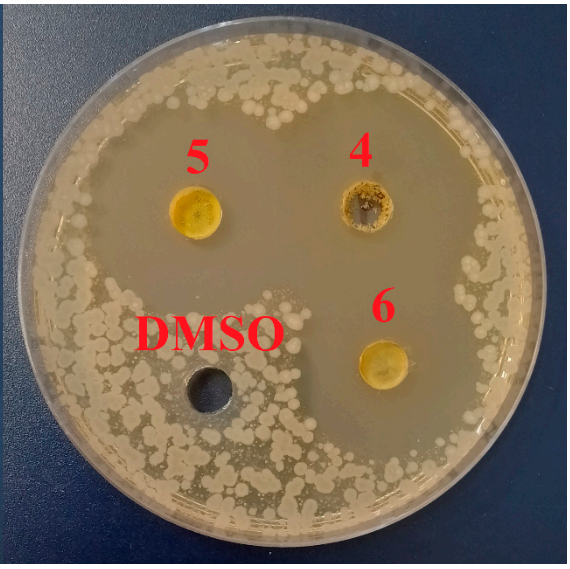

b

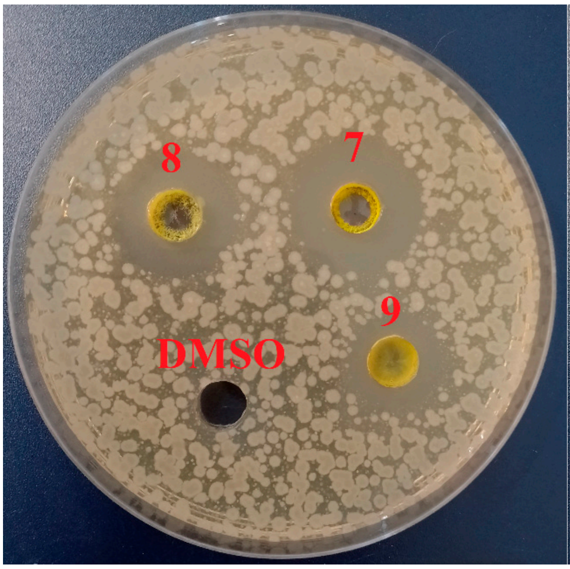

c

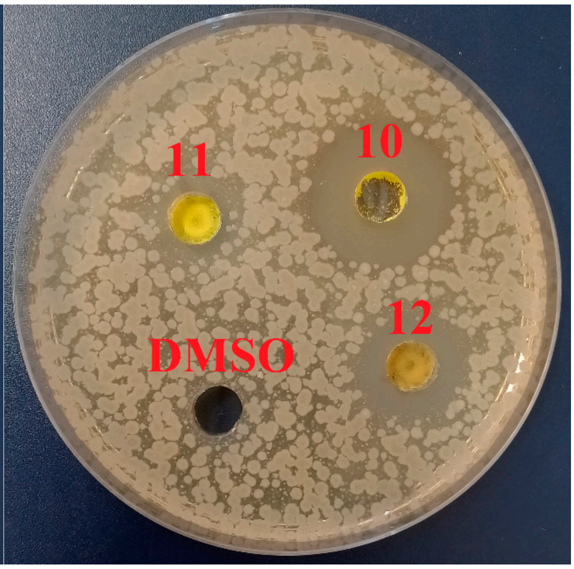

d

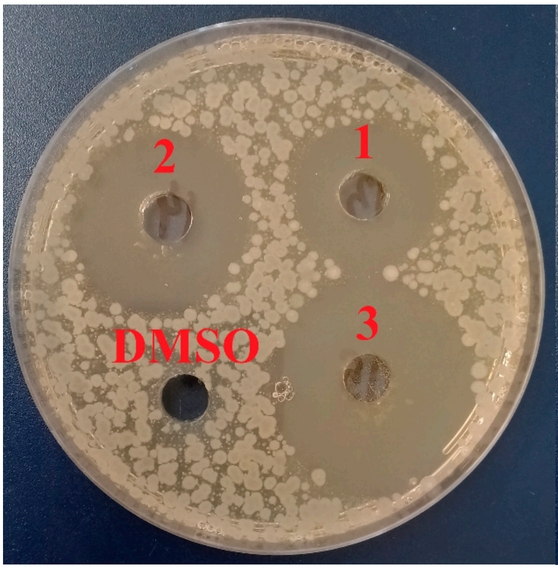

e

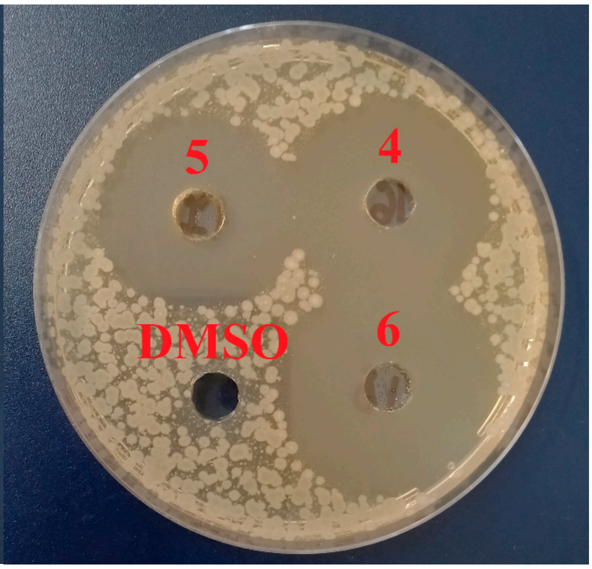

f

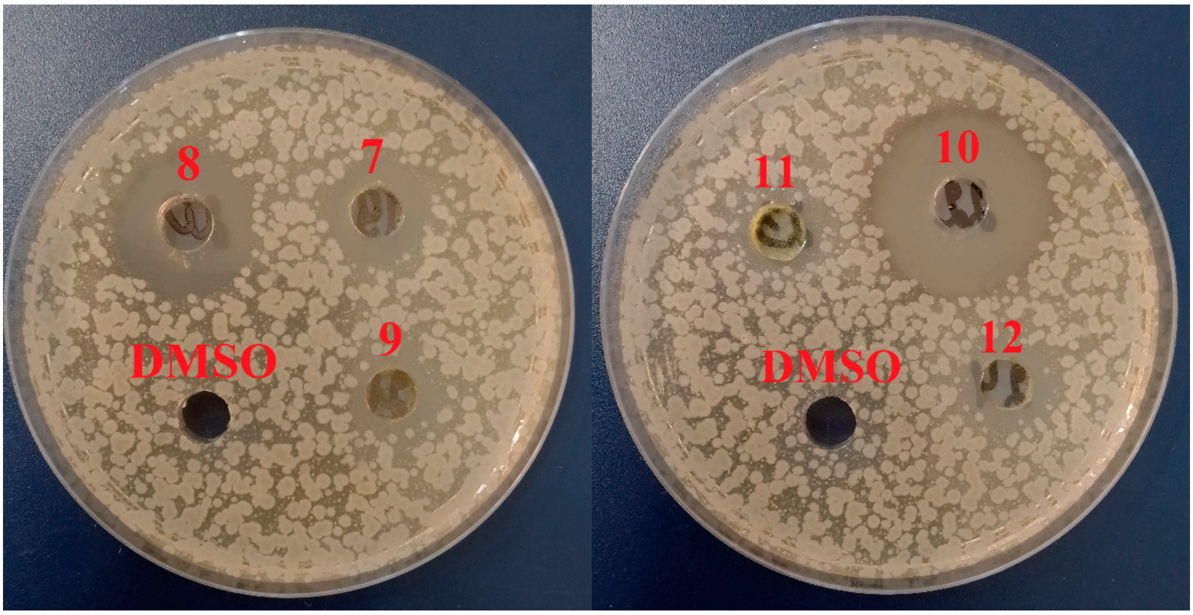

g h

Figure S28. Petri dishes containing *B. subtilis* treated by hydrazones **1-12**. Concentrations of hydrazones **1-12** are 10 μg mL^{-1} (a-d) and 1 μg mL^{-1} (e-h)

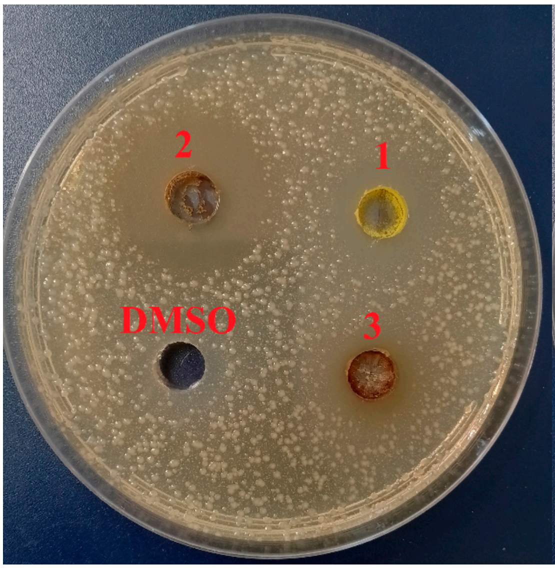

a

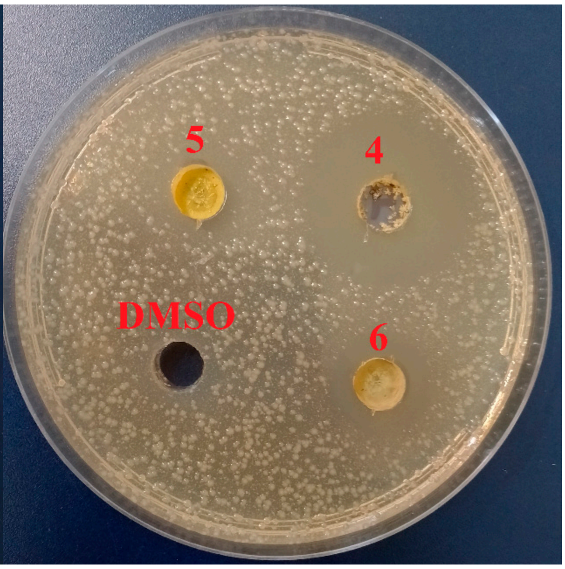

b

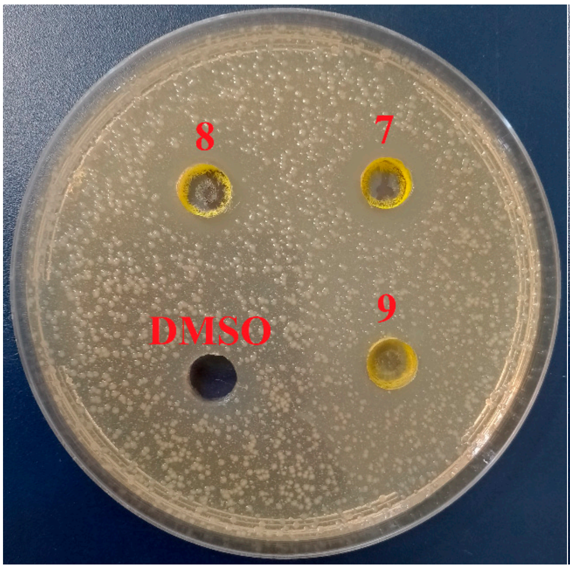

c

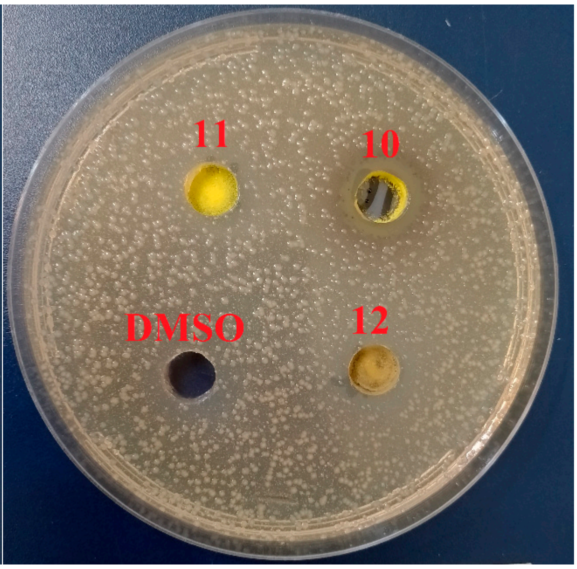

d

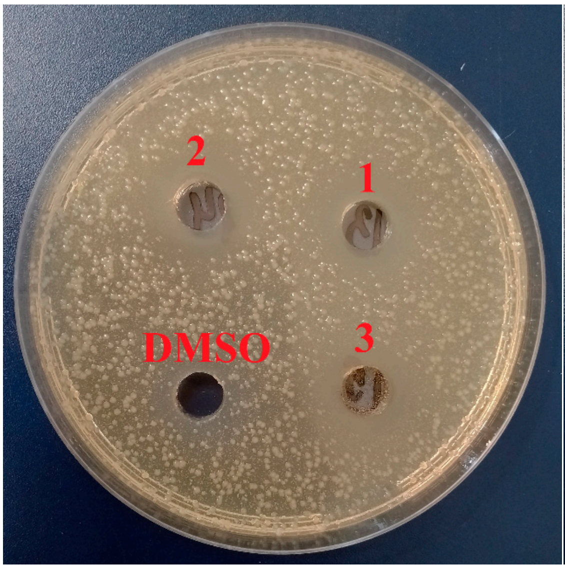

e

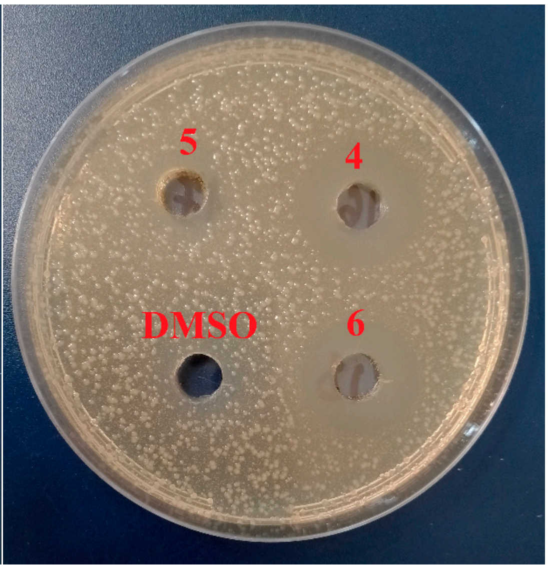

f

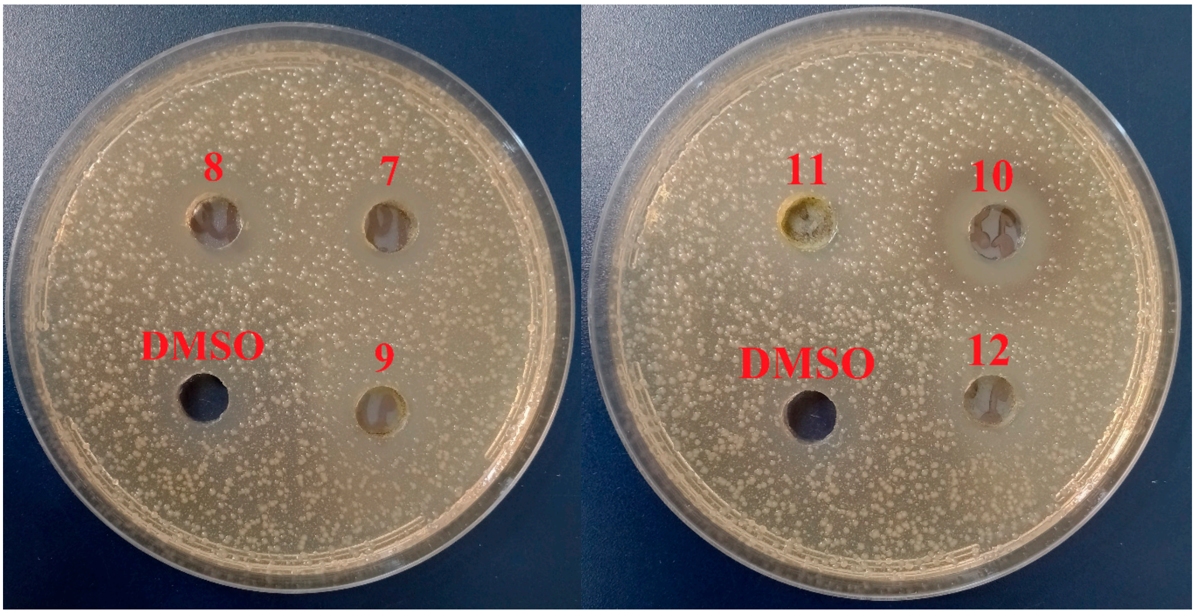

g h

Figure S29. Petri dishes containing *E. coli* treated by hydrazones **1-12**. Concentrations of hydrazones **1-12** are 10 μg mL^{-1} (a-d) and 1 μg mL^{-1} (e-h)
